# Scalable Membrane Enabled One-Pot Liquid-Phase Oligonucleotide Synthesis

**DOI:** 10.1021/acs.oprd.5c00117

**Published:** 2025-05-13

**Authors:** Ronan Kelly, Catalina Parga, Steven Ferguson

**Affiliations:** † Department of Chemical and Bioprocess Engineering, 8797 University College Dublin, D04 V1W8 Dublin 4, Ireland; ‡ SSPC, The Research Ireland Centre for Pharmaceuticals, School of Chemical and Bioprocess Engineering, 8797 University College Dublin, D04 V1W8 Dublin 4, Ireland; § National Institute for Bioprocess Research and Training, Foster Avenue, Mount Merrion, Blackrock, Co. Dublin A94 X099, Ireland

**Keywords:** oligonucleotide therapeutics, membrane, liquid-phase oligonucleotide synthesis, organic solvent, nanofiltration, ultrafiltration

## Abstract

In this article, a new one-pot liquid-phase oligonucleotide synthesis (OP-LPOS) route enabled by organic solvent resistant (OSR) ceramic membranes is described. This approach was demonstrated through the synthesis of 6mer and 18mer 2’-OMe phosphorothioate oligonucleotides with high stepwise filtration yields (97–100%), and high crude purity (∼72% for 18mer) using just 1.5 equiv of phosphoramidites. Ceramic organic solvent nanofiltration (OSN) and ultrafiltration (OSU) membranes were used to selectively retain the growing oligonucleotide, which is reversibly tethered to a 4-arm branched PEG support, facilitating lower molecular weight reaction byproducts to permeate to waste. This is the first application of ceramic ultrafiltration membranes in such an application, which enables purification of intermediate products in just 5 diavolumes with high permeance (13 Lm^–2^ h^–1^ bar^–1^). We employ a one-pot approach that integrates sequential coupling, sulfurization, and detritylation steps, followed by a single membrane purification step per chain extension cycle. Analysis of the methodology indicates that the homogeneous reactions and separation performance, which use commercially available reagents and highly scalable membrane systems, represent a promising alternative to solid-phase oligonucleotide synthesis (SPOS) for large-scale manufacturing of therapeutic oligonucleotides. Furthermore, the combination of OP-LPOS with membrane separation increases intermediate product purity and yield. It reduces the number of unit operations, cycle times, and process mass intensity (PMI) compared to the previous state-of-the-art membrane-based LPOS.

## Graphical abstract

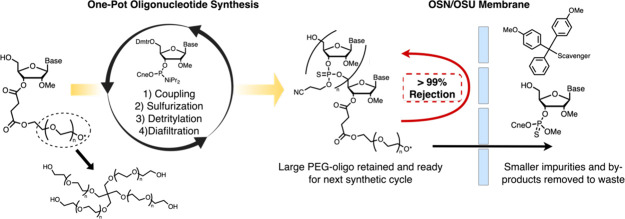

## 1 Introduction

The number of therapeutic oligonucleotides (∼15–25 nucleotides) on the market and in clinical trials has increased gradually over the last two decades, but little change has been seen in their commercial synthesis.^1^ Until recently, the demand for oligonucleotides has been fulfilled by traditional solid-phase oligonucleotide synthesis (SPOS), which suffers from scalability issues with batch sizes limited to approximately 10 kg due to increasing column sizes, resulting in nonlinear flow rates and reduced product purity.^2–4^ SPOS requires large excesses of solvents and reagents, notably the expensive phosphoramidite monomers, to drive reactions to completion, meaning the process is highly mass-intensive at a large scale (process mass intensity (PMI) ∼4300 kg/kg for a 20mer oligo).^3^ Additionally, there is no possibility for in-line analysis of the product without cleavage from the support.^3,5^

Liquid-phase oligonucleotide synthesis (LPOS) presents an attractive alternative to SPOS as a scalable and potentially more sustainable option. LPOS offers major advantages as reactions in solution proceed faster due to a higher rate of mass transfer, which facilitates the use of a small excess of reagents, standard batch reactors can be used rather than specialized solid-phase synthesizers, and a sample can be taken at any stage during the experiment to monitor and optimize each step.^2–4^ In recent years, researchers have opted for telescoped one-pot synthesis strategies, with sequential coupling, oxidation/sulfurizatio,n and deprotection, which can reduce solvent consumption by reducing the number of separation steps.^3,6–9^

To date, most of the published research on LPOS has focused on generating small quantities of oligonucleotides to solve intermediate purification issues using precipitation, membrane filtration, or liquid–liquid extraction.^10–12^ In LPOS, oligonucleotides are reversibly tethered to a soluble support, which facilitates easier separation of the growing oligonucleotides from reaction debris. High efficiency liquid-phase (HELP) oligonucleotide synthesis by Bonora et al. was an early example of LPOS using a linear polyethylene glycol (PEG) support, with product isolation by precipitation used to extend the sequence.^5,10,13,14^ More recently, Ajinomoto (AJIPHASE) and Biogen have produced therapeutic oligonucleotides on a kilogram scale via precipitation using low molecular weight alkyl chain supports.^6,7,15^

The majority of LPOS applications use precipitation as a means for intermediate separation.^2,6,10,15–17^ However, there are drawbacks to precipitation as a unit operation for LPOS, as scaling up industrial precipitation may be limited by the extensive process development required to control particle size and precipitation rate, which may vary depending on the target compound.^18,19^ Membrane-assisted ‘tide’ synthesis was first proposed by Bayer and Mutter for the synthesis of peptides, which they later expanded to oligonucleotides.^20,21^ More recently, Livingston and co-workers demonstrated oligonucleotide synthesis using purification by organic solvent nanofiltration (OSN), which they call “nanostar sieving” and is proposed to be highly scalable.^12,22^ This membrane-based approach works by tethering the growing oligonucleotide to a 3-arm branched PEG support (nanostar), which was highly rejected by a polybenzimidazole (PBI) based membrane, facilitating clean separation from smaller reagents and byproducts, which permeate through the membrane to waste. Several challenges were identified, including a high PMI for the process, mostly due to diafiltration solvent consumption, a low overall process yield (39% for 9mer) due to insufficient product rejection, poor PEG-oligo solubility during chain extension, and incomplete removal of detritylation byproducts, requiring an additional precipitation step. More recently, Yeo et al. have published a liquid-phase peptide synthesis (LPPS) method via one-pot nanostar sieving (PEPSTAR) using a similar PBI membrane.^23^ These efforts have continued through Exactmer, a company that continues to develop this approach.^24,25^

Many organic solvent-resistant (OSR) polymeric and ceramic membranes are available commercially.^26^ In particular, ceramic membranes possess superior chemical resistance compared to polymeric membranes, which are prone to swelling.^17^ Only preliminary work has been conducted using ceramic membranes with LPOS, but these have found success in analogous liquid-phase peptide synthesis (LPPS).^27^ Ceramic Inopor NF 450 Da membranes were used for kilo-scale nanofiltration of synthetic peptides by Eli Lilly using a hybrid approach where fragments were synthesized on solid-phase and assembled in liquid-phase.^29^ Membrane enhanced peptide synthesis (MEPS) was introduced by So et al., which used a hydrophobic modified Inopor UF 2000 Da membrane with a linear 5 kDa methoxy PEG (mPEG) support to synthesize peptides using liquid-phase reactions.^30^ Later novel globular branched PEG supports (∼6–8 kDa) were introduced to improve MEPS, using smaller pore Inopor NF 450 and 750 Da membranes.^31^

The motivation for this work was to develop a scalable membrane-assisted LPOS platform using commercially available membranes, supports, and reagents to encourage wider adoption. We report the integration of a one-pot liquid-phase oligonucleotide synthesis (OP-LPOS) strategy with commercially available ceramic membranes (Inopor NF 750 and UF 2000 Da). 4-arm PEG was used as a soluble support as it is readily available in a variety of sizes and shows close to quantitative membrane rejection with the correct membrane pore size. The developed platform was demonstrated through the synthesis of 6mer and 18mer 2′-OMe phosphorothioate oligonucleotides on a gram scale. The process is an improvement over current membrane-assisted LPOS work in terms of stepwise yield and purity, the number of unit operations, and process mass intensity (PMI). We propose that this approach is a potential alternative to SPOS for the large-scale manufacturing of therapeutic oligonucleotides (Figure 1).

**Figure 1:**
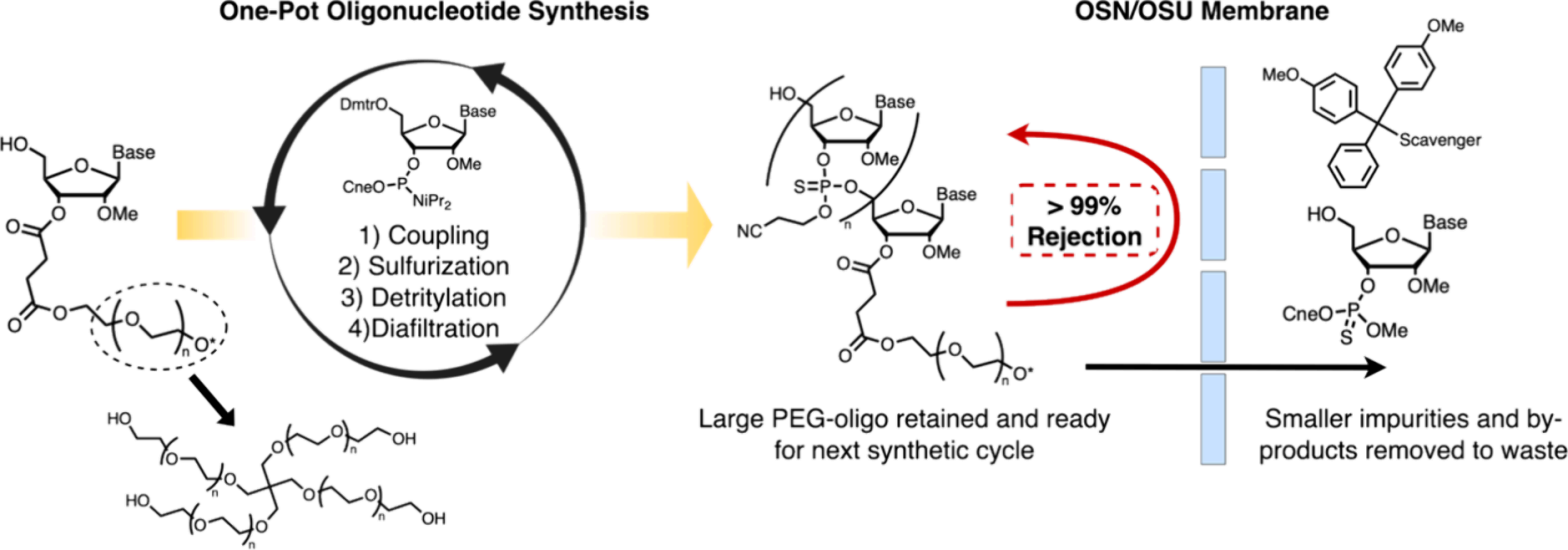
Schematic representation of scalable one-pot liquid-phase oligonucleotide synthesis (OP-LPOS) enhanced by organic solvent resistant (OSR) ultrafiltration/nanofiltration membrane separation.

## 2 Experimental Section

### 2.1 Membranes and Modules

Ceramic membranes were purchased from Inopor/Raushert Kloster Veilsdorf GmbH (Germany). Inopor NF 750 Da (1 nm pore, 750 Da MWCO) and UF 2000 Da (3 nm pore, 2000 Da MWCO) membranes were purchased as 1-channel membranes (AA0250-A3Z3G (UF), AA0600-A3T1G (NF), 10 mm diameter, 250 mm length), and the NF was also purchased as a 19-channel membrane (CA0250-A3T1G, 25 mm diameter, 250 mm length). The membranes were housed in modules (FG-M1–10 × 0250-PN40-SV, FG-M1–25 × 0250-PN40-SV) supplied by FRTJ GmbH. Further details and photos of the membrane and module are given in the Supporting Information.

### 2.2 Membrane Operation during Screening and Purification

Membrane filtration was performed in a cross-flow filtration unit made in-house (Figure 2), pressurized by an HPLC pump (Teledyne LD-Class). A gear pump (Micropump GB) provided flow up to 3.3 L/min. Crude reaction mixtures or screening material (i.e., PEG species or phosphoramidite; 1 g/L) were poured into the separation rig and pressurized to the desired pressure. Typically, NF 750 Da membranes are used at an operating pressure of 8–10 bar, and UF 2000 Da membranes are used at 5–8 bar. MeCN was used as the diafiltration solvent, with MeCN:MeOH (9:1 or 4:1 v/v) utilized at longer oligo chain lengths to counteract unfavorable changes in permeability and impurity rejection. A three-way valve was placed on the permeate side to direct flow during recycle or diafiltration modes. During recycle mode, the permeate line was directed back to the feed tank, and during diafiltration mode, it was directed toward waste. Once pressurized, membranes were left to reach steady-state permeance in recycle mode, usually 30–60 min, before beginning purification. Purification was operated in constant volume diafiltration (CVD) mode where a diaphragm pump (KNF SIMDOS 10 FEM 1.10 S) added fresh solvent to the feed tank to balance system volume loss from the permeate. The temperature during diafiltration was kept constant at room temperature (21 °C ± 3). Samples (0.1 mL) were taken from the feed tank and permeate at regular intervals and diluted with MeOH (1 mL) to monitor diafiltration by HPLC. To collect the purified product, the retentate and permeate lines were fed to a collection vessel, and fresh solvent was pumped through the system. Table 1 summarizes the membrane filtration conditions for the synthesis of oligonucleotides in this study.

**Table 1:** Membrane Diafiltration Purification Conditions Used during Chain Extension by OP-LPOS

membrane	pressure (bar)^a^	membrane channels^b^	conc. (PEG-oligo % w/v)^c^	cross-flow velocity (m/s)^d^	feed flow rate (mL/min)
NF 750 Da	10	19	0.9–0.5	0.2	70
UF 2000 Da	5	1	1–0.3	1	70

a 15 and 10 bar maximum pressures for NF and UF, respectively.

b Each tubular membrane contained a different number of channels; a 19-channel membrane was used for the tight NF membrane to allow for reasonable filtration cycle times at lab scale. The UF membrane has looser pores and therefore higher permeance, which allowed reasonable filtration times using the 1-channel membrane.

c NF filtration used a fixed system volume of 200 mL, whereas the UF synthesis used a concentration of 1% w/v relative to PEG-oligo used for 2–11mer (**3d**–**3m**) steps, then 0.5% w/v for 12–16mer (**3n**–**3r**) steps, and finally 17-18mer steps used 0.3% w/v (**3s**–**3t**).

d Inopor recommend 3–5 m/s for optimal cross-flow velocity to reduce concentration polarization.

**Figure 2:**
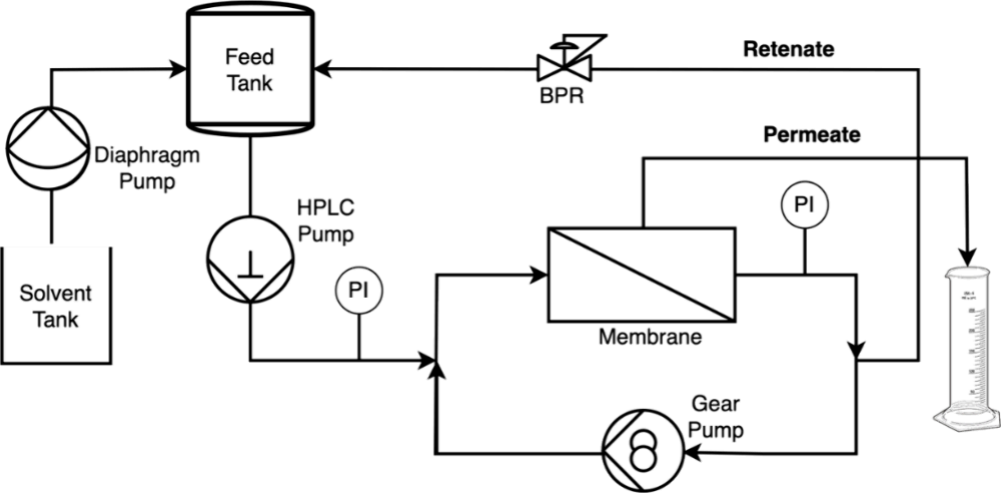
Cross-flow membrane filtration rig schematic.

### 2.3 Membrane Performance Characteristics

Rejection (*R*ej_j_) measures membrane selectivity and describes the percentage of solutes (j) which are unable to permeate the membrane, which is calculated by the equation below, where *C*_*p*,j_ is the concentration of the solute in the permeate and *C*_*r*,j_ is the concentration of the solute in the retentate.

$$Rej_{j}(\%)=\left(1-\frac{C_{p,j}}{C_{r,j}}\right)\times 100$$ 1

Membrane performance can also be characterized by permeance (*P*_m_), which is defined as the liquid volume flow through the membrane per unit area, per unit time, normalized to applied pressure (Lm^–2^ h^–1^ bar^–1^). It is a function of the volumetric flow rate (*Q*_p_) and membrane area (*A*_M_).

$$P_{m}=\frac{Q_{P}}{A_{M}\Delta P}$$ 2

Diavolumes are a time-like dimensionless variable commonly used to describe the filtration performance in CVD, where the system volume is kept constant by matching the permeate flow rate with fresh solvent addition. Diavolumes are calculated by the equation down below where *J* is flux (Lm^–2^ h^–1^), A is the membrane area (m^2^), *t* is filtration time (h), and *V* is the total system volume (L).

$$\mathrm{Diavolume}\;(\mathrm{DV})=\frac{J\times A\times t}{V}$$ 3

Yield of a given solute in the retentate during CVD can be calculated based on solute rejection (*R*ej_j_), which decreases with the number of diavolumes (DV) permeated.

$$\mathrm{Filtration}\;{\mathrm{yield}}_{j}\;(\%)=e^{\mathrm{DV}*(\frac{Rej_{j}}{100}-1)}\times 100$$ 4

### 2.4 Membrane Requirements and Screening

For successful membrane-assisted LPOS several criteria must be met: (i) rejection (>99%) of the growing PEG-oligo to ensure high yields, (ii) low rejection (ideally 0%) of excess reagents and byproducts to ensure high purity, (iii) sufficient membrane permeance for acceptable productivity, (iv) high chemical stability and durability over time, (v) minimal diavolume use for solvent efficiency, and (vi) high PEG-oligo solubility and control of membrane concentration polarization/fouling.

Organic solvent resistant (OSR) membrane use in the pharmaceutical industry is an emerging field, and therefore, there is little information on suitable membranes for early-stage process development. OSR membrane filtration depends on complex solute–solvent-membrane interactions, which limit predictability, requiring screening experiments for each new application.^32–35^ Several commercially available polymeric membranes performed well in screening experiments but were unsuitable when tested under real LPOS conditions (detailed discussion in the Supporting Information). Instead, more stable ceramic tubular membranes were purchased from Inopor for membrane screening: NF 750 Da MWCO (1 nm pore size) and UF 2000 Da MWCO (3 nm pore size).^36^ Both membranes were highly stable and showed >99% rejection of loaded 4-arm PEGs and >44% rejection of 5′-Dmtr-2′-OMe-G^ib^ phosphoramidite (869.95 g/mol), with a detailed discussion included in the Supporting Information.

### 2.5 General Overview of Chemistry and Chain Extension

One of the primary limitations in oligonucleotide synthesis is the incompatibility of reagents in each step, requiring repeated intermediate purification after each reaction step, leading to product losses and high solvent requirements.^8^ One-pot liquid-phase oligonucleotide synthesis (OP-LPOS) employs sequential coupling, sulfurization/oxidation, and detritylation, allowing one separation step per synthetic cycle to reduce unit operations and solvent consumption.^6–8,17,37^ OP-LPOS has not been applied in membrane-assisted LPOS to date, and we predicted that membrane separation could be enhanced through the generation of smaller phosphoramidite byproducts by removing the bulky dimethoxytrityl (Dmtr, 303 Da) temporary protecting group before diafiltration. Reducing the number of separation steps is also beneficial, as each diafiltration is expected to incur yield loss if rejection of the product is not 100%, which is challenging to achieve.^38^

Figure 3 summarizes the overall process and chemistry used in this study to produce 2′-OMe phosphorothioate oligonucleotides using one-pot liquid-phase oligonucleotide synthesis (OP-LPOS) assisted by liquid-phase separation via organic solvent resistant (OSR) membrane nanofiltration (OSN) or ultrafiltration (OSU). The first nucleotide (2′-OMe uridine) is tethered to 4-arm PEGs by a succinate linker through either ester or amide coupling chemistries. After support loading, the growing PEG-oligonucleotide is cycled through multiple rounds of chain extension (coupling, desulfurization, and detritylation) by OP-LPOS, followed by intermediate purification by CVD, using ceramic OSU/OSN membranes. After repeating the cycle multiple times, the oligo is fully deprotected and cleaved from the PEG support by ammonolysis. The crude product purity is determined by IP-RP-HPLC, and the sequence is identified by MALDI-TOF.

**Figure 3:**
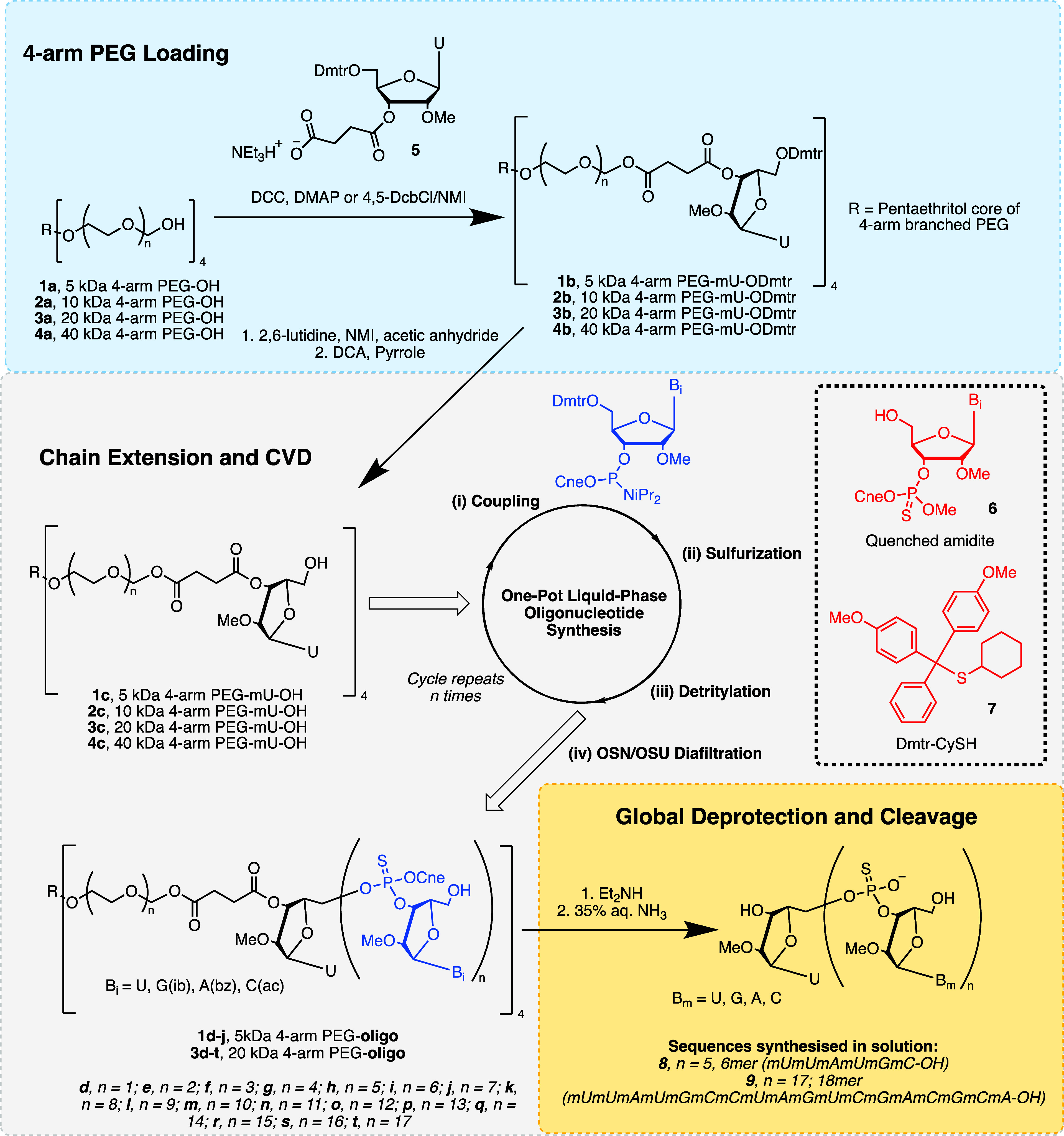
Schematic representation of the overall process developed. One-pot LPOS chain extension conditions per arm: (i) Coupling: 2’-OMe phosphoramidite (1.5 equiv), DCI (4 equiv), 10% w/v 3 Å molecular sieves, MeCN, RT, 30–60 min; amidite quench: MeOH (5 equiv); (ii) sulfurization: PADS (9 equiv) or POS (3 equiv), MeCN, RT, 60 min; (iii) detritylation: TFA (15–180 equiv) and CySH (5–15 equiv), MeCN:DCM (2:1 v/v), 0 °C, 30–130 min; acid quench: pyridine (20–185 equiv), MeCN:DCM (2:1 v/v), 0 °C, 10 min; (iv) diafiltration purification using ceramic OSN/OSU membrane. Cycle is repeated *n* times until desired sequence is reached. [Me: methyl, Dmtr: dimethoxytrityl, ib: isobutyryl, bz: benzoyl, ac: acetyl, Cne: cyanoethyl, iPr: isopropyl].

#### 2.5.1 4-Arm PEG Loading

Polydisperse 4-arm branched polyethylene glycol (PEG) was selected as the soluble support due to its high solubility in organic solvents, availability in various molecular weights, and affordability. Its branched nature facilitates high retention by membrane filtration, as it has been shown that linear PEG can seep through tight pores even at very large sizes.^18^ Previous studies have shown that oligonucleotide solubility can be challenging at longer chain lengths in LPOS using low molecular weight PEGs and other soluble supports.^8,9,12,22^ We hypothesized that using PEGs between 5 and 40 kDa would provide sufficient solubility for full-length products (∼18–25mer) while ensuring high membrane rejection.

4-arm PEGs (**1a**–**4a**) were purchased with terminal hydroxy groups in various molecular weights (Mn_MALDI/GPC_ = 5108 g/mol, 10 707 g/mol, 20 969 g/mol and 41 170 g/mol: polydispersity index (PDI) = 1.02–1.04, HPLC purity 97.4–99.6%). The supports were loaded with the first nucleoside, 5′-O–Dmtr-3′-O-succinyl-2′-O-methyluridine **5,** using either DCC/DMAP or DcbCl/NMI, which have been used in previous LPOS applications.^10,22^ DCC/DMAP was preferred as the DcbCl coupling reagent formed a Dcb-ester impurity that could not be removed by chromatography in our hands. Loading (%) efficiency of compounds **1b**–**4b** was determined by ^1^H NMR integration of PEG compared to Dmtr*H* or UC*H* peaks and MALDI-TOF of PEG before and after loading.^13,39^ Unloaded hydroxy groups were then capped by acetic anhydride, and Dmtr was removed using DCA and pyrrole to give loaded supports **1c**–**4c**. Detailed experimental data of support loading, including NMR and MALDI-TOF spectra, can be found in the Supporting Information.

#### 2.5.2 General Procedure for LPOS

Detailed dimer synthesis and membrane purification from **3c** to **3d** are described using a UF 2000 Da membrane. An equivalent procedure is used for all chain extensions and membrane purification steps. Each reaction used a slightly different PEG-oligo concentration during couplings (varying between 1.8 and 22.3% w/v), depending on oligonucleotide solubility as chain length increased, requiring more acetonitrile to solubilize (solubility during coupling for 18mer **3t** shown in Figure 7).

##### 2.5.2.1 Synthesis Cycle (OP-LPOS)

The starting 4-arm 20 kDa PEG-mU–OH **3c** (3.0 g, 0.134 mmol) was coevaporated with anhydrous acetonitrile (3 × 12 mL). 5′-O–Dmtr-2′-OMe-C^ac^ phosphoramidite (1.5 equiv per 5′–OH, 0.611 g, 0.804 mmol) was coevaporated with anhydrous acetonitrile (3 × 4 mL), dissolved in dry acetonitrile (15 mL), and added to PEG-mU–OH. 3 Å molecular sieves (10% w/v) were added and left stirring for 10 min to remove any residual water. 4,5-Dicyanoimidazole (DCI) (4 eq per 5′–OH, 0.253 g, 2.143 mmol) was added and left stirring for 60 min. HPLC confirmed reaction completion. Anhydrous MeOH (5 eq per 5′–OH, 0.086 g, 2.679 mmol) was added and left stirring for 20 min to quench remaining amidites. 3-phenyl 1,2,4-dithiazoline-5-one (POS) (3 eq per 5′-ODmtr, 0.314 g, 1.607 mmol) was added as a solid and left stirring for 60 min to sulfurize. Anhydrous DCM (7.5 mL) was added to give final solvent mix of MeCN:DCM (2:1). Cyclohexanethiol (CySH) (5 eq per 5′-ODmtr, 0.311 g, 2.679 mmol) and trifluoroacetic acid (TFA) (15 eq per 5′-ODmtr, 0.90 g, 8.036 mmol) were added to reaction mixture dropwise at 0 °C and left stirring for 60 min to remove 5′-ODmtr protecting group. HPLC confirmed reaction completion, and pyridine (20 eq per 5′–OH, 0.847 g, 10.715 mmol) was added dropwise at 0 °C and left stirring for 10 min to quench acid before filtration.

##### 2.5.2.2 Synthesis Cycle (CVD)

The reaction mixtures were poured into the feed tank, using a filter paper to remove the molecular sieves, and diluted to a final system volume with neat acetonitrile diafiltration solvent. The system was pressurized to 5 bar for UF 2000 Da membrane and left recycling for 60 min to reach steady state. Fresh solvent was added during diafiltration to match the rate of permeance, which averaged 20.2 Lm^–2^ h^–1^ bar^–1^. Filtration was stopped after 5 diavolumes permeated, giving a final PEG-dimer purity of 98.0% and final yield of 99.6% by HPLC. The purified product was washed out of the rig with acetonitrile, and the solvent was removed under reduced pressure to give 4-arm PEG-dimer product **3d** (3.03 g, 94.3% recovered mass yield) as a glassy light brown solid. ^31^P NMR (162 MHz, CDCl_3_) δ = 67.03 (0.36P), 66.68 (0.64P). By using this synthesis cycle and repeating it (*n* = 5 and *n* = 17) times desired sequences 6mer **1j** and 18mer **3t** were obtained in 98% and 94% filtration yield,s respectively, calculated based upon rejection data (eq 4).

##### 2.5.2.3 General Procedure for Global Cleavage and Deprotection

PEG-oligo products were cleaved from PEG, and protecting groups were removed in a two-step deprotection. PEG-oligos (**1j** (66 mg), **3n** (72 mg)) were dissolved in MeCN (4 mL) and diethylamine (1 mL, 20% v/v) and left stirring at room temperature for 20 min to remove temporary cyanoethyl protecting groups on the backbone. The solvent was removed under reduced pressure, and products were dissolved in 30% aqueous NH_3_ (4 mL) and filtered through a pad of cotton wool to remove undissolved solids. Reactions were sealed and refluxed for 6 h at 45 °C to cleave from PEG and remove nucleobase protecting groups. The solutions were coevaporated with EtOH (3 × 10 mL) to remove any additional ammonia. Products were obtained by trituration with acetonitrile to remove PEG and collected as a pellet after centrifugation to give fully deprotected off-white powders after drying under vacuum (**8** (29 mg, 87% yield), **9** (27 mg, 79% yield)). Products identified by MALDI-TOF: **8** *m*/*z* calc. for [C_62_H_83_N_19_O_37_P_5_S_5_]^+^ [M + H]^+^: 2000.3; found 2000.6 and **9** *m*/*z* calc. for [C_188_H_247_N_65_O_108_P_17_S_17_]^−^[M-H]^−^: 6216.7; found 6217.0. IP-RP-HPLC purity was 81% for **8** and 72% for **9**.

## 3 Results and Discussion

### 3.1 Loading of First Nucleoside to Polydisperse Branched PEG

Initially, loading onto 4-arm PEG–OH was achieved using 2,6-dichlorobenzoyl chloride (DcbCl) as a coupling reagent.^22,40,41^ This loading method produced a Dcb-ester impurity on PEG, which does not affect oligonucleotide synthesis but complicates analysis by NMR during LPOS chain extension (NMR spectra in Supporting Information). Attempts to separate this impurity chromatographically were unsuccessful. Alternatively, loading onto 4-arm PEG–OH was achieved by Steglich esterification with DCC/DMAP, a method previously used to load linear PEG with a nucleotide by Bonora et al.^10,42^ The loading values, determined by NMR and MALDI-TOF, were 478, 283, 158, and 75 μmol oligonucleotide/g PEG support for supports **1b**, **2b**, **3b** and **4b**, respectively (further details in Supporting Information). Unloaded hydroxy groups were capped and detritylated to give supports **1c**–**4c** for commercial membrane screening.

### 3.2 Chain Extension by One-Pot Liquid-Phase Oligonucleotide Synthesis (OP-LPOS)

In this study, we adopted a one-pot synthesis strategy for membrane filtration and co-optimized with the Inopor ceramic NF and UF membranes to synthesize 6mer (**8**) and 18mer (**9**) 2′-OMe oligonucleotides. Homogeneous reaction conditions facilitated the use of in-line reaction and diafiltration monitoring by HPLC (Figure 4) and ^31^P NMR (Figure 5).^6,8^ The main findings and best conditions during the development of a membrane-compatible one-pot synthesis are outlined in this section.

**Figure 4:**
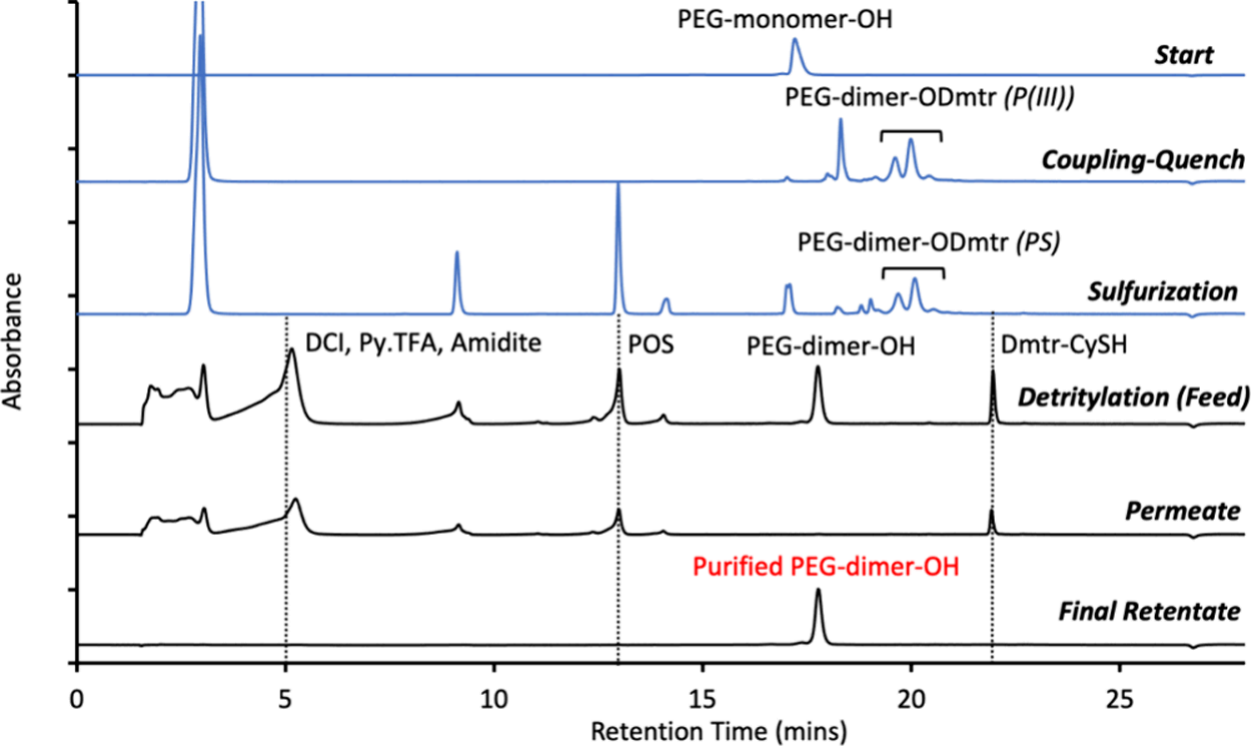
HPLC reaction and diafiltration monitoring using an OP-LPOS and UF 2000 Da membrane from 20 kDa 4-arm monomer PEG-mU–OH **3c** to purified dimer PEG-mUmU–OH **3d**. (Blue) HPLC trace for the starting detritylated PEG-bound uridine. Post coupling-sulfurization reaction; phosphoramidite related species are visible between 16 and 19 min, POS eluted at 13.5 min, DCI eluted at 2 min and the PEG-dimer-ODmtr peaks came out at around 20 min.(Blue) After detritylation and acid quench (feed); a peak for Dmtr-scavenger is visible at 25 min with detritylated amidites species eluting between 0 and 15 min, and the detritylated PEG-dimer–OH peak appearing at 18 min. Permeate and retentate HPLC traces are also shown where final PEG-dimer–OH **3c** showed 99.5% rejection by membrane and 98.0% Area purity by HPLC.

**Figure 5:**
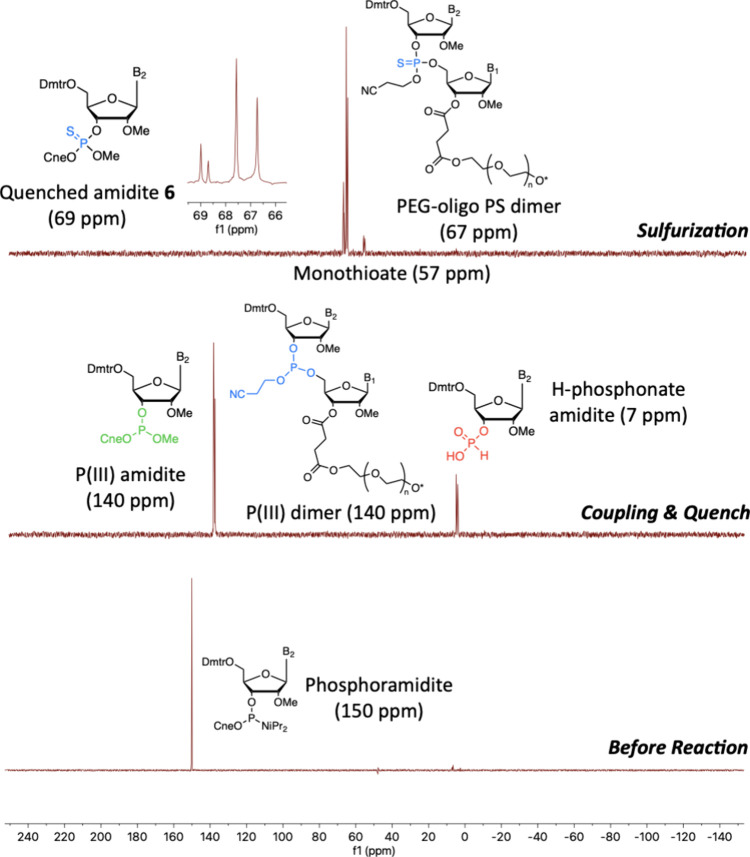
^31^P spectra (bottom) before coupling showing phosphoramidite before coupling and (middle) phosphite triester (PIII) peaks for amidite and PEG-dimer at 140 ppm and H-phosphonate byproduct for NMR preparation at 7 ppm (top) after sulfurization by PADS reagent (9 equiv per 5′-ODmtr) showing amidite and PEG-dimer phosphorothioate peaks at 67 ppm and minor monothioate peak at 57 ppm. Equivalent spectra obtained using POS reagent except for larger amount of monothioate after sulfurization.

#### 3.2.1 Coupling

Coupling of a nucleoside phosphoramidite to the support-bound nucleotide 5′–OH group requires the use of an efficient activator to form a phosphite triester (P^III^).^43,44^ In our procedure, PEG-oligo–OH was coevaporated with anhydrous acetonitrile (dissolved in solvent and then concentrated), then dissolved in anhydrous acetonitrile, and 10% w/v 3Å molecular sieves were added to remove residual water. Four equivalents of activator per 5′–OH were used to achieve high coupling efficiency with just 1.5 equiv of phosphoramidite per 5′–OH (typically 1.05–3 eq for LPOS and 2–3 eq for large-scale SPOS).^2,6^ The reactions were complete in less than 30 min by ^31^P NMR (Figure 5) and by HPLC (Figure 4). To minimize overcoupling addition impurities (*n* + 1), an additional phosphoramidite quenching step was included in the protocol with an excess of methanol (5 eq per 5′–OH).^6,24,25^ This quenching step formed quenched amidite **6**, as observed by ^31^P NMR (Figure 5).

#### 3.2.2 Sulfurization

The phosphite-triester P­(III) is unstable to acid and must be converted to a stable P­(V) species by sulfurization (or oxidation) prior to detritylation. In our adapted process, sulfurization was achieved using either phenylacetyl disulfide (PADS) (9 eq per 5′-ODmtr) or 3-phenyl 1,2,4-dithiazoline-5-one (POS) (3 eq per 5′-ODmtr), due to their high solubility in neat acetonitrile where POS is soluble up to 0.15 M and PADS is soluble up to 0.03 M.^45,46^ Complete sulfurization of phosphite triester (P­(III)) was typically achieved in under 30 min using both reagents, as monitored by ^31^P NMR (Figure 5). Other reagents, such as 3-((dimethylamino-methylidene)­amino)-3H-1,2,4-dithiazole-3-thione (DDTT) and xanthane hydride (XH), were poorly soluble in neat acetonitrile (0.02 and 0.005 M, respectively), leading to precipitation on the membrane surface, so they were not explored further. To increase solubility, PADS, DDTT, and XH are usually mixed with a base; however, in a one-pot process, the use of a base is undesirable as it would neutralize the acid required for the subsequent detritylation step. Usually, PADS is ‘aged’ in a solution of acetonitrile and 3-picoline to improve efficiency, which results in the formation of polysulfides over a number of days, giving rise to a black precipitate.^47^ Therefore, PADS was used without aging to avoid solids that could clog pumps or membrane pores, and a large excess (9 equiv per 5′-ODmtr) was applied to overcome the reduced efficiency. PADS was applied to 6mer (**8**) synthesis with NF 750 Da membrane, while POS was used for the 18mer (**9**) synthesis with 2000 Da membrane, and is the preferred sulfurization reagent due to superior solubility, efficiency, and higher purity final product (Figure 8).

When methanol quenching was included after coupling, only phosphorothioate peaks around 67 ppm and minor monothioate peaks around 57 ppm were observed after sulfurization (Figure 5). Excluding the quench led to the formation of numerous more impurities, observed by ^31^P NMR (Supporting Information).^12^ Simplifying the crude reaction mixture was a key innovation of this adapted one-pot route, where the large number of phosphoramidite byproducts has been challenging to remove in LPOS-OSN previously.^12^ A H-phosphonate product was also observed after coupling, arising due to hydrolysis of the phosphoramidite in the presence of DCI and wet CDCl_3_ while preparing NMR samples, but was not observed in any samples after sulfurization (Figure 5).^11,48,49^

#### 3.2.3 Detritylation

During detritylation, the dimethoxytrityl (Dmtr) protecting group is removed from the 5′–OH to allow for the addition of the next phosphoramidite in the sequence. In this study, TFA (15–180 equiv per 5′-ODmtr) with thiol-based cation scavenger cyclohexanethiol (CySH) (5–15 equiv per 5′-ODmtr) was effective for quantitative detritylation telescoped directly from the preceding reactions in each cycle. In LPOS the detritylation reaction is reversible, requiring a cation scavenger to prevent reattachment of the Dmtr group.^6,8,10,12,50–52^ Detritylation was slower in neat acetonitrile during dimer synthesis, so a mixed solvent system of MeCN:DCM (2:1) was used to improve efficiency (Supporting Information).^53^ In our hands, detritylation was usually complete by HPLC within 30–130 min at 0 °C (Figure 4). Detritylation was cooled to 0 °C as it has been proposed that cooling can increase efficiency, suppress depurination, and slow the deamination of cytosine nucleosides when residual water is present.^6,8^ The reaction gradually became slower as chain length increased, requiring more equivalents of acid (30–180 equiv) and longer reaction times (60–130 min) for completion. After detritylation was completed, the acid was quenched with an excess of pyridine before diafiltration to prevent extended exposure of the oligonucleotide sequence to acidic conditions.

### 3.3 OP-LPOS Synthesis and OSR Membrane Purification

To evaluate the performance of one-pot liquid-phase oligonucleotide synthesis (OP-LPOS) with membrane filtration a 6mer (**8**) was synthesized using the NF 750 Da membrane on a 4-arm 5 kDa PEG support **1c**, and an 18mer (**9**) was synthesized on a 4-arm 20 kDa PEG support **3c** using the UF 2000 Da membrane (compounds abbreviated as shown in Figure 3) using synthesis conditions outlined in Table 2. The phosphorothioate target sequence (UUAUGCCUAGUCGACGCA) was selected as it contained four RNA 2′-OMe nucleobases (UGAC), using standard protecting groups for cytosine (acetyl), guanine (isobutyryl), and adenine (benzoyl), to show the method is generally applicable to a range of nucleobases and modifications/protecting groups. For comparison, an additional synthesis was attempted using a literature two-pot LPOS method with the NF 750 Da membrane and a 4-arm 5 kDa PEG **1c** support (Supporting Information).

**Table 2:** Protocol for Chain Extension by One-Pot LPOS Integrated with the OSR Membrane Filtration

chain extension conditions^a^			
step/reagent	conditions	solvent	reaction time^b^
coupling	PEG-oligo (2–22% w/v)^c^		
	phosphoramidite8 (1.5 equiv per 5′–OH: mU, mC^ac^, mA^bz^, mG^ib^)	MeCN	30–60 min
	DCI (4 eq per 5′–OH)		
amidite quench	MeOH (5 eq per 5′–OH)	MeCN	10–20 min
sulfurization	PADS or POS (9 or 3 eq per 5′-ODmtr)^d^	MeCN	60 min
detritylation	TFA (15–180 eq per 5′-ODmtr) and CySH (5–15 eq per 5′-ODmtr)^e^	MeCN:DCM (2:1)	30–130 min
quench	Py (20–185 eq per 5′–OH)	MeCN:DCM (2:1)	10 min
molecular sieves	10% w/v 3 Å molecular sieves	-	-

a Coupling, amidite quench and sulfurization at RT, detritylation and acid quench steps at 0 °C.

b Long reactions times used to verify reaction completion by offline HPLC. Reaction yield was estimated based on LC or NMR relative area %.

c Dissolved PEG-oligo in minimum volume of acetonitrile to obtain final concentration before coupling. 6mer synthesis used 11–16% w/v. 18mer synthesis used 2–22% w/v.

d PADS used in 6mer synthesis with NF membrane and POS used in 18mer synthesis with UF membrane.

e Equivalents of TFA varied depending on detritylation progress by HPLC, more equivalents and extended reaction times were used if the reaction was incomplete after 60 min.

#### 3.3.1 OP-LPOS 6mer Synthesis with OSN Membrane Filtration

A two-pot synthesis was initially conducted using 4-arm 5 kDa PEG **1c** with NF 750 Da membrane to compare to a literature LPOS process.^8,12^ One chain extension involved coupling-sulfurization followed by diafiltration to purify, then detritylation, and a second round of diafiltration. The NF 750 Da membrane could remove all reagents and byproducts during LPOS in 14 diavolumes measured by HPLC and ^31^P NMR, and the final deprotected dimer filtration yield was 81%, with a purity of 97.5% after 14 diavolumes (details in Supporting Information).

After OP-LPOS, the reaction mixture was poured into the separation rig for purification, where PEG-oligo product rejection after the dimer (**1d**) stage was almost quantitative at 99.8%, rising to 100% during subsequent chain extensions. All reagents and byproducts were effectively removed in 10 diavolumes during dimer (**1d**) synthesis, as measured by HPLC and ^31^P NMR after dimer synthesis. It was not possible to estimate phosphoramidite byproduct rejection by HPLC due to overlapping peaks with pyridine and DCI, which also eluted around 5 min (Figure 4). Resolution of these peaks for an in-process check was not essential, as the impurity with by far the highest rejection at the dimer (**1d**) stage and throughout was the scavenged trityl protecting group, Dmtr-CySH **7**, at 35%. This is an unreactive impurity, which was specified for removal to a high product PEG-oligo purity of >97% by HPLC relative area% in each diafiltration, setting the required number of diavolumes between 8 and 10. All other reagents and impurities were poorly rejected and had been purged from the system to below detectable levels by HPLC. Over the course of the 6mer (**1j**) synthesis, the average PEG-oligo purity in the final retentate was 98% based on HPLC relative area %.

During iterative synthesis, the PEG-oligo was dissolved in the minimum volume of acetonitrile that provided a homogeneous solution before coupling to estimate the solubility at each chain extension. At the dimer stage (**1d**), PEG-oligo showed high solubility in acetonitrile (>160 mg/mL), which decreased gradually up to the 6mer (**1j**) stage (>100 mg/mL). The reactions proceeded to completion during all chain extension steps, as measured by HPLC. The PEG-oligo species remained highly soluble (>100 mg/mL) during the reaction steps, despite the decreasing solubility after each chain extension. However, problems were encountered at the membrane interface, where the permeance decreased from 2.2 to 1.1 L m^–2^ h^–1^ bar^–1^ and the rejection of Dmtr-CySH **15** increased from 35% to 76% between the dimer (**1d**) and 6mer (**1j**) stages. This was despite the bulk operating concentration of 0.5% w/v being much lower than the solubility of the solution during coupling in neat acetonitrile (11% w/v). Furthermore, the change in rejection factor was indicative of a significant change in concentration, thickness, or structure of the polarization layer. A sudden drop in filtration yield was observed at the 6mer (**1j**) stage, due to product precipitation on the membrane surface. To increase the solubility of the PEG-oligo, the diafiltration solvent was swapped to MeCN:MeOH (9:1 v/v) after 8 diavolumes, where permeance increased from 1.1 to 3.8 Lm^–2^ h^–1^ bar^–1^ and the Dmtr-CySH **7** rejection lowered to 26%, completing the filtration in 10 diavolumes (Figure 6). At this point, the PEG-oligo concentration in the retentate also increased, indicating that the PEG-oligo, which precipitated, was resolubilised to a large extent. The addition of methanol to the diafiltration solvent has been proposed to increase the solubility of PEG-oligo by disrupting the hydrogen bonding of nucleobases.^12^

**Figure 6:**
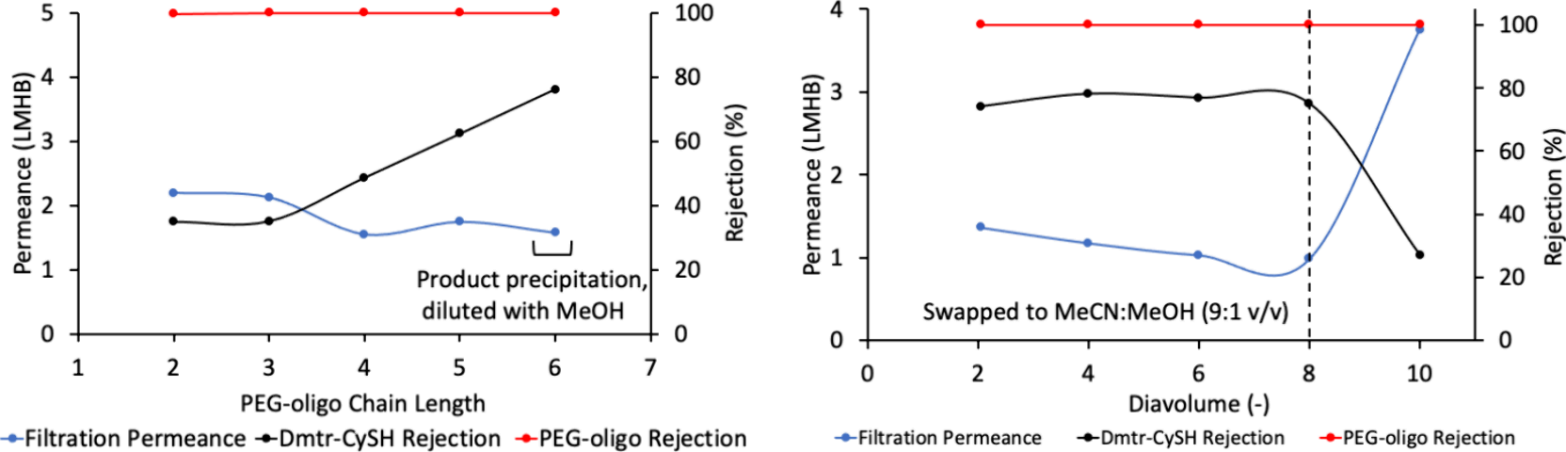
6mer (**8**) synthesis average permeance and Dmtr-CySH impurity **7** rejection trends as the chain length increased. Dashed line indicates when the solvent was swapped from neat acetonitrile to a mixed solvent system with methanol. (Right) 6mer (**1j**) chain extension step filtration permeance and Dmtr-CySH **7** rejection during diafiltration showing the impact of mixed acetonitrile/methanol diafiltration solvent.

Given the rapid decrease in product solubility with increasing chain length, it was predicted that the 4-arm 5 kDa PEG support (**1c**) was insufficient to solubilize full-length oligos under the current filtration conditions. It should be noted that the PEG-oligo solubility in acetonitrile during the reaction was still high (>100 mg/mL) by the 6mer (**1j**) stage, indicating that PEG-oligo solubility was primarily a membrane interface issue. Synthesis was terminated at this point, and the 6mer (**8**) product was obtained after global deprotection. The purity (81%) was analyzed by IP-RP-HPLC, and the sequence was identified by MALDI-TOF (Figure 8).

#### 3.3.2 OP-LPOS 18mer Synthesis with OSU Membrane Filtration

An 18mer (**9**) 2′-OMe PS oligonucleotide was synthesized using a 4-arm 20 kDa PEG support **3c** and UF 2000 Da membrane. A 20 kDa PEG support was selected in an attempt to provide a balance between sufficient solubilizing power, high membrane rejection, and reasonable oligonucleotide loading efficiency, as this method aims to be generally applicable, rather than sitting at a global optimum for a given oligo length or sequence.

After OP-LPOS, the reaction mixture was poured into the separation rig for purification, where PEG-oligo product rejection at the dimer (**3d**) stage was 99.5%, which increased to 100% by the 6mer (**3h**) stage. All reagents and byproducts showed low membrane rejection and were removed in just 5 diavolumes per step, as confirmed by HPLC and ^31^P NMR (Figure 4), compared to 8–10 diavolumes in the one-pot 6mer (**8**) synthesis or 14 diavolumes using the previously reported two-pot approach (Supporting Information), both employing the NF 750 Da membrane. This diavolume efficiency is much lower than Kim et al., who used 27 diavolumes per chain extension cycle employing a PBI OSN polymeric membrane in their LPOS approach.^12,15^ The average PEG-oligo purity in the retentate after each cycle was 97%, with Dmtr-CySH **7** as the only remaining impurity.

During the dimer (**3d**) coupling steps, the PEG-oligo exhibited high solubility in acetonitrile (>210 mg/mL), which gradually decreased until the 18mer (**3t**) stage (>18 mg/mL) (Figure 7). The reactions proceeded to completion for all reaction steps by HPLC, despite decreasing PEG-oligo solubility. However, the determination of coupling reaction completion became more challenging as chain length increased, and the 4-arm PEG-oligo conjugate product peak by HPLC became broader (Supporting Information). Similarly to the previous 6mer (**8**) NF synthesis run, problems were encountered at the membrane interface in the UF-enabled synthesis run, where the permeance decreased with increasing chain length, from 20.2 Lm^–2^ h^–1^ bar^–1^ at the dimer (**3d**) stage to 7.3 Lm^–2^ h^–1^ bar^–1^ by the 10mer (**3l**). Byproduct Dmtr-CySH **7** rejection also increased from −16.8% to a maximum of 93.5% by 10mer (Figure 7), making removal impractical with neat acetonitrile diafiltration. At this point, diafiltration solvent was swapped to MeCN:MeOH (9:1 v/v), which reduced Dmtr-CySH **7** rejection to 76% and increased permeance to 13.6 Lm^2–^h^–1^bar^–1^ from 7.3 Lm^–2^ h^–1^ bar^–1^, completing the filtration in 6 diavolumes, to a purity of 91.3%, without effecting PEG-oligo rejection, which remained at 100%.

**Figure 7:**
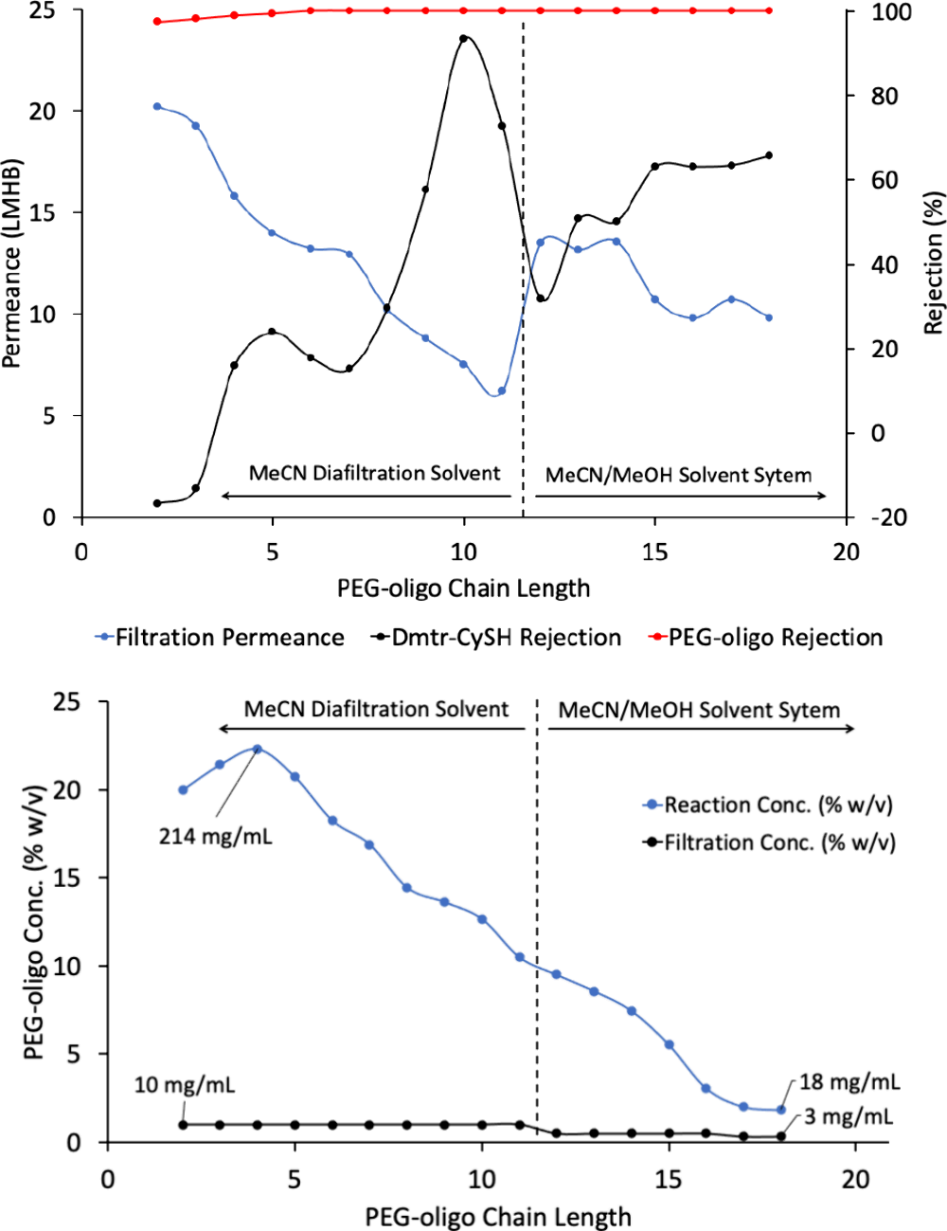
(Top) 18mer synthesis permeance and Dmtr-CySH impurity rejection trends as the chain length increased. Membrane was replaced before 11mer filtration. Neat MeCN solvent and 1% w/v PEG oligo filtration conc. were used in 2–9mer and 11mer filtration steps. Mixed MeCN:MeOH (9:1 or 4:1 v/v) diafiltration and 0.3–0.5% w/v PEG oligo filtration conc. were used in 10mer and 12–18mer steps (Bottom) PEG-oligo concentration (% w/v) in acetonitrile during coupling reaction (for instance monomer to dimer coupling reaction started with 20% w/v of monomer) and during diafiltration.

The pure acetonitrile flux through the membrane decreased from 25 to 20 L m^–2^ h^–1^ bar^–1^ between the dimer (**3d**) and 10mer (**3l**) steps, possibly due to membrane fouling, leading to a membrane replacement for improved filtration times.^54^ During the next chain extension to the 11mer (**3m**), poor filtration performance occurred despite the use of a new membrane, indicated by low permeance at 6.2 Lm^–2^ h^–1^ bar^–1^ and high Dmtr-CySH **7** rejection of 73%. The 11mer (**3m**) filtration was completed using neat acetonitrile diafiltration solvent, with some Dmtr-CySH **7** remaining in the final retentate (91.4% purity) after 5 diavolumes. For the 12mer (**3n**) step, the filtration concentration was halved to 0.5% w/v to improve the filtration performance. However, the permeance remained low at 5.9 Lm^–2^ h^–1^ bar^–1^, meaning ∼14 h of diafiltration would be required to permeate 5 diavolumes. Diafiltration solvent was swapped to MeCN:MeOH (9:1 v/v) to increase permeance to 13.2 Lm^–2^ h^–1^ bar^–1^, allowing filtration to be completed in 6 h. From the 13mer stage (**3o**) to 16mer (**3r**), the filtration concentration was kept at 0.5% w/v, and MeCN:MeOH (4:1 v/v) was used as the diafiltration solvent. During these steps, the permeance decreased from 13.2 to 10.7 Lm^–2^ h^–1^ bar^–1^, and Dmtr-CySH **7** rejection remained between 50.7 and 63.1%. Extension to 17mer (**3s**) and 18mer (**3t**) stages required a further decrease in the filtration concentration to 0.3% w/v, using the same diafiltration solvent (MeCN:MeOH (4:1 v/v). At this point, product precipitation was observed for the HPLC pump filter and in-line filter, requiring filter cleaning by sonication after 17mer (**3s**) filtration was complete. Despite this, the permeance was similar to previous steps between 9.8 to 10.7 Lm^–2^ h^–1^ bar^–1^ and Dmtr-CySH **7** rejection remained at 63.5–65.7%.

No product precipitation was observed during these experiments until the 17mer (**3s**), likely due to the larger 20 kDa PEG size and increased cross-flow velocity to 1 m/s, compared to the 6mer (**8**) synthesis using a 4-arm 5 kDa support and NF 750 Da membrane. The 18mer (**9**) product was obtained after global deprotection, and purity (72%) was analyzed by IP-RP-HPLC, and the sequence was identified by MALDI-TOF (Figure 8).

**Figure 8:**
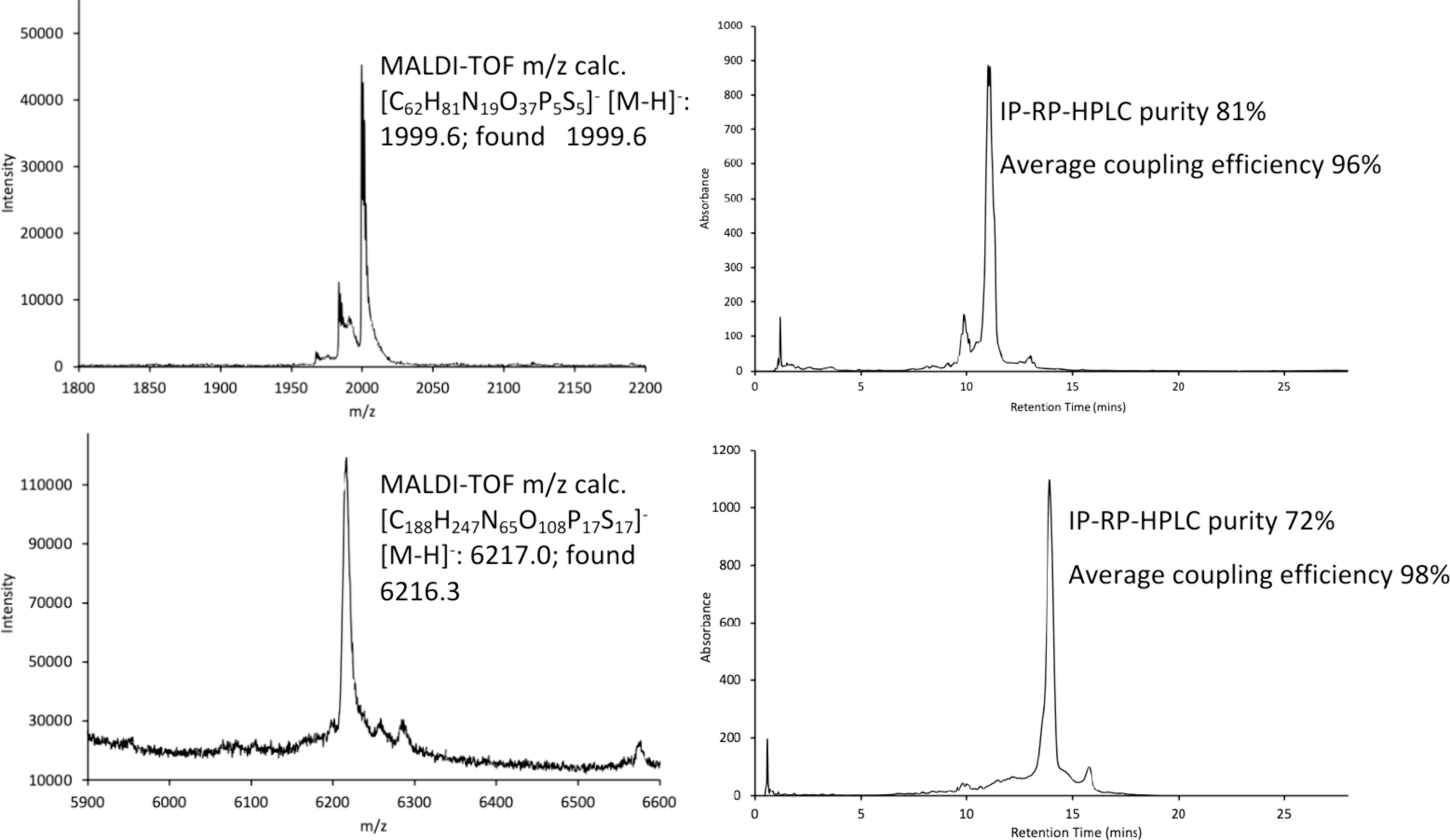
(Top) MALDI-TOF mass spectrum and IP-RP-HPLC chromatogram of 6mer **8** (UUAUGC) synthesized with NF 750 Da support (Bottom) MALDI-TOF mass spectrum and IP-RP-HPLC chromatogram of 18mer **9** (UUAUGCCUAGUCGACGCA) synthesized with UF 2000 Da support. MALDI-TOF spectra were obtained in linear negative mode. IR-RP-HPLC conditions: column Waters XBridge C18 (5 μm, 2.1 mm × 100 mm); gradient: A: 1 M TEAA in DI water B: MeCN (3 to 50%, 30 min); flow rate = 0.3 mL/min; λ = 260 nm; *T* = 50 °C.

### 3.4 Yield of Oligos Synthesized by OP-LPOS

The 6mer (**8**) and 18mer (**9**) synthesis was conducted using an initial mass of 1.6 and 3.0 g, respectively, with a summary of the yields and rejections during each synthesis included in Table 3. Yields in this study are based on rejection values calculated from the concentration of PEG-oligo in the retentate and permeate (eq 4) with stepwise isolated mass yields contained in the Supporting Information. Overall, the yields for each chain extension were nearly quantitative for 6mer and 18mer syntheses due to high PEG-oligo rejection values. PEG-oligo rejection rose from 99.8 to 100% by the 3mer (**1e**) stage during 6mer (**8**) synthesis with the NF 750 Da membrane and 5 kDa PEG support. Whereas, using the wider UF 2000 Da membrane and 20 kDa PEG support, the initial PEG-oligo rejection was lower at 99.5%, rising to 100% by the 6mer (**3h**) stage.

**Table 3:** Summary of 6mer and 18mer Syntheses with NF 750 and UF 2000 Da Ceramic Membranes

membrane	Inopor NF 750 Da	Inopor UF 2000 Da
2′-OMe PS oligo sequence	6mer (**8**)	18mer (**9**)
4-arm PEG support	5 kDa **1c**	20 kDa **3c**
rejection (%)	99.8–100	99.5–100
final yield based on rejection (%)	98	94
diavolumes (per chain extension)	8–10	5
average PEG-oligo HPLC purity (%)^a^	98	97
average permeance (Lm^2–^ h^–1^ bar^–1^)	1.8	12.5
PEG-oligo concentration (% w/v)^b^	0.9–0.5	1–0.3

a The purity was determined based on the relative area by LC-DAD.

b NF filtration used a fixed system volume of 200 mL, whereas the UF synthesis used a concentration of 1% w/v relative to PEG-oligo used for 2–11mer (**3d**–**3m**) steps, then 0.5% w/v for 12–16mer (**3n**–**3r**) steps, and finally, 17–18mer steps used 0.3% w/v (**3s**–**3t**).

### 3.5 Analysis of Oligo Products after Cleavage and Deprotection

6mer (**8**) and 18mer (**9**) products were cleaved from PEG in a two-step protocol. First, cyanoethyl protecting groups were removed using diethylamine, followed by the removal of remaining base protecting groups and cleavage from PEG by heating in aqueous ammonia at 45 °C. Purity was determined by ion-paired reversed-phase HPLC (IP-RP-HPLC) by integrating the main peak to determine UV purity. Identity of product and impurities was confirmed using MALDI-TOF, analyzing in the linear negative mode (Figure 8). This “cleave-and-analyse” approach requires only a small amount of sample and can be used at any point during synthesis to monitor impurity generation by HPLC and MS, a major advantage of LPOS.

The 6mer (**8**) was synthesized with 81% purity, corresponding to a 96% coupling efficiency, whereas the 18mer (**9**) was synthesized with a purity of 72%, corresponding to a higher coupling efficiency of 98% (Figure 8) employing just 1.5 eq of phosphoramidite. The 18mer (**9**) purity was much higher and aligns well with crude 2′-OMe PS oligo purity synthesized by SPOS, which is around 71–84%, assuming 98–99% coupling efficiency, usually employing 2–3 equiv of phosphoramidites on a large scale.

The 6mer (**8**) purity was slightly lower than anticipated, likely due to product remaining on the NF membrane after washout or due to precipitated product in pump filters, leading to early eluting truncated “shortmer” (n-1) impurity identified by MALDI-TOF. Additionally, an early eluting phosphate (PO) impurity (−16 Da) was also observable for the 6mer (**8**), likely due to the use of PADS, which has reduced efficiency in neat acetonitrile without “aging” in a base.^55^ Related to this, if the phosphite triester (P­(III)) post-coupling is not sulfurized, it is unstable to acidic conditions during detritylation in the next setup, which may result in bond cleavage and therefore n-1 impurity generation. The higher coupling efficiency of the 18mer (**9**) was attributed to improved recovery from the membrane rig after purification due to higher cross-flow velocities and the superior efficiency of POS in neat acetonitrile compared to PADS. For the 18mer (**9**), an addition “longmer” (*n* + 1) impurity was observed at M+359 Da, which is characteristic for 2′-OMe-G, indicating that unwanted coupling occurred. This may be a result of the extended coupling times used to monitor coupling reaction by HPLC, where the acidic activator DCI could have led to premature detritylation, giving rise to unwanted coupling.^56^ We anticipate that these impurities can be minimized through further reaction and filtration optimization and by the addition of in-line analysis techniques (NMR, IR, LC–MS) for further process understanding.

### 3.6 Assessment and Future Optimization of Membrane Diafiltration

Membrane diafiltration proceeded well during 6mer (**8**) (4-arm 5 kDa PEG, NF 750 Da) and 18mer (**9**) (4-arm 20 kDa PEG, UF 2000 Da) syntheses where PEG-oligo rejection was very high at 99.8–100 and 99.5–100% enabling high product recovery after each chain extension cycle with stepwise purity >97% by HPLC %Area. In particular, the UF platform was most promising, achieving high levels of permanence (12.5 Lm^–2^ h^–1^ bar^–1^) while enabling efficient separation with just 5 diavolumes. The synthesis runs proceeded up to the 6mer (**1j**) and 10mer (**3l**) stages before diafiltration became challenging using neat acetonitrile as the diafiltration solvent. After these PEG-oligo chain lengths, lower permeance and increased impurity rejection (Dmtr-CySH **7**) were limiting.

Understanding the exact causes for decreased performance in OSN/OSU is not straightforward due to a complex interplay of solute, solvent, and membrane interactions.^26,35^ Given the relatively high PEG-oligo solubility during reactions, the issue stems from increased concentration polarization and/or interaction with the membrane at this interface. Concentration polarization occurs when feed components permeate the membrane at different rates, creating a concentration gradient near the membrane surface, which can lead to solute accumulation, reduced permeance, and increased impurity rejection.^28^ Gradual membrane fouling, which is more prevalent with higher levels of concentration polarization, was also suspected as pure solvent permeance decreased overtime. Fouling may occur due to highly polar solutes strongly interacting with the hydrophilic membrane, resulting in absorption of solutes inside membrane pores, or fouling may occur by simple solute precipitation on the membrane surface or inside the pores.^26,57^ Several strategies may be implemented, including but not limited to increasing the cross-flow velocities, improving the solubility of the growing PEG-oligos (PEG size, oligo sequence and modifications, solvent selection), and replacing Dmtr-CySH **7** byproduct.

The cross-flow velocities (0.2 and 1 m/s) employed during 6mer (**8**) and 18mer (**9**) synthesis runs are below the supplier’s recommended operating range (3–5 m/s) provides insufficient turbulence to prevent significant solute accumulation at the membrane surface at longer chain lengths.^28,57^ At larger scales, further optimization involving new pumps to provide much higher cross-flow velocities, along with optimized transmembrane pressure and feed concentration, is expected to improve filtration performance.^28,57,58^

During this study, the selection of an appropriately sized PEG was essential to ensure high PEG-oligo solubility in acetonitrile. We observed solubility enhancement using a 20 kDa PEG (>180 mg/mL) over a 5 kDa PEG (>100 mg/mL) for the same 6mer (**1j**/**3j**) sequence. The oligonucleotide sequence itself also has a large effect on the overall solubility when attached to 4-arm PEGs, with some sequences forming secondary structures that are challenging to dissolve, or the different solubilities of the phosphate and phosphorothioate backbones.^59–61^ Additionally, protecting uridine bases and using more lipophilic protecting groups for the other nucleobases can improve oligonucleotide solubility in LPOS/SPOS.^9,11,24,62^

Ideally neat acetonitrile or a single mixed solvent system should be used for reaction and filtration steps: however, DCM was employed with acetonitrile in the reaction to improve detritylation efficiency, and methanol was added to the acetonitrile diafiltration solvent to improve PEG-oligo solubility and therefore diafiltration performance at longer chain lengths (after the 6mer (**1j**) and 10mer (**3l**) lengths). Solubility may be enhanced by the addition of methanol to acetonitrile by disruption of H-bonding between nucleobases, but it may also disrupt solute adsorption in the concentration polarization layer at the membrane interface or inside the pores.^12^ At large scales, however, methanol must be removed or replaced from the diafiltration solvent to prevent phosphoramidite quenching in subsequent chain extensions. Using methanol was not a concern during the synthesis runs presented in this study due to product isolation and evaporation to dryness after each diafiltration, which is more challenging at a large scale.

For the acetonitrile/methanol system, a low-boiling azeotrope is found, which can be exploited in an azeotropic batch distillation solvent swap to remove methanol post-diafiltration. A Dynochem simulation was performed (discussed in detail in the Supporting Information), which shows that the distillation of methanol to very low levels is feasible, potentially enabling the use of a mixed acetonitrile/methanol diafiltration solvent system at large scale.

Methanol could also be replaced with a polar aprotic solvent, such as DMF and sulfolane. When mixed with acetonitrile, these solvents have been shown to improve solubility of challenging oligonucleotide sequences in SPOS and LPOS.^9,63,64^ These solvents also have the added benefit of being compatible with the coupling chemistry during chain extension. To test if these solvents are viable in our developed OP-LPOS protocol we performed an additional dimer syntheses and membrane screening experiment (Supporting Information) where acetonitrile:sulfolane (3:1 v/v) gave the best results as all reaction steps proceeded near-completion (>99%) and furthermore this mixed solvent system was compatible with the UF membrane filtration.

The only significant impurity remaining after each diafiltration was Dmtr-CySH **7**, where the rejection was low and unproblematic during early chain extension cycles but increased significantly when the membrane performance decreased in later cycles. Nevertheless, this is an improvement over Kim et al., who found that the equivalent Dmtr-pyrrole byproduct in their nanostar sieving LPOS-OSN approach showed >90% rejection, requiring an additional precipitation step.^12^ The low rejection during early cycles may be attributed to the solute’s hydrophobic nature, resulting in a low affinity for the hydrophilic membrane surface, while it is likely that the rejection increased due to concentration polarization, which could be reduced by increasing cross-flow velocity. Dmtr-CySH **7** is nonreactive, and small amounts of this impurity remaining after each chain extension did not greatly affect the syntheses. However, Dmtr-CySH **7** could pose a risk for membrane fouling due to its poor solubility in acetonitrile (>20 mg/mL), suggesting that a change in scavenger molecule or protecting group would be advantageous. For example, replacing CySH with a more polar scavenger could facilitate removal by diafiltration through hydrophilic ceramic membranes. Similarly, the Dmtr group may not be optimal for LPOS, particularly for membrane-based approaches, due to its large size and hydrophobic character. Dmtr protection poses other challenges, such as the reversibility of the reaction, potential depurination, and dependence on oligonucleotide length, sequence, and scale.^17^ Switching to a smaller, acid-labile and nonreversible group, such as the 2-isopropoxyprop-2-ylacetal (IIP) protecting group, may be beneficial if quantitative deblocking can be achieved.^65^

### 3.7 Sustainability Analysis

The development of a more sustainable manufacturing process for oligonucleotides requires an understanding of the material efficiency to generate the product.^3^ Process mass intensity (PMI) serves as a key metric, calculating the total amount of starting materials, reagents, and solvents to generate 1 kg of API. In SPOS, the PMI was estimated to be approximately 4300 or ∼200 kg/kg per nucleotide.^3^ To compare to other LPOS strategies, we evaluated two methods: the kilogram scale convergent LPOS precipitation approach by Zhou et al. at Biogen, which had a PMI ∼ 63 kg/kg, and the membrane-based nanostar sieving approach by Kim et al. from the Livingston group, which had a PMI ∼ 2480 kg/kg.^3,6,12,66^

For our developed OP-LPOS via UF membrane process, the PMI was estimated for producing 1 kg of a 20-mer PS 2′-OMe oligonucleotide based upon average values obtained experimentally (filtration yield of 99.6%, coupling efficiency of 98%, filtration and reaction conc. of 1% w/v and 12% w/v respectively, 5 diavolumes to purity and 2 diavolumes to collect) and input into the ACS Green Chemistry PMI calculator.^67^ Three further situations were also considered, including (i) implementation of a distillation process to recover 85% of diafiltration solvent, (ii) increased filtration concentration to 5% and integration into a single membrane reactor-separator unit, and (iii) a combination of these two scenarios.^23,68–70^

The PMI per nucleotide for our developed process was 718 kg/kg, which is significantly lower than the nanostar sieving approach but still higher than SPOS or Biogen’s. If our diafiltration was operated at a concentration of 1% w/v with 85% of the diafiltration solvent recovered by distillation, the PMI would be lowered to just 108 kg/kg. An 85% recovery is based on work by Novartis, which claims recovery of 85% of acetonitrile from SPOS is possible for certain steps.^68,69^ An average reaction and filtration concentration of 5% w/v provides a realistic future target for this OP-LPOS process, which yields a PMI of just 103 kg/kg that could be further reduced to just 16 kg/kg by implementing an additional solvent recovery step by distillation. At a large scale, merging reactions and filtrations into a single membrane reactor-separator unit would be beneficial to enable easier automation and reduce the number of transfer steps.^23,71^

These potential scenarios highlight the potential sustainability benefits of our developed process compared with current SPOS and LPOS methods (Figure 9). It should be noted that the scalability limitations of SPOS, issues with precipitation approaches, and challenges during nanostar sieving are not captured by the PMI metric. Additionally, the reduced equivalents of phosphoramidites in LPOS (1.05–2 equiv) compared to SPOS (2–3 eq or more for large-scale) are not captured by PMI, where these building blocks have a high associated PMI themselves.

**Figure 9:**
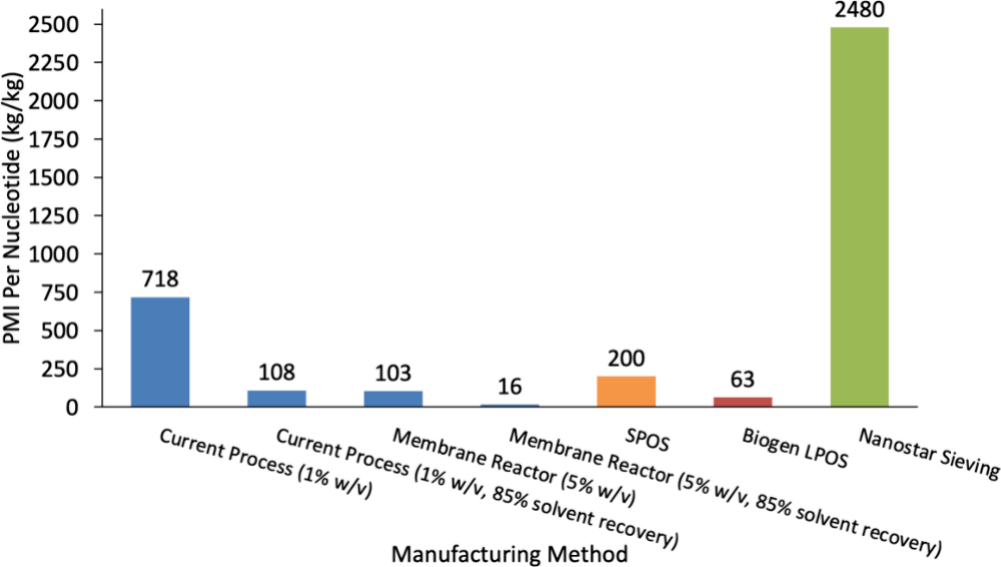
Comparison of PMI per nucleotide for the developed OP-LPOS process enhanced by OSU membrane filtration compared to the current LPOS and SPOS methods.

### 3.8 Advantages and Adaptation to Industrial Processing

The developed platform and process utilize affordable and commercially available reagents, membranes, and soluble supports, making it accessible for large-scale production of oligos using standard industrial batch reactors coupled with industrial membrane separation. A small excess of reagents was used to provide generally applicable reaction conditions using just 1.5 equiv of phosphoramidites per 5′–OH, with further optimization possible to further reduce the consumption of the most expensive reagent in the process. Coupling, sulfurization, and detritylation are telescoped into a one-pot reaction step, allowing just one membrane purification step for each chain extension cycle, significantly reducing solvent consumption and cycle time associated with multiple filtration steps.

The homogeneity of the reactions enables process monitoring capabilities. As demonstrated in this study, samples can be taken in situ at any time for HPLC or NMR analysis, providing the potential for robust process control and future Process Analytical Technology (PAT) integration. Additionally, small quantities of the product can be cleaved from the PEG support for more detailed analysis via HPLC and MALDI-TOF of the evolving oligomer product quality.

The ceramic membranes used in this platform exhibit excellent chemical stability under LPOS reaction conditions, achieving high product rejections (99.5–100%) and efficiently removing all reaction byproducts with as little as 5 diavolumes. Scaling up these membranes is feasible, with Inopor demonstrating the scale-up of their tubular ceramic nanofiltration membranes in a pilot plant containing 234 m^2^.^72^ Moreover, membrane filtration is a low-energy, low-labor-intensive unit operation.^35^ Ceramic membranes may also have their performance further optimized through surface functionalization.^73,74^ Improved performance is anticipated at an industrial scale under optimized membrane parameters, improved hydrodynamic conditions, and product-specific optimization of the reaction and diafiltration conditions as previously discussed.

The 4-arm branched PEGs used in this work were soluble in acetonitrile during all reaction steps (>18 mg/mL), and their branched nature facilitated high product rejection during membrane purification. We found that a larger 20 kDa PEG improved oligonucleotide solubility, enabling synthesis up to 18mer (**9**) using the OSU membrane. Fortunately, this larger support also achieved very high product rejections (99.5–100%) and halved the number of diavolumes needed compared to the OSN membrane used in this study without sacrificing purity or yield. Using a higher molecular weight support reduces oligonucleotide loading capacity per gram of support; however, the reduction in process time and solvent consumption may offset this limitation, especially if the support can be recovered and reused.^75^

The method presented here is complementary to emerging templated enzymatic ligation or convergent assembly methods.^6,76^ Crude fragments (∼3–10mer) could be synthesized using our LPOS platform and ligated in aqueous solution. This may also facilitate the removal of further downstream purification, as ligase enzymes are highly specific and can exclude impure shortmer fragments during assembly.^77,78^ In a convergent approach, blockmer (∼2–5mer) phosphoramidites can be synthesized using our platform by incorporating an orthogonal linker (base and acid stable) instead of the succinyl linker used in this work.^9,77–82^ This approach is beneficial in terms of isolating impurities to smaller fragments and achieving higher yields and purities compared with linear assembly.

## 4 Conclusions

This work presents the first one-pot liquid-phase oligonucleotide synthesis (OP-LPOS) process integrated with organic solvent nanofiltration (OSN) and ultrafiltration (OSU) membrane filtration, which was demonstrated through the production of 6mer and 18mer 2’-OMe phosphorothioate oligonucleotides, although it may be possible to extend to other chemistries. This innovative approach enables the addition of nucleotides through sequential coupling, sulfurization, and detritylation, followed by a single membrane-based purification step. Furthermore, this represents the first use of ceramic ultrafiltration in this application, which was found to be highly productive with a permeance of 13 Lm^2–^h^–1^bar^–1^ achieved compared to 2 Lm^2–^h^–1^bar^–1^ for the ceramic OSN membranes used herein.

Oligonucleotides were synthesized on a 4-arm branched PEG support (5 or 20 kDa), which enabled high product membrane rejection (99.5–100%) and therefore high stepwise filtration yields (97–100%). These membranes effectively removed all byproducts and impurities generated during one-pot synthesis to >97% stepwise purity with as low as 5 diavolumes using an ultrafiltration membrane. The PEG-oligonucleotide products exhibited high solubility in acetonitrile, and solubility could be further enhanced by adding methanol to improve filtration performance, especially for longer chain lengths where solubility becomes a limiting factor at the membrane interface. Membrane performance may be further enhanced by increasing cross-flow velocities across the membrane, improving product solubility at longer chain lengths through solvent choice and PEG molecular weight, and by removing the poorly soluble Dmtr-CySH impurity, which was the most challenging solute to purge.

We believe that this method is a potential scalable alternative to SPOS for the large-scale manufacturing of therapeutic oligonucleotides. Compared to the current membrane-assisted LPOS, this method is beneficial as it provides intermediates of higher purity and yield after diafiltration while needing fewer unit operations and diavolumes, resulting in decreased cycle times and a lower PMI. Our work is further amenable to emerging enzymatic ligation methods using oligo fragments (∼3–10mer), which offer purity enrichment and a possible solution to the solubility limitations of LPOS generally.

## Supplementary Material



## Notes

Materials, analytical techniques, details of membranes and membrane modules, synthetic procedures and loading calculation for the functionalization of PEG, screening data for ceramic and polymeric membranes, additional membrane-assisted LPOS results, detailed membrane filtration results for each synthesis step, distillation simulation, spiking and solvent screening OP-LPOS experiment data, and NMR and MALDI-TOF characterization (PDF)
