# Supplementary material for: Scalable Membrane Enabled One-Pot Liquid-Phase Oligonucleotide Synthesis
Source: Org Process Res Dev. 2025 May 13;29(6):1577–92. doi: 10.1021/acs.oprd.5c00117 (PMC12186683; doi:10.1021/acs.oprd.5c00117)
Supplement: Supplementary file 1 [file op5c00117_si_001.pdf]

Supporting Information for:
Scalable Membrane Enabled One-Pot Liquid-Phase Oligonucleotide Synthesis

Ronan Kelly[1,2], Catalina Parga[1,2], and Steven Ferguson*[1,2,3]
[1]*Department of Chemical and Bioprocess Engineering, University College Dublin, Dublin 4, Ireland*
[2]*SSPC, The Research Ireland Centre for Pharmaceuticals, School of Chemical and Bioprocess Engineering, University College Dublin, Dublin 4, Ireland*
[3]*National Institute for Bioprocess Research and Training, Foster Avenue, Mount Merrion, Blackrock, Co. Dublin, A94 X099, Ireland*

**Email: steven.ferguson@ucd.ie*

Table of Contents

Table of Contents

Supporting Information 1

Table of Contents 1

1 Materials and Methods 3

1.1 Materials 3

1.2 Membrane Modules 4

1.3 Analytical Methods 5

2 Functionalisation of PEG Support with First Nucleoside 6

3 Ceramic Membrane Screening 13

4 PEG Loading Calculation 15

4.1 Loading Calculation by MALDI-TOF 15

4.2 Loading Calculation by NMR 15

4.3 Oligonucleotide Loading onto 4-arm PEG Supports 17

5 LPOS Applied to Commercial Polymeric Membranes 17

6 Two-Pot Chain Extension Applied to NF 750 Da Membrane 20

6.1 Comments on Literature Two-Pot Synthesis 22

7 By-Products After Sulfurization and Amidite Quench 23

8 HPLC Monitoring of Detritylation Step 24

9 Example ^{31}P NMR Before and After Diafiltration 25

10Filtration Data with NF 750 Da Membrane 25

11 Filtration Data with UF 2000 Da Membrane 26

12 Transmembrane Pressure Excursion Study 29

13 Methanol Distillation Solvent Swap (DynoChem Simulation) 30

14 Dmtr-CySH Spiking Experiment and Solvent Screening 34

15 Acetonitrile/Sulfolane Mixed Solvent Screening During Membrane Filtration 35

References 37

NMR Spectra 38

MALDI-TOF 51

1 Materials and Methods

1.1 Materials

Dichloromethane (HPLC Grade), acetonitrile (HPLC Grade), pyridine, ethanol, methanol, dimethylformamide (DMF), sulfolane and isopropanol were purchased from Fisher or Sigma Aldrich. Solvents were mostly purchased anhydrous (Sure/SealTM from Sigma) or dried over 3 Å molecular sieves from Fisher Scientific UK. Molecular sieves were purchased from Sigma Aldrich. 4-arm Hydroxyl PEGs were purchased from Creative PEGWorks. 2'-O-Methyluridine, N,N'-Dicyclohexylcarbodiimide (DCC), triethylamine, succinic anhydride, N,N-Dimethyl-N'-(3-thioxo-3H-1,2,4-dithiazol-5-yl)formimidamide (DDTT), 1,1,1,3,3,3-Hexafluoro-2-propanol (HFIP), 4-dimethylaminopyridine (DMAP) and 4,4-dimethoxytrityl chloride were purchased from Fluorochem UK. Dichloroacetic acid (DCA), N-methylimidazole (NMI), 2,6-Dichlorobenzoyl chloride (DcbCl), diethylamine, anhydrous Na_2SO_4, citric acid, 2,6-lutidine, 1-dodecanethiol, 4,5-dicyanoimidazole (DCI), $NaHCO_3$, Bis(phenylacetyl) disulfide (PADS), and ammonium acetate were purchased from Fisher Scientific UK. Xanthane hydride (XH) and Bis(phenylacetyl) disulfide (PADS) were purchased from Tokyo Chemical Industry. Dmtr-2'O-Methyl-rG(ib) phosphoramidite, Dmtr-2'O-Methyl-rA(bz) phosphoramidite, Dmtr-2'O-Methyl-r phosphoramidite, Dmtr-2'O-Methyl-rC(ac) phosphoramidite, triethylamine (NEt_3), deuterated chloroform ($CDCl_3$), 2',4',6'-Trihydroxyacetophenone monohydrate (THAP), Phenyl-3H-1,2,4-dithiazol-3-one (POS), cyclohexanethiol (CySH), pyrrole, N-(3-dimethylaminopropyl)-N'-ethylcarbodiimide and acetic anhydride was purchased from Sigma Aldrich.

1.2 Membrane Modules

Supplier ceramic membrane modules: FG-M1-10x0250-PN40-SV and FG-M1-25x0250-PN40-SV modules supplied by FRTJ GmbH for 10 mm and 25 mm OD membranes with 250 mm length shown in Figure S1. Stainless steel modules are rated to 40 bar and 80 ^{o}C. Permeate and retentate ports are fitted with 8 mm and 12 mm compression fittings. Kalrez O-rings provided necessary seal at each end of the membrane upon tightening flanges.

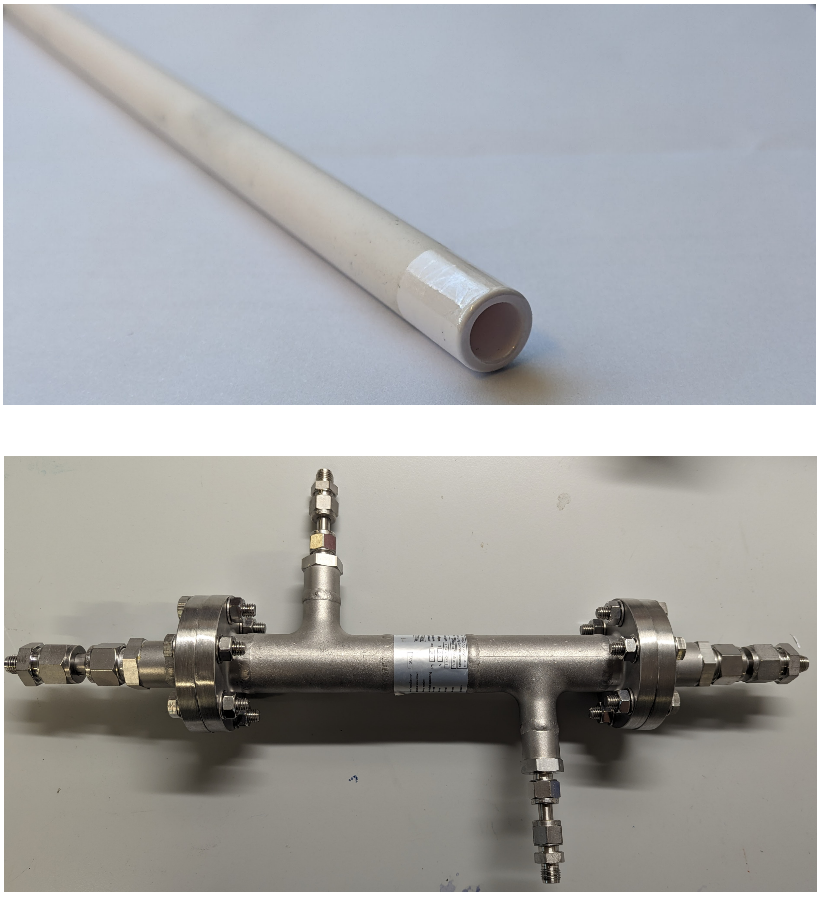

Figure S1: (Top) 1-channel ceramic membrane with glass end sealing. (Bottom) Stainless steel supplier building modules for ceramic membranes.

Custom flat-sheet membrane module: An adapted version of a mixed flow-through cell by Livingston and co-workers was manufactured by Parga for housing polymeric membranes.[105,164] The housing holds a flat-sheet membrane (7.55 cm^2 effective membrane area) which is cut into a disc to fit housing. A stir bar is placed inside to generate turbulent flow set to 600 rpm. A CAD drawing of the polymeric module is given in Figure S2.

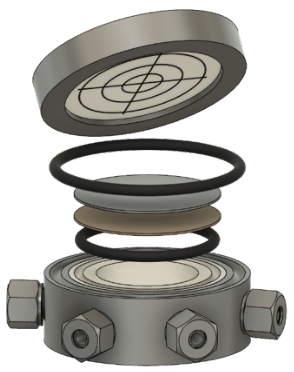

Figure S2: Stirred-cell for flat sheet ceramic membranes

1.3 Analytical Methods

Thin layer chromatography (TLC) was performed on Merck pre-coated Kieselgel 60F254 aluminium plates realised with UV irradiation. Flash column chromatography was performed on Fluorochem Silicagel 60 Å with particle size of 40-63µ. Reactions were carried out under N_2 atmosphere unless otherwise stated. Oxygen free nitrogen was obtained from BOC gases and was used without further drying.

HPLC analysis was performed on an Agilent Technologies 1100 Infinity system equipped with an auto-sampler and Agilent DAD detector. Standard analysis of reactions and filtrations used a gradient of 100mM aqueous ammonium acetate and MeOH (20 to 100%, 18 mins) eluted through Thermo Scientific BDS Hypersil C18 (150 mm x 4.6 mm) column at 40°C. The injection volume varied depending on the concentration of the sample. The pump flow rate was set to 1.0 mL/min and UV wavelength was set at 260 nm. After global deprotection and cleavage crude oligonucleotides were analysed with a gradient of 8.6 mM TEA/100 mM HFIP in DI water and MeOH (3 to 50%, 12 mins) eluted through a Waters XBridge® C18 (5 µm, 2.1 x 100 mm) at 40°C. The pump flow rate was set to 0.2 mL and and UV wavelength was set at 260 nm. The HPLC analysis purity data was reported in relative area %.

Typically, the NMR spectra were recorded at 25 °C on Varian VNMRS 400 MHz. Chemical shifts are recorded as δ-values in ppm relative to internal standard trimethylsilane (TMS) for ^{1}H and ^{13}C.

High Resolution Mass Spectrometry (HRMS) was carried out using an Agilent 6546 Quadrupole Time-of-Flight High Resolution Mass Spectrometer and associated Infinity Prime II 800bar HPLC system with variable wavelength detector (VWD).

Matrix assisted laser desorption ionisation (MALDI) was carried out on a Bruker Daltonics autolex Max TOF/TOF MALDI TOF machine. Samples were dissolved in MeCN (1-2 mg/mL) and mixed with an equal volume of matrix solution (1 µL). The matrix solution was a 20mg/mL solution of THAP dissolved in acetonitrile. The mixed solution (1 µL) was spotted onto a stainless-steel plate using the premixed technique and allowing the spot to dry uniformly by evaporation at room temperature.

2 Functionalisation of PEG Support with First Nucleoside

2.1 2'-O-methyl-5′-O-(4'',4'''-dimethoxytriphenylmethyl)uridine triethylammonium salt

2’-O-Methyluridine (2 g, 7.745 mmol) was co-evaporated with anhydrous acetonitrile (3 x 10 mL), dissolved in pyridine (20 mL) and 4,4′-dimethoxytrityl chloride (3.15 g, 9.294 mmol) was added. Reaction mixture was a strong orange colour at the start and was left stirring overnight at room temperature under N_2. The next day the reaction was yellow. MeOH (10 mL) was added dropwise top quench the reaction over 20 mins. Pyridine content was reduced by co-evaporation with toluene (200 mL). This was then diluted with DCM (150 mL) and extracted with $NaHCO_3$ (150 mL), then back extracted with DCM (3 x 50 mL) and dried over Na_2SO_4. Solvent was removed under reduced pressure to give a sticky, fluffy white product. Product was purified by column chromatography using DCM:Et_3N (98:2 v/v) as eluent. Solvent was removed under reduced pressure to give a frothy white product (4.255 g, 98% yield). R_f (CH_2Cl_2 + Et_3N) 0.2.

^{1}H NMR (400 MHz, $CDCl_3$) δ = 7.95 (d, J = 8.2 Hz, 1H, U C***H***), 7.45 – 7.25 (m, 2H, Dmtr ***H**$_{ar}$*), 7.30 – 7.15 (m, 7H, Dmtr ***H**$_{ar}$*), 6.84 – 6.76 (m, 4H, Dmtr ***H**$_{ar}$*), 5.94 (d, J = 2.0 Hz, 1H, ***H***-C(1’)), 5.22 (d, J = 8.1 Hz, 1H, U C***H***), 4.44 (dd, J = 7.4, 5.2 Hz, 1H, ***H***-C(3’)), 3.99 (dt, J = 7.5, 2.5 Hz, 1H, ***H***-C(4’)), 3.81 – 3.73 (m, J = 0.9 Hz, 8H, ***H***-C(2’), Dmtr-OC***H$_3$***), 3.62 (s, 3H, 2’OC***H$_3$***), 3.55 – 3.43 (m, 2H, J = 2.6 Hz, ***H***-C(5’)). ^{13}C NMR (101 MHz, $CDCl_3$) δ = 163.2 (U ***C***), 158.7 (Dmtr ***C***), 150.1 (U

C), 144.3 (Dmtr ***C***), 140.0 (U ***C***H), 135.3 (Dmtr ***C***), 135.0 (Dmtr ***C***), 130.2 (Dmtr ***C***H), 130.1 (Dmtr ***C***H), 128.1 (Dmtr ***C***H), 128.0 (Dmtr ***C***H), 127.1 (Dmtr ***C***H), 113.3 (Dmtr ***C***H), 102.1 (U ***C***H), 87.0 (Dmtr ***C***), 86.9 (***C***(1')), 84.0 (***C***(2')), 83.3 (***C***(4')), 68.4 (**C**(3')), 61.34 (***C***(5')), 58.7 (2'O***C***H_3), 55.2 (Dmtr O***C***H_3).

HRMS (ESI+) m/z calc. $[C_{31}H_{33}N_2O_8]^+$ $[M+H]^+$: 561.2238; found 561.2228.

2.2 2'-O-methyl-3'-O-succinyl-5'-O-(4'',4'''-dimethoxytriphenylmethyl)uridine triethylammonium salt, 5

5'-O-Dmtr-2'-O-methyluridine triethylammonium salt (4.34 g, 7.74 mmol) was co-evaporated with anhydrous acetonitrile (3 x 10 mL) and dissolved in pyridine (12.5 mL). DMAP (0.473 g, 3.87 mmol) and succinic anhydride (1.162 g, 11.61mmol) were added and the reaction was left stirring overnight at room temperature under N_2. The reaction mixture was cloudy yellow at the start of the reaction and brown the next day. Pyridine content was reduced by co-evaporation with toluene (150 mL). This was then diluted with DCM (150 mL) and extracted ice-cold 10% w/w citric acid (100 mL) and deionised water (100 mL) and dried over Na_2SO_4. Solvent was removed under reduced pressure to give a brown oil. Product was purified by column chromatography using DCM:MeOH:Et_3N (97-93:1-5:2 v/v) gradient as eluent. Gradient was increased every 200-300 mL (1% MeOH to 5% MeOH). Solvent was removed under reduced pressure to give a frothy white product (4.753 g, 93% yield). R_f (CH_2Cl_2:MeOH:Et_3N, 93:5:1) 0.2.

^{1}H NMR (400 MHz, $CDCl_3$) δ = 7.84 (d, J = 8.2 Hz, 1H, U C***H***), 7.37 – 7.30 (m, 2H, Dmtr ***H_{ar}***), 7.32 – 7.19 (m, 7H, Dmtr ***H_{ar}***), 6.87 – 6.78 (dd, 4H, Dmtr ***H_{ar}***), 5.99 (d, J = 3.8 Hz, 1H, ***H***-C(1')), 5.60 (s, 2H), 5.32 – 5.23 (m, 2H, U C***H, H***-C(3')), 4.29 – 4.18 (m, 1H, ***H***-C(4')), 4.06 (dd, J = 5.1, 3.9 Hz, 1H, ***H***-C(2')), 3.77 (s, 6H, Dmtr -OC***H_3***), 3.60 (q, *J* = 7.3 Hz, C*H_3*C***H_2***N^+), 3.50 (dd, J = 11.2, 2.3 Hz, 1H, ***H***-C(5')), 3.44 (s, 4H, 2'OC***H_3, H***-C(5')), 2.66 (t, J = 7.5 Hz, 2H, Suc C***H_2***), 2.52 (dd, J = 7.6, 6.3 Hz, 2H, Suc C***H_2***), 1.44 (t, *J* = 7.3 Hz, C***H_3***C*H_2*N^+), 1.14 (t, *J* = 7.3 Hz, C***H_3***C*H_2*N^+).^{13}C NMR (101 MHz, $CDCl_3$) δ = 177.4 (Suc ***C***=O), 173.1 (Suc ***C***=O), 163.0 (U ***C***), 158.7 (d, J = 2.3 Hz, Dmtr ***C***), 150.15 (U ***C***), 144.1 (Dmtr ***C***), 139.8 (U ***C***H), 135.1 (Dmtr ***C***) , 134.9 (Dmtr ***C***), 130.1 (2C, Dmtr ***C***H), 130.1 (2C, Dmtr ***C***H), 128.1 (d, J = 7.3 Hz, 2C, Dmtr ***C***H), 127.2 (Dmtr ***C***H), 113.3 (d, J = 2.9 Hz, Dmtr ***C***H), 102.3 (U ***C***H), 87.2 (Dmtr ***C***), 87.2 (***C***(1')), 82.2 (***C***(2')), 81.2 (***C***(4')), 69.8 (**C**(3')),

63.4 (CH$_3$**C**H$_2$N$^+$), 61.7 (**C**(5’)), 59.0 (2’O**C**H$_3$), 52.9 (CH$_3$**C**H$_2$N$^+$), 45.4 (CH$_3$**C**H$_2$N$^+$), 31.9 (Suc **C**H$_2$), 30.6 (Suc **C**H$_2$), 9.7 (**C**H$_3$**C**H$_2$N$^+$), 8.1 (**C**H$_3$CH$_2$N$^+$).

HRMS (ESI$^+$) m/z calc. $[C_{35}H_{36}N_2O_{11}]^+$ $[M]^+$: 660.2319; found 660.2301.

2.3 5 kDa 4-arm PEG-O-mU-ODmtr, 1b

5 kDa 4-arm PEG **1a** (2.875 g, 0.927 mmol) was co-evaporated with anhydrous acetonitrile (3 x 8 mL). 5’-O-Dmtr-3’-O-succinyl-2’-O-methyluridine triethylammonium salt **5** (2.23 g, 2.927 mmol) was co-evaporated with anhydrous acetonitrile (3 x 7 mL). 5’-O-Dmtr-3’-O-succinyl-2’-O-methyluridine was dissolved in anhydrous DCM (7 mL), N-Methylimidazole (0.494 mL, 6.193 mmol) and 2,6-dichlorobenzoyl chloride (0.57 mL, 3.941 mmol) were added and left stirring for 20 mins to activate the sugar. 5 kDa 4-arm PEG **1a** was dissolved with DCM (14 mL) and added dropwise to the activated sugar. The reaction was left at room temperature overnight under N_2, the reaction mixture was colourless at start of reaction and slightly brown after 24 hours. Diluted with DCM (150 mL) and extracted with $NaHCO_3$ (100 mL), back extracted with DCM (3 x 50 mL). Dried over Na_2SO_4 and solvent content reduced under reduced pressure. Product was precipitated with ice-cold cold diethyl ether (200 mL) and washed with cold diethyl ether. Solvent was removed under reduced pressure to give a sticky slightly brown product (3.636 g, 83%).

^{1}H NMR (400 MHz, CDCl$_3$) δ = 7.84 (d, J = 8.2 Hz, 4H, U C**H**), 7.38 – 7.20 (m, 36H, Dmtr ***H**$_{ar}$*), 6.84 (d, J = 9.0 Hz, 16H, Dmtr ***H**$_{ar}$*), 6.01 (d, J = 3.9 Hz, 4H, **H**-C(1’)), 5.34 – 5.21 (m, 8H, U C**H**, **H**-C(3’)), 4.27 – 4.19 (m, 12H, **H**-C(4’), Suc-OC**H**$_2$), 4.07 (dd, 4H, **H**-C(2’)), 3.79 (s, 24H, Dmtr-OC**H**$_3$), 3.69 – 3.51 (m, 464H, C**H**$_2$O), 3.49 – 3.38 (m, 16H, 2’OC**H**$_3$, **H**-C(5’)), 2.71 – 2.62 (m, 16H, Suc C**H**$_2$), 2.53 (q, J = 7.0 Hz, CH$_3$C**H**$_2$N$^+$), 1.02 (t, J = 7.2 Hz, C**H**$_3$CH$_2$N$^+$). ^{13}C NMR (101 MHz, CDCl$_3$) δ = 172.2 (4C, Suc **C**=O), 171.8 (4C, Suc **C**=O), 163.0 (4C, U **C**), 159.2 (8C, Dmtr **C**), 150.3 (4C, U **C**), 144.5 (4C, Dmtr **C**), 140.1 (4C, U **C**H), 135.3 (d, J = 11.7 Hz, 8C, Dmtr **C**H)), 130.5 (d, J = 7.5 Hz, 16C, Dmtr **C**H), 128.5 (d, J = 4.7 Hz, 16C, Dmtr **C**H), 127.6 (4C, Dmtr **C**H), 113.7 (16C, Dmtr **C**H), 102.9 (4C, U **C**H), 87.8 (4C, Dmtr **C**), 87.5 (4C, **C**(1’)), 82.6 (4C, **C**(2’)), 81.5 (4C, **C**(4’)), 70.9 (232C, **C**H$_2$O), 69.4 (4C, **C**(3’)), 64.3 (4C, **C**H$_2$O), 62.0 (4C, **C**(5’)), 59.4 (4C, 2’O**C**H$_3$), 55.7 (8C, Dmtr **C**H), 46.4 (CH$_3$**C**H$_2$N$^+$), 29.3 (d, J = 4.2 Hz, (8C, Suc **C**H$_2$)), 11.7 (**C**H$_3$CH$_2$N$^+$).

MALDI-TOF analysis: Average MW calc. 7747; found 7532.

2.4 5 kDa 4-arm PEG-O-mU-OH, 1c

5 kDa 4-arm PEG-mU-ODmtr **1b** (4.317 g) was co-evaporated with anhydrous acetonitrile (3 x 20 mL), dissolved in anhydrous acetonitrile (15 mL) and cooled to 0 °C. 2,6-lutidine (0.15 mL, 1% v/v), NMI (0.15 mL, 1% v/v) and acetic anhydride (0.15 mL, 1% v/v) were added and left stirring for 10 mins. Product precipitated from ice-cold diethyl ether (250 mL) to give a yellow sticky solid (4.4 g).

5 kDa 4-arm PEG-mU-ODmtr from previous step (4.4 g) was co-evaporated with anhydrous acetonitrile (3 x 20 mL), dissolved in anhydrous DCM (15 mL). Pyrrole (0.45 mL, 3% v/v) and DCA (0.75 mL, 5% v/v) were added dropwise and left stirring at room temperature. Colour turned deep red and dissipated over 30 mins. Product precipitated from ice-cold diethyl ether (250 mL) to give a brown glassy solid (2.954 g).

^{1}H NMR (400 MHz, $CDCl_3$) δ = 8.77 (s, 4H, 5'-O***H***), 7.81 (d, J = 8.2 Hz, 4H, U C***H***), 7.39 – 7.27 (m, **Dcb-ester**), 5.81 (d, J = 5.1 Hz, 4H, ***H***-C(1')), 5.73 (d, J = 8.2 Hz, 4H, ***H***-C(5')), 5.31 (t, J = 4.7 Hz, 4H, ***H***-C(3')), 4.28 – 4.16 (m, 16H, ***H***-C(4')), Suc-OC$\boldsymbol{H}_2$, ***H***-C(2')), 3.96 – 3.87 (m, 4H, ***H***-C(5')), 3.84 – 3.73 (m, 4H, ***H***-C(5')), 3.71 – 3.50 (m, 464H, C$\boldsymbol{H}_2$O), 3.48 – 3.38 (m, 12H, 12H, 2'OC$\boldsymbol{H}_3$), 2.76 – 2.60 (m, 16H, Suc C$\boldsymbol{H}_2$).^{13}C NMR (101 MHz, $CDCl_3$) δ = 172.1 (4C, Suc ***C***=O), 171.8 (4C, Suc ***C***=O), 163.0 (4C, U ***C***), 150.3 (4C, U ***C***), 141.4 (4C, U ***C***H), 102.8 (4C, U ***C***H), 89.4 (4C, ***C***(1')), 83.3 (4C, ***C***(2'/4')), 81.4 (4C, ***C***(2'/4')), 70.6 (232C, ***C***H_2O), 69.1 (4C, ***C***(3')), 64.0 (4C, ***C***H_2O), 61.5 (4C, ***C***(5')), 59.1 (4C, 2'O***C***H_3), 29.2 (8C, Suc ***C***H_2).

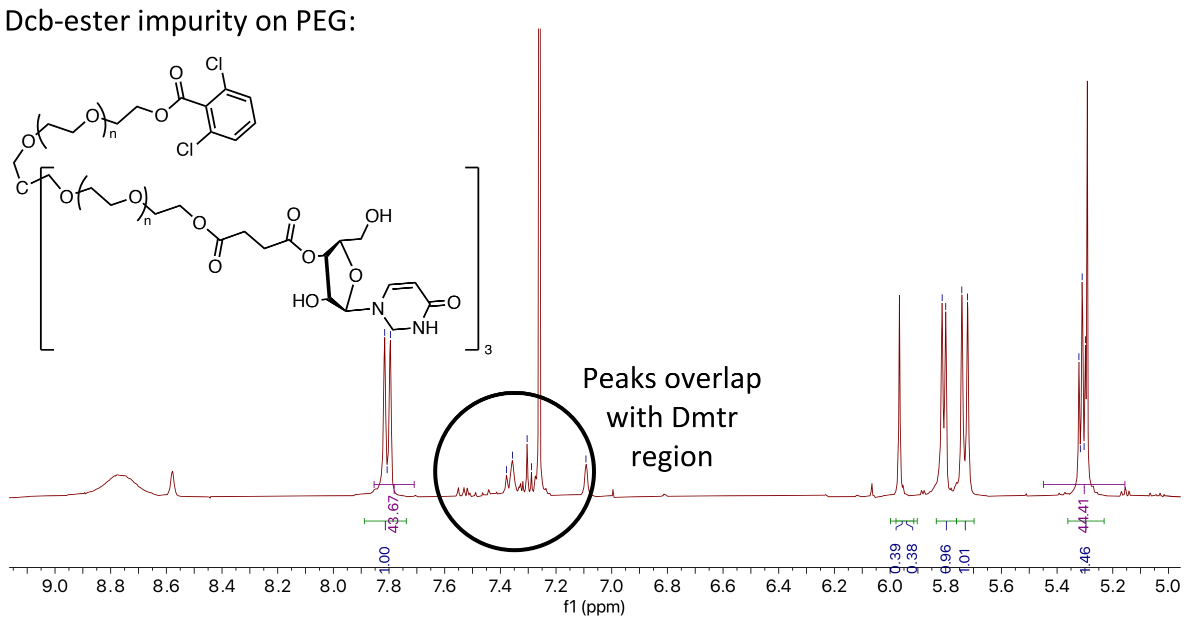

Figure S3: ^{1}H NMR spectra of detritylated PEG-mU-OH with Dcb-ester region identified. Peaks overlap with Dmtr peaks ~7.3 ppm, obscuring NMR analysis after detritylation reaction. Appears as if some Dmtr remains on 5'-OH end.

2.5 20 kDa 4-arm PEG-O-mU-ODmtr, 3b

20 kDa 4-arm PEG **3a** (4 g, 0.191 mmol) was co-evaporated with anhydrous acetonitrile (3 x 18 mL). 5'-O-Dmtr-3'-O-succinyl-2'-O-methyluridine triethylammonium salt **5** (1.74 g, 2.29 mmol) was co-evaporated with anhydrous acetonitrile (2 x 8 mL). 5'-O-Dmtr-3'-O-succinyl-2'-O-methyluridine **5** and 20 kDa 4-arm PEG **3a** were dissolved in anhydrous DCM (24 mL). N,N'-Dicyclohexylcarbodiimide (0.52 g, 2.52 mmol) and 4-Dimethylaminopyridine (0.308 g, 2.52 mmol) were added and the reaction was left stirring under N_2 at 0°C and left to slowly heat up to room temperature overnight under N_2. Stirred for 24 hours in total. Product was precipitated with ice-cold cold diethyl ether (250 mL). Product was washed with diethyl ether and solvent was removed under reduced pressure to give a frothy pale pink product (4.28 g, 95%).

^{1}H NMR (400 MHz, $CDCl_3$ + 1% v/v NEt_3) δ = 7.81 (d, *J* = 8.2 Hz, 4H, U C***H***), 7.36 – 7.19 (m, 36H, Dmtr ***H**~ar~*), 6.82 (d, *J* = 9.0 Hz, 16H, Dmtr ***H**~ar~*), 6.00 (d, *J* = 3.9 Hz, 4H, ***H***-C(1')), 5.30 – 5.24 (m, 8H, U C***H, H***-C(3')), 4.21 (m, 12H, ***H***-C(4'), Suc-OC$\boldsymbol{H}_2$), 4.09 – 4.03 (dd, 4H, ***H***-C(2')), 3.80 (s, *J* = 4.7 Hz, 24H, Dmtr-OC$\boldsymbol{H}_3$), 3.71 – 3.49 (m, 1908H, CH_2O), 3.48 – 3.37 (m, 16H, 2'OC$\boldsymbol{H}_3$, ***H***-C(5')), 2.69 – 2.56 (m, 16H, Suc C$\boldsymbol{H}_2$), 2.50 (q, *J* = 7.2 Hz, CH_3C$\boldsymbol{H}_2$$N^+$), 1.00 (t, *J* = 7.2 Hz, C$\boldsymbol{H}_3$C$H_2$$N^+$). ^{13}C

NMR (101 MHz, $CDCl_3$ + 1% v/v NEt_3) δ = 172.0 (4C, Suc ***C***=O), 171.6 (4C, Suc ***C***=O), 162.9 (4C, U ***C***), 158.9 (8C, Dmtr ***C***), 150.2 (4C, U ***C***), 144.2 (4C, Dmtr ***C***), 139.7 (4C, U ***C***H), 135.0 (8C, Dmtr ***C***H), 130.2 (16C, Dmtr ***C***H), 128.2 (16C, Dmtr ***C***H), 127.3 (4C, Dmtr ***C***H), 113.4 (16C, Dmtr ***C***H), 102.6 (4C, U ***C***H), 87.5 (4C, Dmtr ***C***), 87.1 (4C, ***C***(1')), 82.2 (4C, ***C***(2')), 81.2 (4C, ***C***(4')), 70.7 (954C, ***C***H_2O), 69.1 (4C, ***C***(3')), 64.0 (4C, ***C***H_2O), 61.8 (4C, ***C***(5')), 59.1 (4C, 2'O***C***H_3), 55.3 (8C, Dmtr ***C***H), 46.3 (CH_3***C***H_2N^+), 29.0 (8C, Suc ***C***H_2), 11.7 (***C***$H_3CH_2N^+$)

MALDI-TOF analysis: Average MW calc. 23608; found 24103.

2.6 20 kDa 4-arm PEG-O-mU-OH, 3c

20 kDa 4-arm PEG-mU-ODmtr **3b** (4.45 g) was co-evaporated with anhydrous acetonitrile (1 x 20 mL), dissolved in anhydrous acetonitrile (20 mL) and cooled to 0 °C. 2,6-lutidine (2 mL, 10% v/v), NMI (2 mL, 10% v/v) and acetic anhydride (2 mL, 10% v/v) were added and left stirring for 10 mins. Product precipitated from ice-cold diethyl ether (250 mL) to give a pale pink solid (4.4 g).

20 kDa 4-arm PEG-mU-ODmtr from previous step (4.4 g) was co-evaporated with anhydrous acetonitrile (2 x 20 mL), dissolved in anhydrous DCM (20 mL). Pyrrole (0.6 mL, 3% v/v) and DCA (1 mL, 5% v/v) added dropwise and left stirring at room temperature. Colour turned deep red and dissipated over 30 mins. Product precipitated from ice-cold diethyl ether (250 mL) to give an off-white solid (3.824 g).

^{1}H NMR (400 MHz, $CDCl_3$) δ = 8.50 (s, 4H, 5'-O***H***), 7.78 (d, *J* = 8.2 Hz, 4H, U C***H***), 5.79 (d, *J* = 5.2 Hz, 4H, ***H***-C(1')), 5.71 (dd, *J* = 8.8 Hz, 4H, ***H***-C(5')), 5.31 (t, *J* = 4.7 Hz, 4H, ***H***-C(3')), 4.25 – 4.14 (m, 16H, ***H***-C(4'), Suc-OC***H***$_2$, ***H***-C(2')), 3.91 (dd, *J* = 12.4, 2.1 Hz, 4H, ***H***-C(5')), 3.82 – 3.73 (m, 4H, ***H***-C(5')), 3.62 (m, 1908H, C***H***$_2$O), 3.44 – 3.33 (m, 12H, 2'OC***H***$_3$), 2.75 – 2.64 (m, 16H, Suc C***H***$_2$). ^{13}C NMR (101 MHz, $CDCl_3$) δ = 172.1 (4C, Suc ***C***=O), 171.8 (4C, Suc ***C***=O), 162.7 (4C, U ***C***), 150.3 (4C, U ***C***), 141.3 (4C, U ***C***H), 102.8 (4C, U ***C***H), 89.4 (4C, ***C***(1')), 83.3 (4C, ***C***(2'/4')), 81.4 (4C, ***C***(2'/4')), 70.7 (954C, ***C***H_2O), 69.1 (4C, ***C***(3')), 64.0 (4C, ***C***H_2O), 61.5 (4C, ***C***(5')), 59.1 (4C, 2'O***C***H_3), 31.0 (4C, Suc ***C***H_2), 29.1 (4C, Suc ***C***H_2).

2.7 10 kDa 4-arm PEG-O-mU-ODmtr, 2b

2b was synthesised using the same procedure as **3b** using **5** (3.41 g, 4.483 mmol) and **2a** (4 g, 0.474 mmol) as starting materials to give a light pink solid (4.85 g, 97% yield).

^{1}H NMR (400 MHz, $CDCl_3$ + 1% v/v NEt_3) δ = 7.83 (d, J = 8.2 Hz, 4H, U C***H***), 7.38 – 7.18 (m, 36H, Dmtr $\boldsymbol{H_{ar}}$), 6.82 (dd, 16H), 6.00 (d, J = 3.9 Hz, 16H, Dmtr $\boldsymbol{H_{ar}}$), 5.37 – 5.19 (m, 8H, U C***H***, ***H***-C(3')), 4.25 – 4.18 (m, 12H, ***H***-C(4'), Suc-OC$\boldsymbol{H_2}$), 4.09 – 4.03 (m, 4H, ***H***-C(2')), 3.78 (s, 18H, *J* = 4.7 Hz, 24H, Dmtr-OC$\boldsymbol{H_3}$), 3.71 – 3.52 (m, 973H, C$\boldsymbol{H_2}$O), 3.47 – 3.39 (m, 16H, 2'OC$\boldsymbol{H_3}$, ***H***-C(5')), 2.71 – 2.61 (m, 16H, Suc C$\boldsymbol{H_2}$), 2.53 (q, J = 7.2 Hz, CH_3C$\boldsymbol{H_2}$$N^+$), 1.02 (t, J = 7.2 Hz, C$\boldsymbol{H_3}$C$H_2$$N^+$). ^{13}C NMR (101 MHz, $CDCl_3$) δ = 172.0 (4C, Suc ***C***=O), 171.6 (4C, Suc ***C***=O), 162.8 (4C, U ***C***), 158.8 (8C, Dmtr ***C***), 150.1 (4C, U ***C***), 144.2 (4C, Dmtr ***C***), 139.8 (4C, U ***C***H), 135.0 (d, J = 12.2 Hz, (8C, Dmtr ***C***H)), 130.2 (d, J = 7.4 Hz, 16C, Dmtr ***C***H), 129.2 (16C, Dmtr ***C***H), 128.1 (d, J = 4.6 Hz, 4C, Dmtr ***C***H), 113.4 (16C, Dmtr ***C***H), 102.5 (4C, U ***C***H), 87.5 (d, J = 31.2 Hz, (4C, Dmtr ***C***)), 87.1 (4C, ***C***(1')), 82.2 (4C, ***C***(2')), 81.3 (4C, ***C***(4')), 70.6 (487C, ***C***H_2O), 69.1 (4C, ***C***(3')), 64.0 (4C, ***C***H_2O), 61.7 (4C, ***C***(5')), 59.1 (4C, 2'O***C***H_3), 55.3 (8C, Dmtr ***C***H), 28.9 (8C, Suc ***C***H_2).

MALDI-TOF analysis: Average MW calc. 13346; found 13250.

2.8 10 kDa 4-arm PEG-O-mU-OH, 2c

2c was synthesised using the same procedure as **3c** to give an off-white solid (3.985 g).

^{1}H NMR (400 MHz, $CDCl_3$) δ = 8.58 (s, 4H, 5'-O***H***), 7.80 (d, J = 8.0 Hz, 4H, U C***H***), 5.81 (d, J = 5.3 Hz, 4H, ***H***-C(1')), 5.73 (d, J = 8.2 Hz, 4H, ***H***-C(5')), 5.34 – 5.24 (m, , 4H, ***H***-C(3')), 4.29 – 4.14 (m, 16H, ***H***-C(4')), Suc-OC$\boldsymbol{H_2}$, ***H***-C(2')), 3.92 (dd, J = 12.5, 2.0 Hz, 4H, ***H***-C(5')), 3.81 – 3.75 (m, 4H, C$\boldsymbol{H_2}$). ^{13}C NMR (101 MHz, $CDCl_3$) δ = 172.1 (4C, Suc ***C***=O), 171.8 (4C, Suc ***C***=O), 162.9 (4C, U ***C***), 150.3 (4C, U ***C***), 141.3 (4C, U ***C***H), 102.8 (4C, U ***C***H), 89.2 (4C, ***C***(1')), 83.2 (4C, ***C***(2'/4')), 81.5 (4C, ***C***(2'/4')), 70.6 (487C, ***C***H_2O), 69.1 (4C, ***C***(3')), 64.0 (4C, ***C***H_2O), 61.4 (4C, ***C***(5')), 59.0 (4C, 2'O***C***H_3), 31.0 (4C, 2'O***C***H_3), 29.1 (4C, Suc ***C***H_2).

2.9 40 kDa 4-arm PEG-O-mU-ODmtr, 4b

4b was synthesised using the same procedure as **3b** using **5** (0.4 g, 0.6 mmol) and **4a** (2 g, 0.05 mmol) as starting materials to give a grey solid (2.02 g, 95%).

MALDI-TOF analysis: Average MW calc. 43809; found 43003.

2.10 40 kDa 4-arm PEG-O-mU-OH, 4c

4c was synthesis using the same procedure as **3c** to give a glassy grey solid (1.72 g).

^{1}H NMR (400 MHz, $CDCl_3$) δ = 8.47 (s, 4H, 5′-O***H***), 7.83 (d, J = 8.2 Hz, 4H, U C***H***), 5.80 (d, J = 4.7 Hz, 4H, ***H***-C(1′)), 5.71 (d, J = 8.4 Hz, 4H, ***H***-C(5′)), 5.29 – 5.21 (m, 4H, ***H***-C(3′)), 4.24 – 4.10 (m, 16H, ***H***-C(4′)), Suc-OC$\boldsymbol{H}_2$, ***H***-C(2′)), 3.94 – 3.87 (m, 4H, ***H***-C(5′)), 3.82 – 3.75 (m, 4H, ***H***-C(5′)), 3.73 – 3.48 (s, 3743H, C$\boldsymbol{H}_2$O), 3.46 – 3.41 (m, 12H, 2′OC$\boldsymbol{H}_3$), 2.71 (m, 8H, Suc C$\boldsymbol{H}_2$), 2.51 (m, 8H, Suc C$\boldsymbol{H}_2$).

2.11 4,4′-Dimethoxytrityl (cyclohexyl) sulfane, Dmtr-CySH, 7

4,4′-Dimethoxytrityl chloride (1.49 g, 4.40 mmol) was dissolved in anhydrous pyridine (10 mL). Cyclohexanethiol (0.48 g, 4.00 mmol) was added and left stirring at room temperature overnight under N_2. Reaction was quenched with MeOH (10 mL) and co-evaporated with toluene (200 mL) to reduce pyridine content. Diluted with DCM (150 mL) and extracted with $NaHCO_3$ (100 mL), back extracted with DCM (50 mL), dried over Na_2SO_4 and solvent removed. Product was purified by column chromatography using DCM:MeOH:Et_3N (99-94:0-5:1 v/v) gradient as eluent. Solvent was removed under reduced pressure to give a yellow oil (1.403 g, 84% yield). R_f (CH_2Cl_2:Et_3N, 99:1) = 0.5.

^{1}H NMR (400 MHz, $CDCl_3$) δ = 7.52 – 7.11 (m, 9H, Dmtr $\mathbf{H}_{\mathbf{ar}}$), 6.88 – 6.76 (m, 4H, Dmtr $\mathbf{H}_{\mathbf{ar}}$), 3.79 (d, J = 5.1 Hz, 6H, Dmtr-OC$\boldsymbol{H}_3$), 2.22 – 2.11 (m, 1H, **H**-Cy), 1.64 – 0.94 (m, 9H, **H**-Cy). ^{13}C NMR (101 MHz, $CDCl_3$) δ = 158.0 (Dmtr ***C***), 146.2 (Dmtr ***C***), 138.0 (Dmtr ***C***), 130.9 (Dmtr ***C***H), 129.6 (Dmtr ***C***H), 129.3 (Dmtr ***C***H), 113.1 (Dmtr ***C***H), 66.5 (Dmtr ***C***), 55.4 (d, J = 2.3 Hz, Dmtr-O***C***H_3), 34.7 (**Cy**SH), 26.5 (**Cy**SH), 25.8 (**Cy**SH).

3 Ceramic Membrane Screening

Membrane screening of Inopor NF 750 Da MWCO (1 nm pore size) and UF 2000 Da MWCO (3 nm pore size) was conducted in acetonitrile, the solvent of choice for oligonucleotide synthesis. The test solutes for screening membranes were the 4-arm PEGs (5, 10, 20 and 40 kDa) loaded with the first deprotected nucleoside **1c**-**4c** and 5′-Dmtr-2′-OMe-G^{ib}

phosphoramidite (869.95 g/mol). 2'-OMe modified phosphoramidites were chosen for this project due to the presence of this protecting group in a number of oligonucleotide therapeutics on the market.[3] Phosphoramidites are the largest species that must be filtered, as incomplete removal would result in unwanted couplings in subsequent chain extension cycles, giving rise to addition (n+1) impurities.

The ceramic membranes remained stable throughout screening, with results summarised in Table S1. Both NF and UF membranes showed >99% rejection of loaded PEGs **1c-4c**. The NF 750 Da membrane achieved 100% rejection of 5 kDa loaded PEG **1c,** while the UF 2000 Da membrane with its larger pore size, required a 4-arm 40 kDa PEG **4c** for 100% rejection. Crucially, the UF 2000 Da membrane showed much lower amidite rejection (43.8%) and therefore higher selectivity compared to the NF 750 Da membrane (74.3%), which reduces the number of diavolumes required to prevent accumulation of phosphoramidites, thereby improving solvent efficiency of the separation process. Any membrane which has very high rejection of impurities would require too much solvent for a scalable process and therefore a balance in selectivity is needed for optimal diavolume efficiency. Additionally, the UF 2000 Da membrane exhibited significantly higher permeance (18 $Lm^{-2}h^{-1}bar^{-1}$) compared to the NF 750 Da membrane (4 $Lm^{-2}h^{-1}bar^{-1}$). Based on these promising results, both membranes were suitable for testing under LPOS reaction conditions.

Table S1: Membrane screening results for ceramic membranes

	**Inopor NF 750 Da**	**Inopor UF 2000 Da**
**Rejection (%)**		
5 kDa 4-arm PEG-mU-OH, **1c**	100±0.0	99.0±0.1
10 kDa 4-arm PEG-mU-OH, **2c**	100±0.0	99.2±0.2
20 kDa 4-arm PEG-mU-OH, **3c**	-	99.5±0.2
40 kDa 4-arm PEG-mU-OH, **4c**	-	100±0.0
Dmtr-2'-OMe-rG^{ib} Phosphoramidite (869.95 g/mol)	74.3±0.6	43.8±3.1
**Pressure (bar)**	10	8
**Permeance ($Lm^{-2}h^{-1}bar^{-1}$)**	4.4±0.7	18.0±1.9

4 PEG Loading Calculation

4.1 Loading Calculation by MALDI-TOF

Loading was calculated by the MALDI-MS spectrum of the unloaded PEG and oligonucleotide loaded PEG. The samples (1-2 mg/mL) were mixed an equal volume (1 µL) of matrix solution THAP 20 mg/mL in acetonitrile, spotted, air dried and analysed by MALDI-TOF operated in linear positive mode. The molecular weights of the loaded PEG and starting PEGs were determined using Bruker FlexAnalysis software by taking the peak maximum or by the average molecular weight (M_n) calculated by the software. An example loading (%) of 5 kDa PEG **1b** with first nucleoside was calculated:

$$Loading\ (\%) = \left(\frac{MW_{PEG-oligo} - MW_{PEG}}{MW_{Theoretical} - MW_{PEG}}\right) * 100 = \left(\frac{7532 - 5096}{(5096 + 4(659.67)) - 5096}\right) * 100 = 92\%$$

Equation 1

Where MW_{PEG} and $MW_{PEG\text{-}oligo}$ are the calculated molecular weights by MALDI-MS for 4-arm PEG before loading and after functionisation with first uridine nucleoside. $MW_{Theoretical}$ is the theoretical molecular weight assuming complete loading of first nucleoside.

4.2 Loading Calculation by NMR

NMR integration was also used to determine loading when unloaded nucleoside was fully removed according to HPLC. The ^{1}H-NMR peak of the DmtrH (6.8 ppm) or U-C*H* (7.8 ppm) was compared to 4-arm PEG core (3.6 ppm).[4] The number of protons for a given 4-arm PEG was calculated by dividing the MW given by supplier (e.g 20969 g/mol for 20 kDa PEG) by the MW of an ethylene glycol repeating unit (44 g/mol) to get the number of repeating units and then multiplying this value by 4 to give 1908H in this case. An example loading (%) is calculated for a 5 kDa PEG loaded with first nucleoside **13b**:

$$Loading\ (\%) = \frac{Integration\ of\ DmtrH}{Integration\ of\ PEG * \frac{16}{1908}} * 100\% = \frac{16}{1999.54 * \frac{16}{1908}} * 100\% = 95\%$$

Equation 2

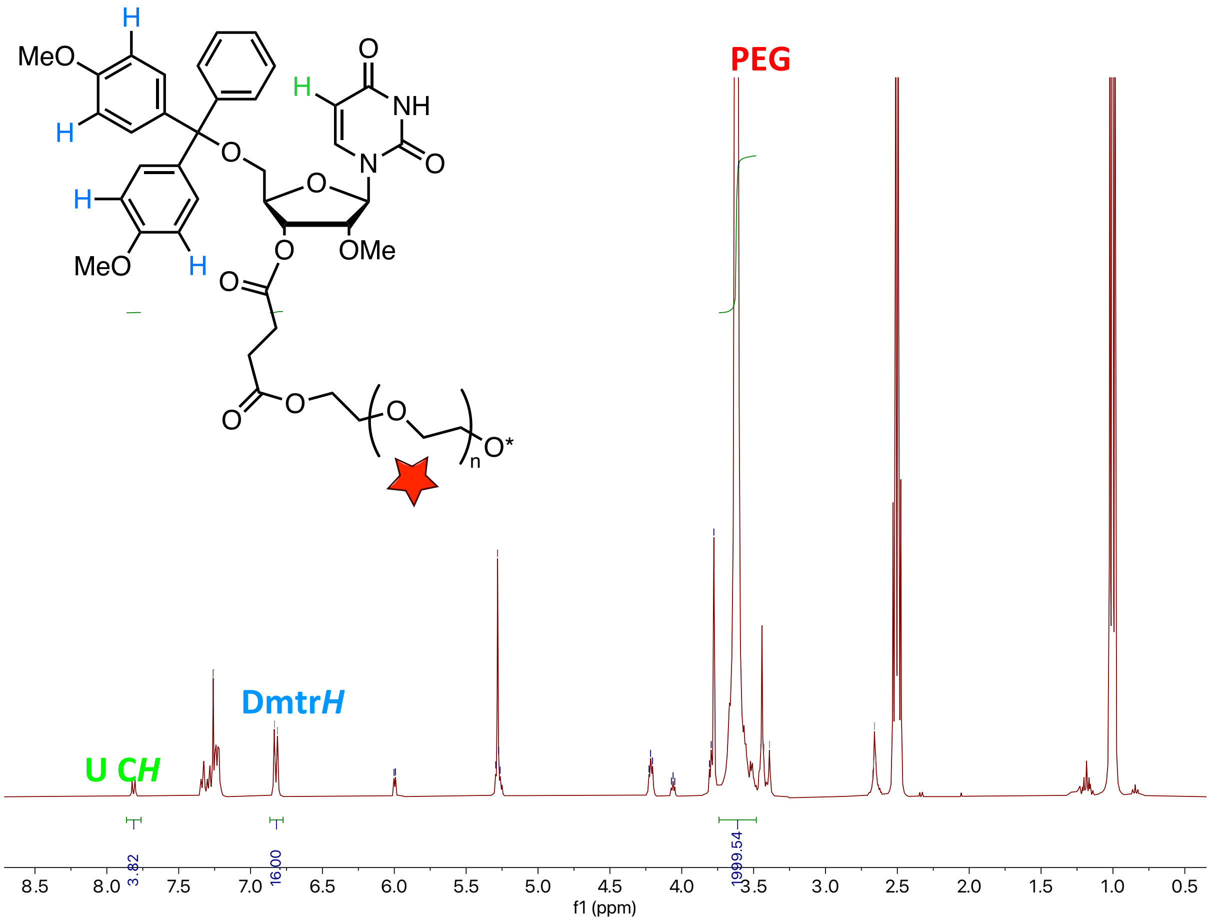

*Figure S4: ^{1}H NMR ($CDCl_3$) of loaded 20 kDa PEG-mU-ODmtr **3b** showing relevant DmtrH and PEG protons used for loading calculation.*

4.3 Oligonucleotide Loading onto 4-arm PEG Supports

Table S2: Loading calculation for first nucleoside onto various PEGs

Compound	Loading Chemistry	4-arm PEG Size (kDa)	MALDI-TOF % Loading	NMR % Loading	Oligo Loading (µmol/g) [(a)]
**1b**	DcbCl/NMI	5	92	93	478
**2b**	DCC/DMAP	10	94	95	283
**3b**	DCC/DMAP	20	91	95	158
**4b**	DCC/DMAP	40	86	81	75

(a) Based on average loading from MALDI and NMR calculation; reported as µmol oligo/g PEG

5 LPOS Applied to Commercial Polymeric Membranes

Rig set-up: The same membrane configuration was used method was used for screening polymeric membranes as ceramic membranes as described in the main text using a custom polymeric membrane housing detailed previously.

Several polymeric membranes were screened and OP-LPOS synthesis conditions outlined in in the main text or standard literature two-pot synthesis conditions were applied to commercial polymeric membranes which showed promising results during membrane screening. A summary of the membrane screening results and LPOS performance in each case is given in Table S3. Under reaction conditions the most promising membranes in Borsig oNF-3, SolSep UF, SolSep NF and Novamem PEEK5 which gave excellent results under screening had lower PEG-oligo rejection values than predicted during screening, oNF-3 was not stable under basic conditions and SolSep UF could not remove a small molecular weight by-product. Such chemical instability has been a common issue with polymeric membranes.[1] Based on these issues more stable ceramic membranes were explored for LPOS rather than exploring other polymeric membranes available on the market at the time.

Table S3: Summary of results from developed OP-LPOS applied to commercial polymeric membranes.

Membrane	4-arm PEG MW (kDa)	Synthesis	Result Summary	Conclusion
Borsig oNF-3	5/10	OP-LPOS	• 100% PEG rejection during screening.<br>• 10 kDa PEG-dimer rejection just 52.0% after 3.3 diavolumes hypothesized due to membrane swelling during pyridine acid quench.<br>• Rejection rose to 96.2% when an acid quench was excluded. 9.6 diavolumes required to deliver 90% purity by LC.	Chemically unstable under certain acid/base conditions. Product rejection insufficient; below 99%.
SolSep UF (010706)	40	OP-LPOS	• 100% PEG rejection during screening.<br>• By-product Py.TFA not removed. PEG-dimer rejection just 94.5%.<br>• All other by-products and impurities removed; LC purity 40%.	Poor purity and low product rejection. Remaining Py.TFA can act as a coupling reagent, resulting in uncontrolled coupling in subsequent chain extensions.
SolSep (NF10306)	10	Two-pot synthesis	• 100% PEG rejection during screening.<br>• 29% PEG-dimer rejection after coupling-sulfurization.	Yield too low under reaction conditions.

Membrane	4-arm PEG MW (kDa)	Synthesis	Result Summary	Conclusion
			• 82% PEG-dimer rejection after detritylation.<br>• 3 diavolumes required to remove impurities.	
Puramem Flux	N/A	N/A	• Phosphoramidite rejection >80%.	Phosphoramidite rejection high – not pursued further
Puramem Performance	N/A	N/A	• Phosphoramidite rejection >60%.	Phosphoramidite rejection high – not pursued further
Puramem S600	N/A	N/A	• Phosphoramidite rejection >80%.	Phosphoramidite rejection high – not pursued further
Duramem 900	N/A	N/A	• Phosphoramidite rejection >90%.	Phosphoramidite rejection high – not pursued further
Novamem (PEEK5)	20/40	Two-pot synthesis	• 100% PEG rejection during screening.<br>• 0% product yield after 3 diavolumes.	Highly unstable under reaction conditions

6 Two-Pot Chain Extension Applied to NF 750 Da Membrane

A standard two-pot approach was also attempted with NF 750 Da membrane to synthesise a dimer (PEG-mUmC^{ac}-OH) oligonucleotide is described here based upon literature.[7] One chain extension includes coupling-sulfurization and diafiltration to purify followed by detritylation and a second round of diafiltration. The synthesis conditions are shown in Scheme S1.

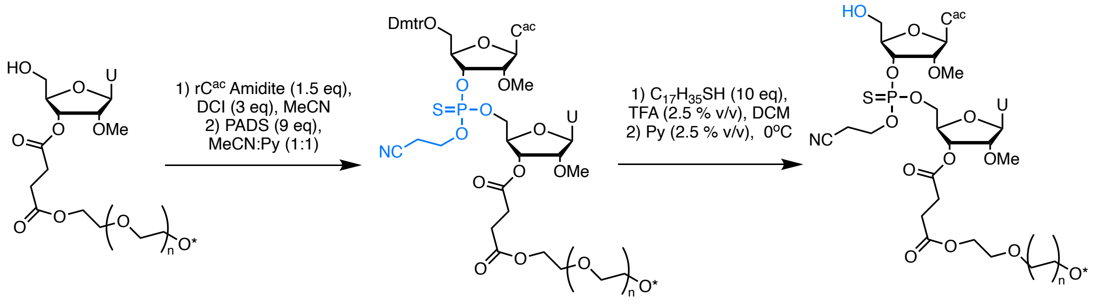

Scheme S1: Two-pot reaction procedure used to test ceramic membranes

5 kDa 4-arm PEG-mU-OH **1a** (0.25 g, 0.038 mmol) was co-evaporated with anhydrous acetonitrile (3 x 3 mL). 5'-O-Dmtr-2'-OMe-C^{ac} phosphoramidite (0.183 g, 0.228 mmol) was co-evaporated with anhydrous acetonitrile (3 x 2 mL) and dissolved in anhydrous acetonitrile (3 mL) and added to PEG-mU-OH. 3 Å molecular sieves (0.15 g) were added and left stirring for 10 mins to remove any residual water. DCI (0.054 g, 0.456 mmol) was added and left stirring for 60 mins. PADS (0.456 g, 1.52 mmol) and diluted with pyridine (3 mL) and left stirring for 60 mins. Reaction mixture poured into rig (200 mL total volume, 0.15% w/v) pressurised to 10 bar and left recirculating for 60 mins to reach steady state. Filtration was stopped after 8 diavolumes permeated ceramic membrane. Purified product washed out of rig with acetonitrile and solvent removed under reduced pressure to give dimer tritylated PEG-mUmC^{ac}-ODmtr as a light brown frothy glass (0.2 g, 83.3% filtration yield).

Purified PEG-mUmC^{ac}-ODmtr co-evaporated with anhydrous acetonitrile (3 x 3 mL) and dissolved in anhydrous DCM (2.5 mL), $C_{12}H_{25}SH$ (0.12 mL, 1.0 mmol) and TFA (0.063 mL, 0.85 mmol) were added to reaction mixture dropwise at 0 °C and left stirring for 60 mins. Colour went red-orange which dissipated over 20 mins. Pyridine (0.69 mL, 0.85 mmol) was added dropwise at 0 °C and left stirring for 10 mins to neutralize solution before filtration. Reaction mixture poured into rig (190 mL total volume, 0.15% w/v) pressurised to 10 bar and left recirculating for 60 mins to reach steady state. Filtration was stopped after 6 diavolumes

permeated ceramic membrane. Purified product washed out of rig with acetonitrile and solvent removed under reduced pressure to give dimer product PEG-mUmC^{ac}-OH as brown frothy glass (0.16 g, 96.6% filtration yield). ^{31}P NMR (162 MHz, $CDCl_3$) δ = 67.05 (0.40P), 66.62 (0.60P).

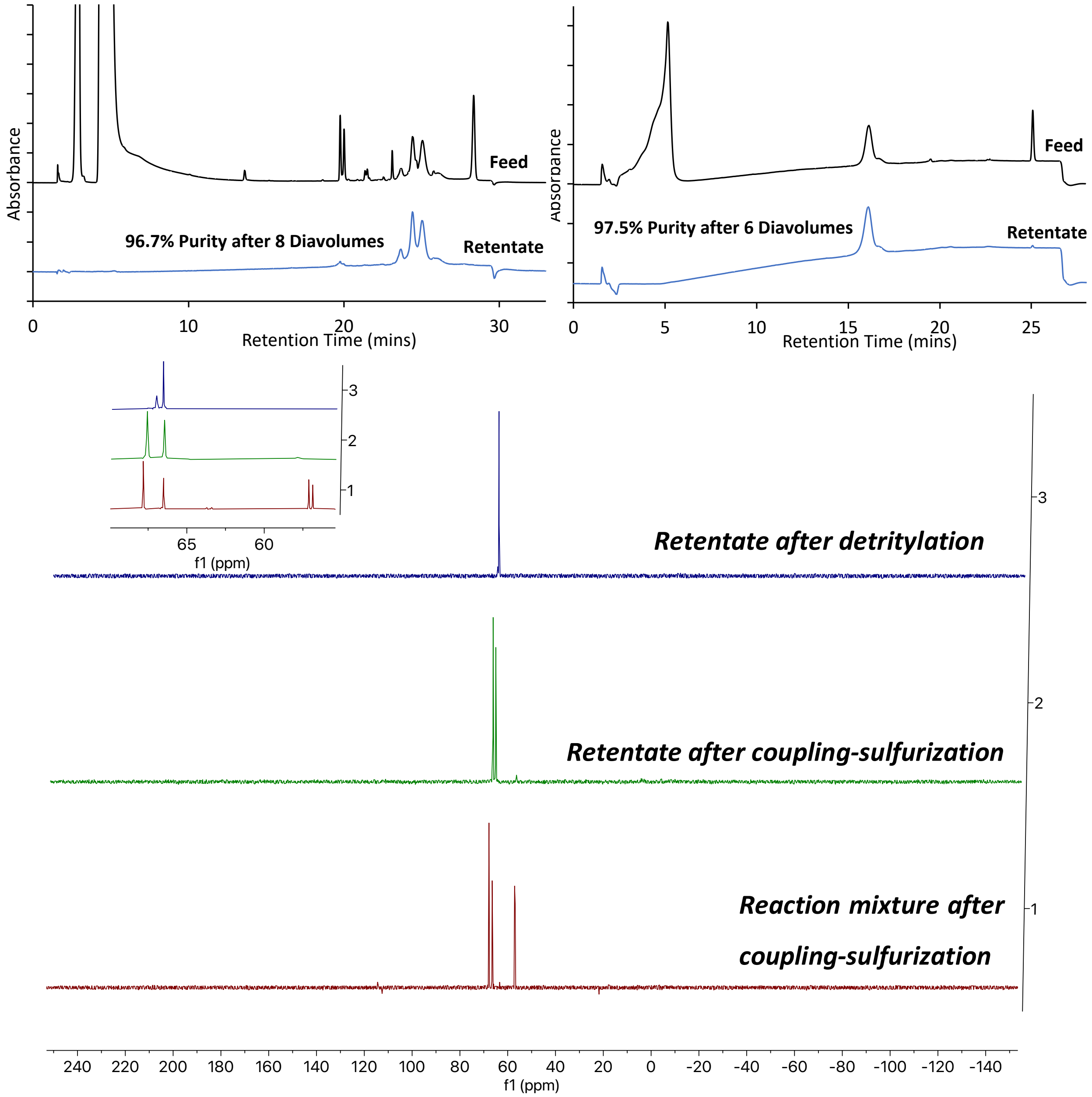

Figure S5: HPLC traces from two-pot experiment before and after filtration. (Top Left) HPLC trace for feed after coupling-sulfurization reaction on top and final retentate on bottom after 8 diavolumes. (Top Right) HPLC trace for feed after detritylation reaction on top and final retentate on bottom after 6 diavolumes. (Bottom) ^{31}P NMR spectra of reaction after coupling sulfurization showing phosphorothioate product and monothioate amidite by-product in red. Retentate after diafiltration

with remaining trace amidite at 58 ppm in green. Retentate after detritylation showing only purified phosphorothioate product.

6.1 Comments on Literature Two-Pot Synthesis

The two-pot protocol based upon literature was applied to the NF 750 Da membrane for the synthesis of a dimer (PEG-mUmC^{ac}-OH) oligonucleotide on a 4-arm 5 kDa PEG support.[7] The same reagents were used in OP-LPOS and the two-pot experiment, except that PADS sulfurization was carried out by adding 'fresh' PADS diluted with pyridine.

The only amidite by-product present in the post reaction mixture was monotioate amidite, identified by ^{31}P NMR at 58 ppm (Figure S5). After coupling-sulfurization reaction the membrane could remove all impurities in 8 diavolumes. This was surprising, as the membrane showed close to 80% amidite rejection during membrane screening. Monothioate amidite rejection was estimated from to be 53%, much lower than the fully protected amidites. Due to the reasonably high rejection of this species, some traces amounts remained and were visible by ^{31}P NMR and HPLC.

The dimer PEG-mUmC^{ac}-ODmtr generated in this step was then detritylated and purified by filtration in 6 diavolumes to remove all impurities, including the remaining trace amidites from the previous step. The overall filtration yield was 80% after 14 diavolumes across the two steps. The rejection of Dmtr-thiol scavenger peak was just 11% for this step, much lower than Kim *et al.* who found that the Dmtr-pyrrole scavenger showed >90% rejection and required an additional precipitation step to remove in their nanostar sieving approach.[8]

Rejection of the PEG-oligo was high for both steps, however unexpectedly the rejection was highest for the detritylated dimer at 99.4% compared to 97.8 % for tritylated dimer, where product mass is largest due to Dmtr group. Also, after detritylation the PEG-oligo-OH product will have more hydrophilic character as hydrophobic Dmtr is removed. Therefore, based upon solution-diffusion model of NF membrane transport, it was expected that rejection of PEG-oligo-OH will be lower than PEG-oligo-ODmtr as hydrophilic product will have more affinity for hydrophilic membrane. The higher rejection of the smaller and more hydrophilic PEG-oligo-OH may be explained by possible H-bonding interactions between deprotected products, which form higher order structures/clusters with larger hydrodynamic radius

providing higher membrane rejection. Dmtr protection may disrupt these interactions and lead to lower-than-expected rejection. A similar observation has been seen by Ormerod et al. where smaller Pd catalysts formed clusters, exhibiting higher membrane rejection compared to their larger molecular weight functionalised analogues.[9]

7 By-Products After Sulfurization and Amidite Quench

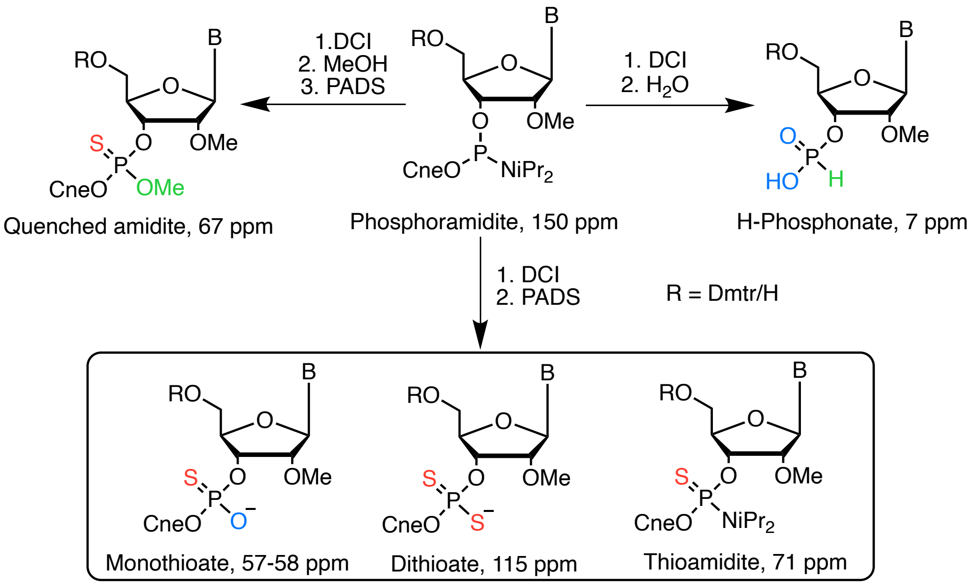

Figure S6: Formation of phosphoramidite by-products by quenching amidite with MeOH after coupling, hydrolysis to H-phosphonate in presence of DCI and various sulphur related by-products formed after sulfurization indicated in black box. ^{31}P NMR shifts included for all species.

8 HPLC Monitoring of Detritylation Step

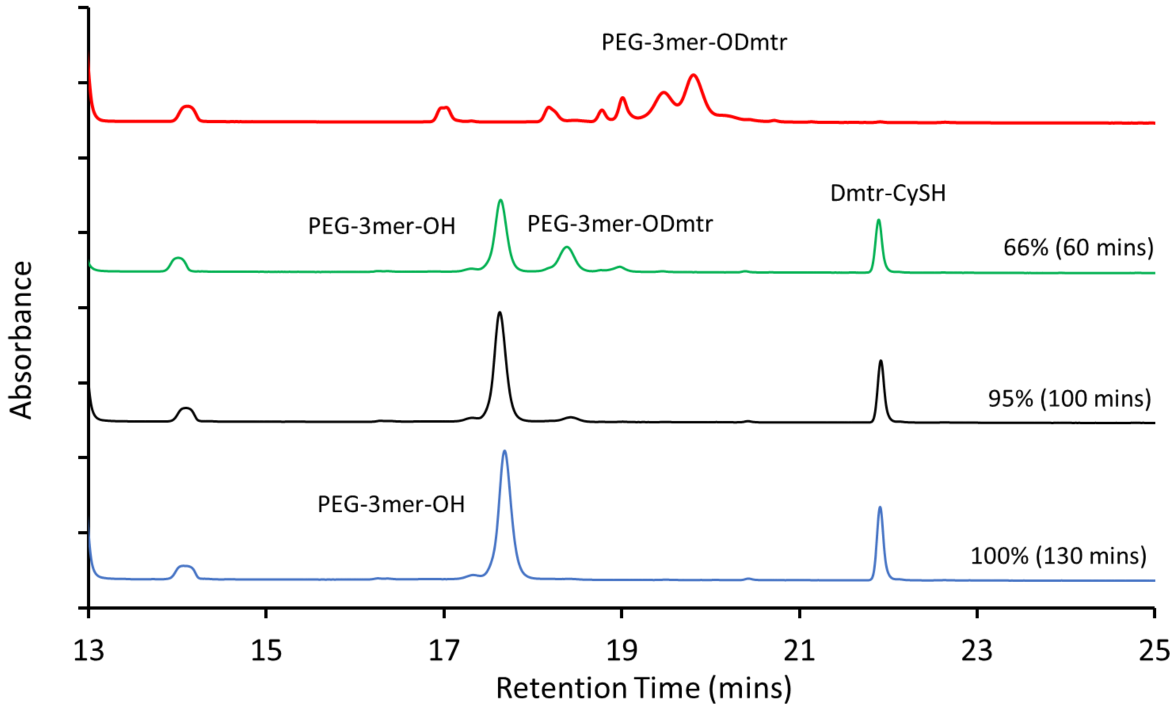

*Figure S7: HPLC progress of PEG-trimer detritylation reaction during 18mer (**3t**) synthesis. CySH (5 eq per 5'-ODmtr) and TFA (30 eq per 5'-ODmtr) added. Extra peaks around 18.5-19.5 mins have UV spectra for Dmtr protected species. Reaction yield was estimated based on LC relative area % of relevant peaks.*

9 Example ^{31}P NMR Before and After Diafiltration

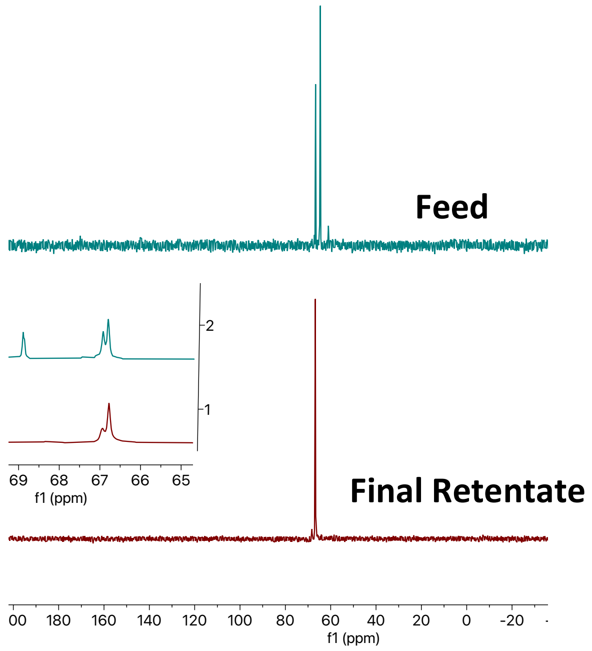

*Figure S8: ^{31}P NMR spectra showing the PEG-dimer **3d** and quenched amidite **6** phosphorothioate peaks in the feed before diafiltration and only the product peak remaining in final retentate after 5 diavolumes permeated.*

10 Filtration Data with NF 750 Da Membrane

Table S4: 6mer stepwise filtration yield data

Sequence	Yield Based on Rejection (%)	Mass Before Reaction (g)	Mass After Filtration (g)	Theoretical Yield (g)	Recovered Yield (%)[(a)]
**UU**	98.0	1.6	1.6	2.0	79.8
**UUA**	99.2	1.4	1.4	1.7	83.0
**UUAU**	99.2	1.2	1.2	1.4	86.6
**UUAUG**	99.2	1.1	1.0	1.2	81.4
**UUAUGC**	99.0	0.9	0.9	1.0	90.6

(a) Mass yield not accounting for the minor yield losses during sampling and transfer steps. Mass taken after extensive product drying under high vacuum when HPLC confirms high PEG-oligo purity (<97%) after diafiltration.

Table S5: 6mer stepwise filtration data

Sequence	Dmtr-CySH Rejection (%)	PEG-oligo Rejection (%)	Purity HPLC (%)[a]	Permeance ($Lm^{-2}h^{-1}$bar-1)	Diavolume
**UU**	34.9	99.8	99.5	2.2	10.0
**UUA**	35.1	100	99.1	2.1	8.0
**UUAU**	48.6	100	98.2	1.5	8.0
**UUAUG**	62.3	100	96.0	1.7	8.0
**UUAUGCA**[b]	76.1	100	97.2	1.6	10.0

(a) The purity was determined based on the relative area % by LC-DAD. (b) Diafiltration solvent swapped to MeCN:MeOH (9:1 v/v) for final two diavolumes which increased permeance and decreased Dmtr-CySH rejection.

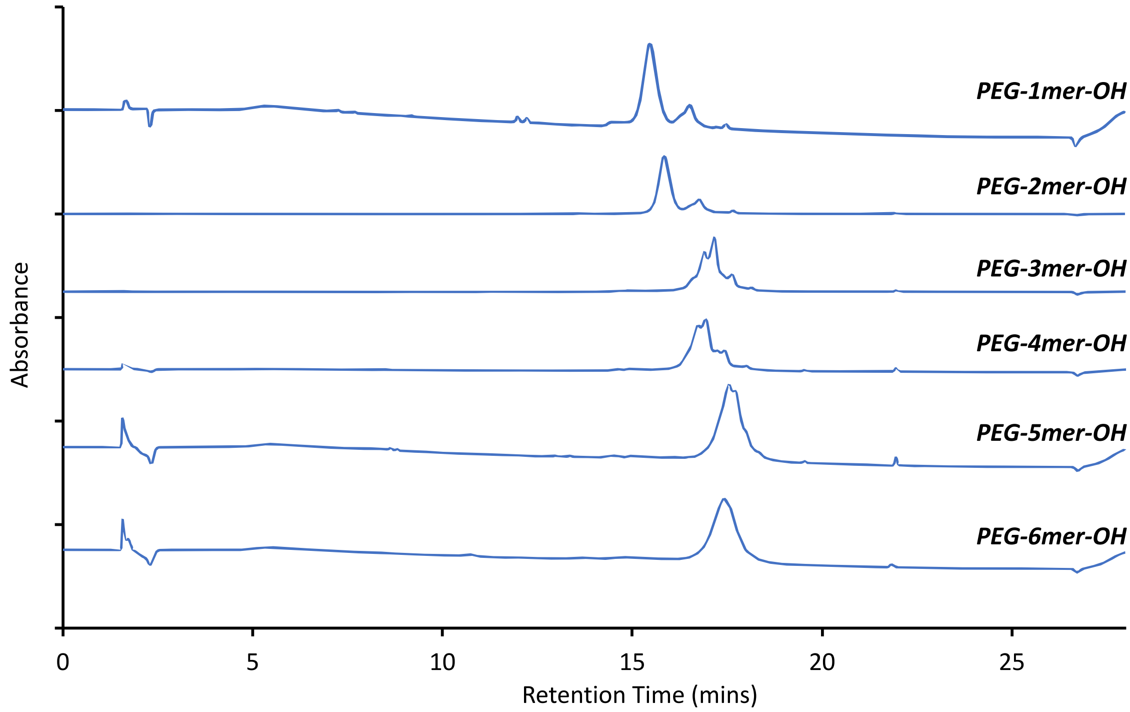

Figure S9: HPLC traces of final retentates after diafiltration during 6mer synthesis

11 Filtration Data with UF 2000 Da Membrane

Table S6: 18mer filtration stepwise yield data.

Sequence	Yield Based on Rejection (%)	Mass Before Reaction (g)	Mass After Filtration (g)	Theoretical Yield (g)	Recovered Yield (%)
**UU**	97.5	3.00	3.03	3.21	94.3
**UUA**	98.2	3.00	2.90	3.26	89.1[(a)]
**UUAU**	99.0	2.90	2.91	3.07	94.5
**UUAUG**	99.4	2.90	2.92	3.11	93.9
**UUAUGC**	100.0	2.92	2.98	3.09	96.3
**UUAUGCC**	100.0	2.70	2.61	2.85	91.6
**UUAUGCCU**	100.0	2.60	2.60	2.72	95.5
**UUAUGCCUA**	100.0	2.59	2.57	2.74	93.5
**UUAUGCCUAG**	100.0	2.28	2.28	2.40	94.8
**UUAUGCCUAGU**	100.0	2.10	2.12	2.18	97.0
**UUAUGCCUAGUC**	100.0	1.80	1.73	1.88	92.2
**UUAUGCCUAGUCG**	100.0	1.50	1.49	1.57	94.9
**UUAUGCCUAGUCGA**	100.0	1.49	1.39	1.56	89.4
**UUAUGCCUAGUCGAC**	100.0	1.38	1.38	1.43	96.3
**UUAUGCCUAGUCGACG**	100.0	1.34	1.13	1.40	81.1[(a)]
**UUAUGCCUAGUCGACGC**	100.0	1.00	1.00	1.03	97.0
**UUAUGCCUAGUCGACGCA**	100.0	1.00	1.03	1.04	99.2

(a) Lower yield due to product loss from handling error during solvent removal step.

Table S7: 18mer stepwise filtration data

Sequence	Dmtr-CySH Rejection (%)	PEG-oligo Rejection (%)	Purity HPLC (%)[(a)]	Permeance ($Lm^{-2}h^{-1}$bar-1)	Diavolume
**UU**	-16.8	99.5	98.0	20.2	5.0
**UUA**	-13.2	99.6	98.4	19.2	5.0
**UUAU**	16.0	99.8	98.5	15.8	5.0
**UUAUG**	23.8	99.9	98	14.0	5.0
**UUAUGC**	17.8	100.0	98.1	13.2	5.0
**UUAUGCC**	15.2	100.0	98.8	20.2	5.1
**UUAUGCCU**	29.8	100.0	98.7	19.2	5.0
**UUAUGCCUA**	57.7	100.0	97.7	15.8	5.0
**UUAUGCCUAG**	76.3[(b)]	100.0	91.3	10.2[(c)]	6.0
**UUAUGCCUAGU** [(d)]	72.8	100.0	91.4	8.8	5.1
**UUAUGCCUAGU**[(e)]	31.8	100.0	96.9	11.5	5.1
**UUAUGCCUAGUCG**[(f)]	50.7	100.0	96.6	6.2	5.0
**UUAUGCCUAGUCGA**	50.2	100.0	98.4	13.5	5.0
**UUAUGCCUAGUCGAC**	63.1	100.0	98.7	13.2	5.0
**UUAUGCCUAGUCGACG**	63.1	100.0	97.8	13.5	5.0
**UUAUGCCUAGUCGACGC**[(g)]	63.5	100.0	97.8	10.7	5.0
**UUAUGCCUAGUCGACGCA**[(g)]	65.7	100.0	96.5	9.8	5.0

(a) The purity was determined based on the relative area % by LC-DAD. (b) Dmtr-CySH rejection increased to 92% which was reduced by MeCN:MeOH (9:1 v/v) solvent system. (c) Higher permeance due to use of mixed solvent system; MeCN:MeOH (9:1 v/v). (d) Membrane replaced. (e) Filtration concentration reduced to 0.5% w/v and used MeCN:MeOH (9:1 v/v) solvent system. (f) Swapped diafiltration solvent to MeCN:MeOH (4:1 v/v) and maintained 0.5% w/v filtration conc. (g) Filtration concentration reduced to 0.3% w/v and maintained MeCN:MeOH (4:1 v/v) solvent system.

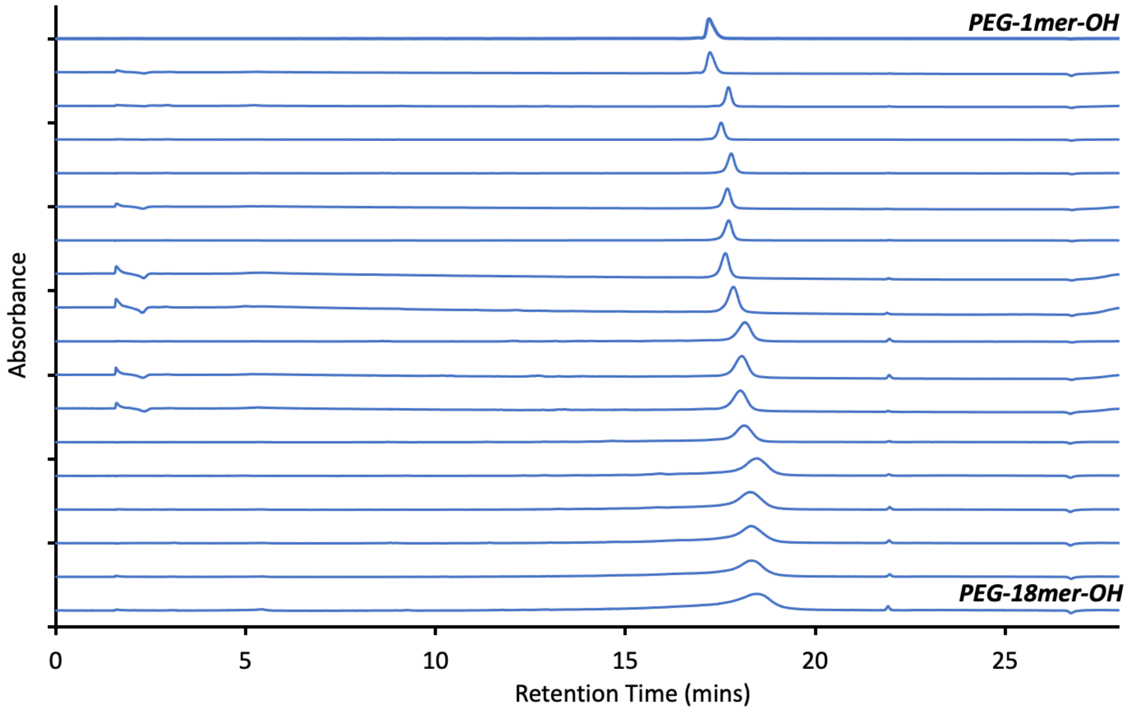

Figure S10: HPLC traces of final retentates after diafiltration during 18mer synthesis with starting PEG-1mer-OH and final PEG-18mer-OH indicated. Product peak becomes broader as chain length increases.

12 Transmembrane Pressure Excursion Study

To select a transmembrane pressure and observe the impact of lower cross-flow velocities for synthesis with UF 2000 Da membrane an excursion study was carried out using the conditions summarised in Table S8. 20 kDa 4-arm PEG-mU-OH **12a** was dissolved in acetonitrile (2 g/L) was added to membrane separation rig and left to reach steady state in recycle mode for 60 mins. Samples were taken at each pressure to record permeance and solute rejection (Figure S11). The system was left to reach steady state after each pressure change. Results show that under CFV = 0.5 m/s and after TMP = 5 bar that permeance plateaus meaning that this region is pressure independent (also called gel-layer control region). Increasing pressure after this point does not increase permeance but instead will consolidate gel-layer further. A CFV or 1 m/s and pressure of 5 bar was selected for filtration based upon these results. It should be noted that 1 m/s was the maximum achievable value using the equipment available and further increasing would be beneficial for membrane performance.

After solute washout the pure acetonitrile permeance was lower than for pristine membrane before study.

Table S8: Filtration conditions from TMP excursion study

Conditions		Units
Feed Flow	70	ml/min
Recirculation Flow	2.3/1.15	L/min
CFV	1/0.5	m/s
Conc.	2	g/L
Solute	20 kDa 4-arm PEG-mU-OH, **12a**	
Membrane SA	0.0049	m^2
Solute Rejection (%)	99.5%	

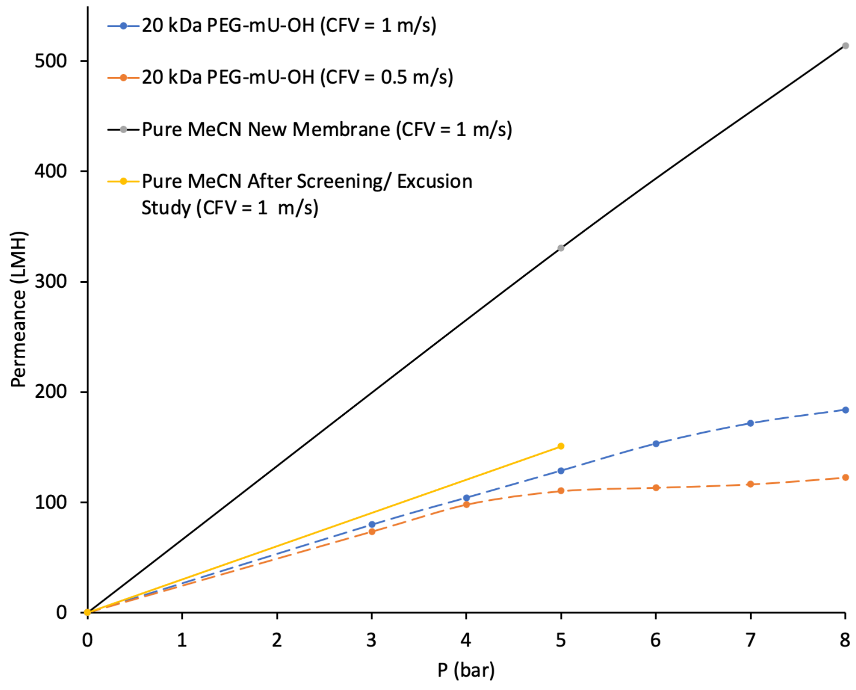

Figure S11: Transmembrane pressure excursion study using 20 kDa loaded PEG and UF 2000 Da membrane

13 Methanol Distillation Solvent Swap (DynoChem Simulation)

The addition of methanol to acetonitrile in the diafiltration solvent helped to improve the solubility of the PEG-oligo during diafiltrations. Diafiltrations proceeded using neat acetonitrile until the 10mer (**3l**) stage using the 4-arm 20 kDa PEG support and UF 2000 Da

membrane for example. Beyond this point there was a noted decrease in membrane performance in terms of permeance from 25 to 20 L m^{-2} h^{-1} bar^{-1} and Dmtr-CySH **7** rejection which increased from -16.8% to a maximum of 93.5%. It should be noted solubility was maintained at 1.8-22.3% during the reaction steps so reduction in performance was related to the membrane interface.

To address this, acetonitrile/methanol mixed solvent diafiltrations were conducted to enable extension to an 18mer (**3t**). If methanol is not removed after diafiltration or replaced with an alternative solvent then methanol will quench phosphoramidites in subsequent chain extension cycles. After each chain extensions in this study the retentate is evaporated to dryness which easily achieved at lab-scale but would be a challenging step during large-scale manufacturing.

However, batch azeotropic distillation is commonplace in the synthesis of pharmaceutical products. For the acetonitrile/methanol system a low boiling azeotrope of is found (Figure S12), which can be exploited in a batch distillation solvent swap to remove methanol post diafiltration (Figure S13). It should be noted that this will require more energy, solvent and cycle time compared to replacing methanol or optimising the membrane parameters or other feed characteristics to avoid adding a stronger solvent at all.

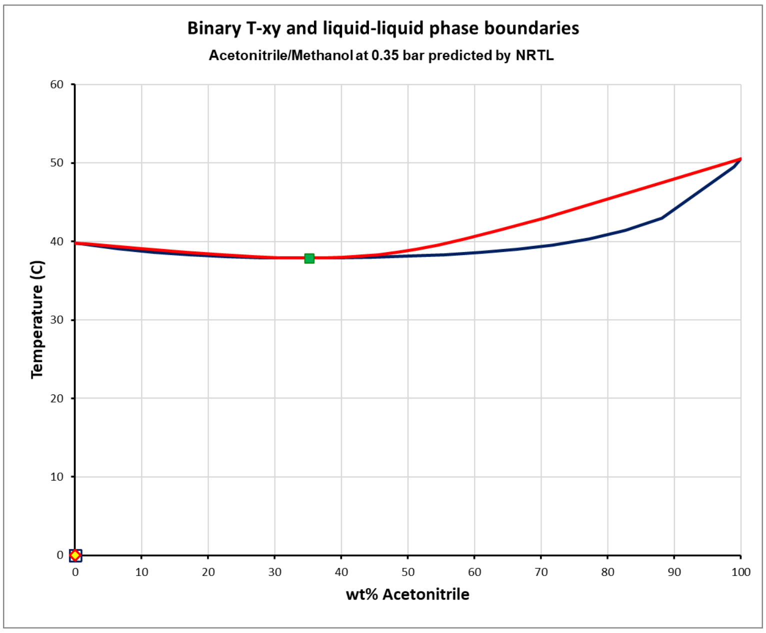

Figure S12: T-x-y diagram of MeCN/MeOH binary azeotrope composition at 0.35 bar

Under a moderate vacuum at pressure of 0.35 bar, the acetonitrile/methanol azeotrope occurs at 37.88 ^{o}C. This means that excessive hold temperatures are not needed and the azeotrope which contains predominantly methanol (64.85% at 0.35 bar). Distillation will remove methanol and acetonitrile at this favourable ratio, depleting the methanol mol fraction preferentially, with fresh acetonitrile addition when a minimum operating volume is reached. Since some small amounts of water will be present, a second binary azeotrope for acetonitrile/water will form at 47.6 ^{o}C containing mostly acetonitrile (12.2% water at 0.35 bar) which will be very powerful in removing any residual water, a key consideration for oligonucleotide synthesis where highly anhydrous conditions are required during synthesis (typically less than 50 ppm). Azeotropic based distillation of acetonitrile/methanol/water mixtures using an entrainer for ternary extraction distillation has been studied in the literature but no examples were found specifically on acetonitrile/methanol distillation or solvent swaps in pharmaceutical applications.[10] Recently, a two-column pressure-swing distillation for purifying oligonucleotide solid-phase waste streams has been proposed, where a pure acetonitrile stream at high pressure with very low water content.[11]

A simulation of an exemplar large-scale solvent swap was conducted within the Dyonchem^{TM} software packages solvent swap function, assuming binary azeotrope behaviour throughout. This will have to be experimentally verified given the high concentration of the product at the end of the concentration prior to commencement of the next reaction step where the product, although non-volatile, may alter the characteristic or the solution and position of the azeotrope. In the example simulation shown here a volume reduction, then fed batch constant volume distillation of MeCN/MeOH (9:1 v/v, 500 L) to 100% MeCN (50 L), using a maximum jacket temperature of 60 ^{o}C.[12] The model assumes that 10 kg of PEG-oligo at the 10mer stage (**3l**) was present, giving a 5% w/v filtration concentration. This was distilled down to 100 L or 10% w/v for chain extension to take place at a higher concentration in the membrane rig or a separate batch reactor in the next cycle.

Results from this simple simulation indicate that methanol volume can be reduced from 50 L to 6.8 µL after 39 hours and 202.6 L of fresh solvent addition. Rounding this up gives 0.01 mL methanol remaining, or approximately 0.001 equivalents of amidite, essentially negligible at

a 10 kg PEG-oligo scale where 6 eq (1.5 eq per arm, >1 kg amidite) amidite will be added in the next step. If only a volume reduction step was included, without fresh solvent addition, there would be 20 mL of methanol remaining, which is approximately 2 equivalents of amidite. Therefore, additional solvent addition is essential to achieve sufficient methanol removal. The additional solvent and energy consumption may be offset if it enables the use of a highly concentrated diafiltration step, assuming the cycle time and hot hold temperature is acceptable.

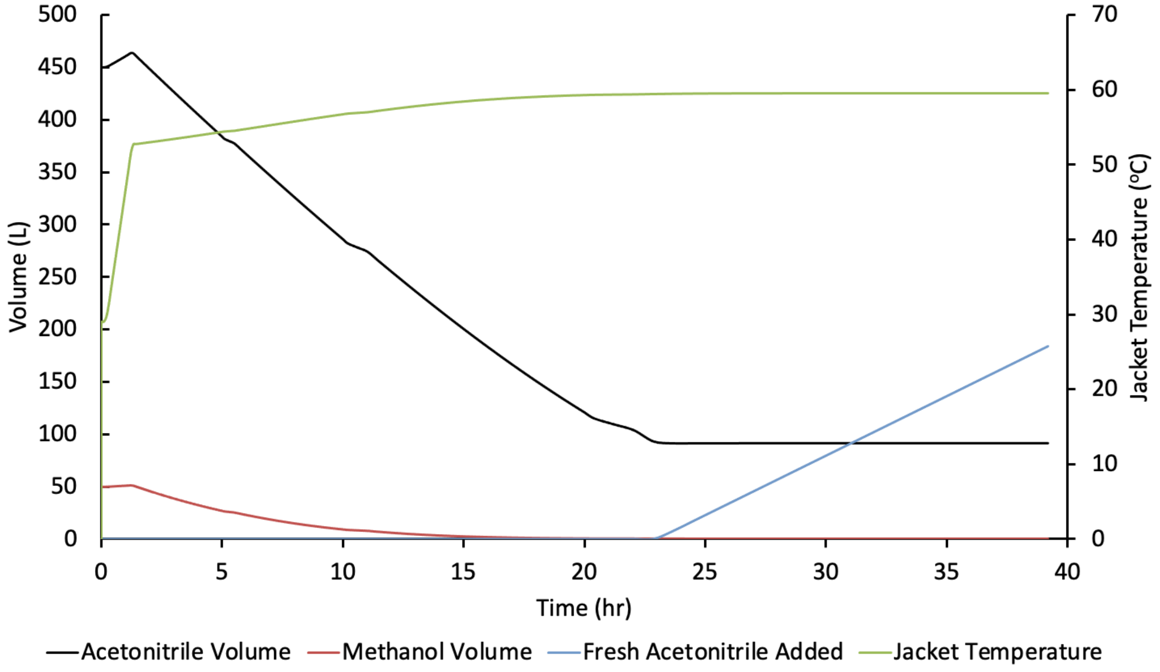

Figure S13: Decrease in volume of MeOH during batch distillation from 50L to 6.8 µL after 39 hours and 202.6 L of fresh solvent addition. Distillation at 0.35 bar to keep jacket temperature below 60 °C to avoid product decomposition.

However, given the reaction steps can be run at such high concentrations (up to around 20% w/v) and a large amount of volume in the batch reactor must be reserved for dilution to conduct membrane filtration (1-10% w/v), methanol addition to enable higher diafiltration concentrations throughout the synthetic cycles could result in a further increase in process intensity along with azeotropic distillation it out of the system. Given the relatively high product concentrations and the non-ideality of the binary distillation further experimentation will be required to verify that the product, reagents and impurities do not significantly shift the conditions in the distillation.

14 Dmtr-CySH Spiking Experiment and Solvent Screening

Based upon the observations during experiments at longer chain lengths several additional reaction screening experiments were performed to test the impact of Dmtr-CySH **7** impurity and explore alternative solvent mixtures for performing reactions and filtrations in a membrane reactor-separator. To determine if Dmtr-CySH **7** is a critical impurity which may affect subsequent chain extension steps a spiking study was carried out where 10 eq was spiked into a standard OP-LPOS reaction. The developed OP-LPOS reaction used DCM as this is the choice solvent for detritylation; however, for integration with a membrane reactor-separator it would be advantageous to use a single acetonitrile solvent system, or potentially to use a mixed solvent system, diluting with a stronger polar aprotic solvent (DMF or sulfolane) to improve oligonucleotide solubility at longer chain lengths. Five reactions were carried out, including a control (Table S9), to compare various reaction conditions using loaded 10 kDa 4-arm PEG-mU-OH **2c** (10% w/v reaction concentration), no further purification was carried out by CVD.

Reaction completion was determined by HPLC and ^{31}P NMR (Table S9). Coupling and sulfurization reactions proceeded to completion as measured by HPLC and ^{31}P NMR. The main difference between reaction conditions were observed during detritylation reaction where MeCN:DMF (3:1 v/v) did not lead to any detritylation and completion varied for the other conditions between 87-99% after 150 mins. MeCN:DCM (2:1 v/v) and MeCN:Sulfolane (3:1 v/v) were almost quantitively detritylated, whereas using neat acetonitrile and spiking experiment with Dmtr-CySH **7** appeared to slow the reaction. These results indicate that removal of Dmtr-CySH **7** and solvent choice is particularly important for detritylation completion. Future OP-LPOS reaction optimisation with MeCN:Sulfolane appears to be a promising apolar solvent mixture for future integration with a membrane reactor-separator as sulfolane has been proposed as a stronger solvent when oligonucleotide solubility becomes challenging.

Table S9: OP-LPOS impurity spike and polar aprotic solvent screening experiments.

**Entry[(a)]**	**Coupling conversion (%) at 1 hr[(b)]**	**Sulfurization conversion (%) at 1 hr[(c)]**	**Detritylation conversion (%) at 1/2.5 hr[(d)]**
MeCN:DCM (2:1 v/v)[(e)]	>99	>99	87/97
MeCN:DCM (2:1 v/v) + 10 eq Dmtr-CySH[(e)]	>99	>99	69/93
MeCN	>99	>99	84/87
MeCN:DMF (3:1 v/v)	>99	>99	2/2
MeCN:Sulfolane (3:1 v/v)	>99	>99	90/99

(a) Reagent equivalents per arm of loaded 10 kDa 4-arm PEG-mU-OH **2c**; (i) Coupling: 2'-OMe-mU-ODmtr phosphoramidite(1.5 eq), DCI (4 eq), 10% w/v 3Å molecular sieves; Amidite quench: MeOH (5 eq); (ii) Sulfurization: POS (3 eq); (iii) Detritylation: TFA (15 eq) and CySH (5 eq); Solvents during all reaction steps included in table. (b) Estimated based on LC relative area (%) conversion of PEG-mU-OH after coupling. (c) Estimated based on ^{31}P NMR relative area (%) conversion of phosphite triester peak after sulfurization. (d) Estimated based on LC relative area (%) conversion to PEG-mUmU-OH peak after acid treatment.

15 Acetonitrile/Sulfolane Mixed Solvent Screening During Membrane Filtration

To evaluate the performance of an acetonitrile/sulfolane solvent system for the potential use during the synthesis of full-length oligos an additional experiment was run using this solvent system while spiking in the PEG-18mer (**3t**) (0.45 g) and Dmtr-CySH **7** (0.045 g, 12 eq) previously synthesised. The same membrane used during the 18mer (**3t**) was used and 12 eq Dmtr-CySH **7** (4 eq from PEG-oligo, 6 eq from phosphoramidites, 2 eq further from incomplete removal during previous chain extensions) were employed to mimic a potential scenario

where the scavenged trityl protecting group is the slowest impurity to be removed and the membrane exhibits some fouling. A solvent composition of acetonitrile/sulfolane (3:1 v/v) was selected for test screening to match the composition used in the previous section for the dimer synthesis which showed the OP-LPOS worked well employing this solvent ratio.

Before membrane filtration of the test solutes was conducted the pure solvent composition of MeCN:sulfolane (3:1 v/v) showed a significantly lower permeance of 2.7 $Lm^{-2}h^{-1}bar^{-1}$ compared to 10.3 $Lm^{-2}h^{-1}bar^{-1}$ using MeCN:MeOH (4:1 v/v) after the 18mer (**3t**) run. The solutes were then added, and the same filtration conditions were attempted as the 18mer (**3t**) run which used a mixed solvent composition of MeCN:MeOH (4:1 v/v) and a filtration concentration of 0.3% w/v (Table S10). However, when the PEG-18mer and Dmtr-CySH **7** were added to the rig the in-line filter quickly clogged at this filtration concentration. The filter was replaced the concentration was reduced to 0.15% w/v by further dilution with MeCN:sulfolane (3:1 v/v). After reaching steady state permeance the PEG-18mer rejection remained at 100% while the Dmtr-CySH **7** rejection was higher at 77.9% compared to the original run using methanol as a co-solvent. Again, the permeance was also lower when using acetonitrile/sulfolane at 2.3 $Lm^{-2}h^{-1}bar^{-1}$. These preliminary results show a mixed acetonitrile/sulfolane solvent system may be useful in the synthesis of full-length therapeutic oligonucleotides if the exact solvent composition and membrane parameters can be optimised further to improve membrane performance as discussed previously.

Table S10: Filtration results from PEG-18mer and Dmtr-CySH filtration test screening with an acetonitrile/sulfolane mixed solvent system.

**Solvent System**	**Conc. (PEG-18mer % w/v)**	**Permeance ($Lm^{-2}h^{-1}bar^{-1}$)**	**PEG-18mer Rejection (%)**	**Dmtr-CySH Rejection (%)**
MeCN:MeOH (4:1 v/v)	0	10.3 after 18mer (**3t**) run.	n/a	n/a
MeCN:MeOH (4:1 v/v)	0.3	9.8 during 18mer (**3t**) run	100	65.7

MeCN:sulfolane (3:1 v/v)	0	2.7	n/a	n/a
MeCN:sulfolane (3:1 v/v)	0.3	Product precipitated in filter.	n/a	n/a
MeCN:sulfolane (3:1 v/v)	0.15	2.3	100	77.9

References

(1) Parga, C. Liquid Phase Membrane Supported Cyclical Flow Synthesis of Oligonucleotides for Therapeutic Applications, University College Dublin, 2021.

(2) Peeva, L.; Da Silva Burgal, J.; Heckenast, Z.; Brazy, F.; Cazenave, F.; Livingston, A. Continuous Consecutive Reactions with Inter-Reaction Solvent Exchange by Membrane Separation. Angew. Chem. **2016**, 128 (43), 13774–13777. https://doi.org/10.1002/ange.201607795.

(3) Deleavey, G. F.; Damha, M. J. Designing Chemically Modified Oligonucleotides for Targeted Gene Silencing. Chem. Biol. **2012**, 19 (8), 937–954. https://doi.org/10.1016/j.chembiol.2012.07.011.

(4) Creusen, G.; Akintayo, C. O.; Schumann, K.; Walther, A. Scalable One-Pot-Liquid-Phase Oligonucleotide Synthesis for Model Network Hydrogels. J. Am. Chem. Soc. **2020**, 142 (39), 16610–16621. https://doi.org/10.1021/jacs.0c05488.

(5) Marchetti, P.; Jimenez Solomon, M. F.; Szekely, G.; Livingston, A. G. Molecular Separation with Organic Solvent Nanofiltration: A Critical Review. Chem. Rev. **2014**. https://doi.org/10.1021/cr500006j.

(6) Vandezande, P.; Gevers, L. E. M.; Vankelecom, I. F. J. Solvent Resistant Nanofiltration: Separating on a Molecular Level. Chem. Soc. Rev. **2008**. https://doi.org/10.1039/b610848m.

(7) Kim, J. F.; Gaffney, P. R. J.; Valtcheva, I. B.; Williams, G.; Buswell, A. M.; Anson, M. S.; Livingston, A. G. Organic Solvent Nanofiltration (OSN): A New Technology Platform for Liquid-Phase Oligonucleotide Synthesis (LPOS). Org. Process Res. Dev. **2016**, 20 (8), 1439–1452. https://doi.org/10.1021/acs.oprd.6b00139.

(8) Kim, J. F.; Gaffney, P. R. J.; Valtcheva, I. B.; Williams, G.; Buswell, A. M.; Anson, M. S.; Livingston, A. G. Organic Solvent Nanofiltration (OSN): A New Technology Platform for Liquid-Phase Oligonucleotide Synthesis (LPOS). Org. Process Res. Dev. **2016**. https://doi.org/10.1021/acs.oprd.6b00139.

(9) Ormerod, D.; Dorbec, M.; Merkul, E.; Kaval, N.; Lefèvre, N.; Hostyn, S.; Eykens, L.; Lievens, J.; Sergeyev, S.; Maes, B. U. W. Synthesis of Pd Complexes Containing Tailed NHC Ligands and Their Use in a Semicontinuous Membrane-Assisted Suzuki Cross-Coupling Process. Org. Process Res. Dev. **2018**, 22 (11), 1509–1517. https://doi.org/10.1021/acs.oprd.8b00273.

(10) Wang, Y.; Bu, G.; Geng, X.; Zhu, Z.; Cui, P.; Liao, Z. Design Optimization and Operating Pressure Effects in the Separation of Acetonitrile/Methanol/Water Mixture by Ternary Extractive Distillation. J. Clean. Prod. **2019**, 218, 212–224. https://doi.org/10.1016/j.jclepro.2019.01.324.

(11) Meadhra, R. Ó.; Fleury, C.; Guelat, B.; Venturoni, F. Acetonitrile Regeneration from Oligonucleotide Production Waste Streams. Org. Process Res. Dev. **2024**. https://doi.org/10.1021/acs.oprd.4c00188.

(12) Solvent swap distillation - Dynochem Resources. https://dcresources.scale-up.com/?q=solvent+swap#q=solvent+swap___5380 (accessed 2024-09-11).

NMR Spectra

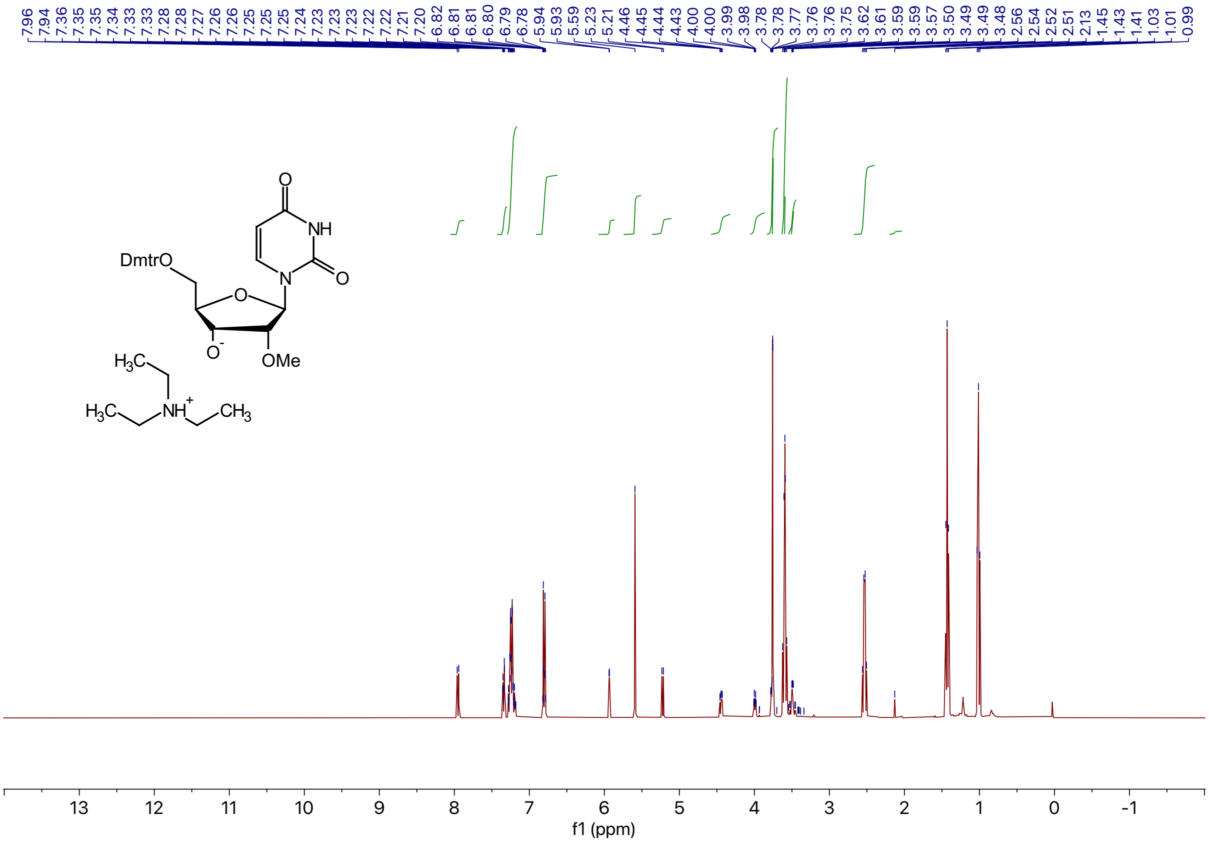

Figure S14: ^{1}H NMR spectra of 2'-O-methyl-5'-O-(4'',4'''-dimethoxytriphenylmethyl)uridine triethylammonium salt

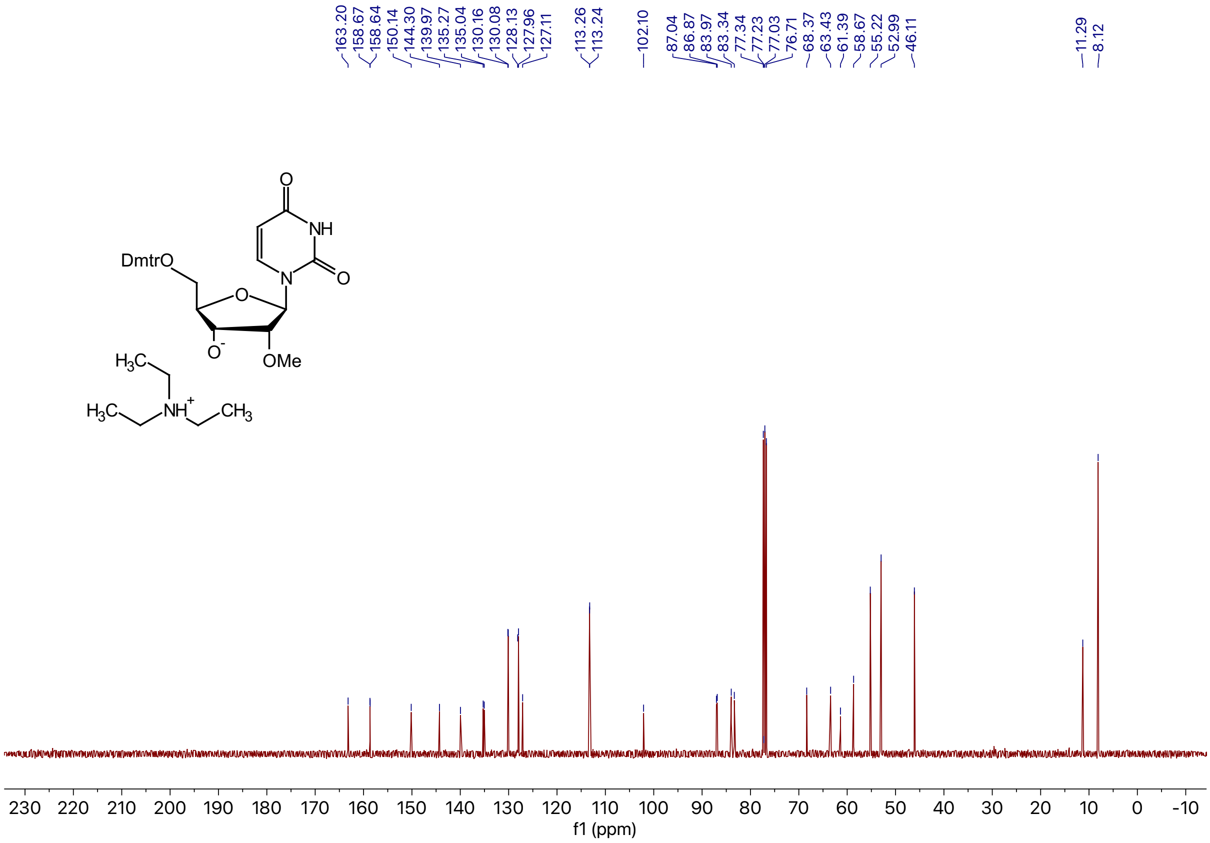

Figure S15: ^{13}C NMR spectra of 2'-O-methyl-5’-O-(4'',4'''-dimethoxytriphenylmethyl)uridine triethylammonium salt

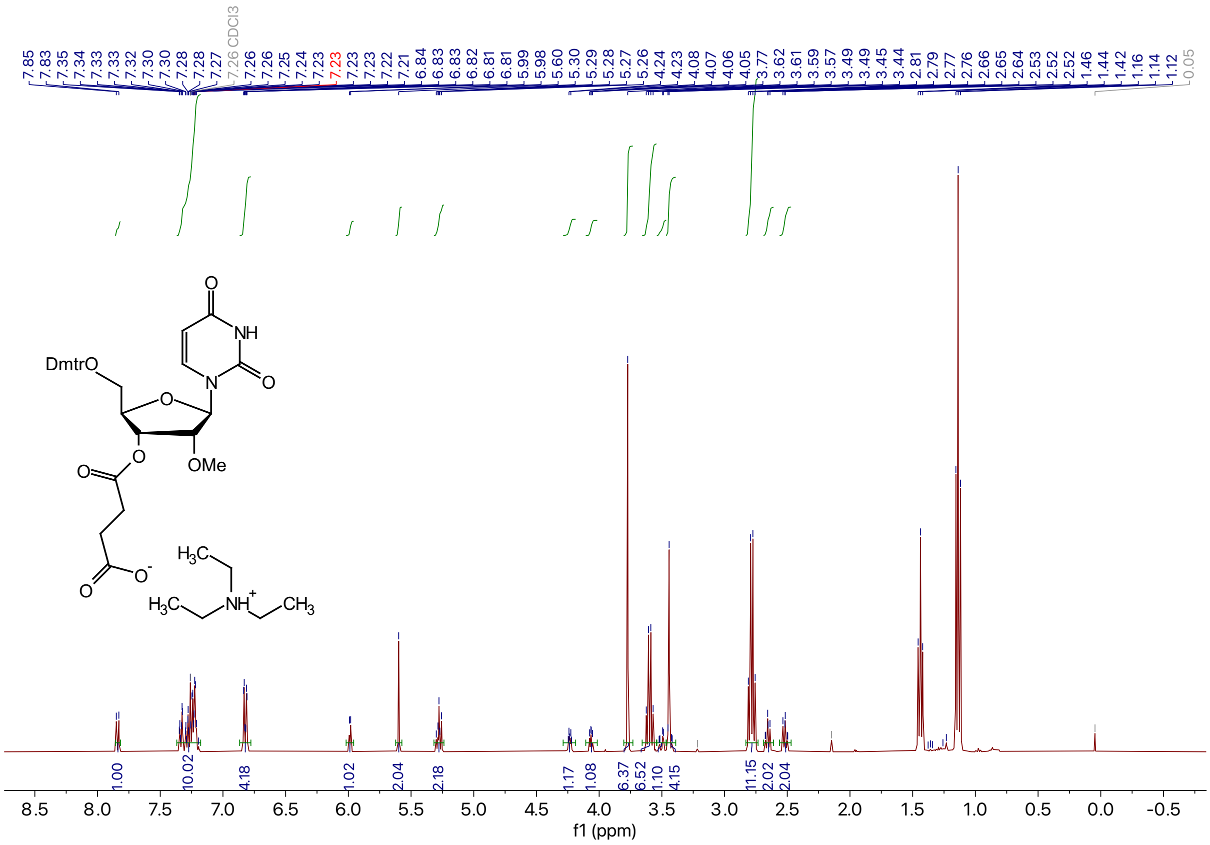

*Figure S16: ^{1}H NMR spectra of 2'-O-methyl-3'-O-succinyl-5'-O-(4'',4'''-dimethoxytriphenylmethyl)uridine triethylammonium salt, **5***

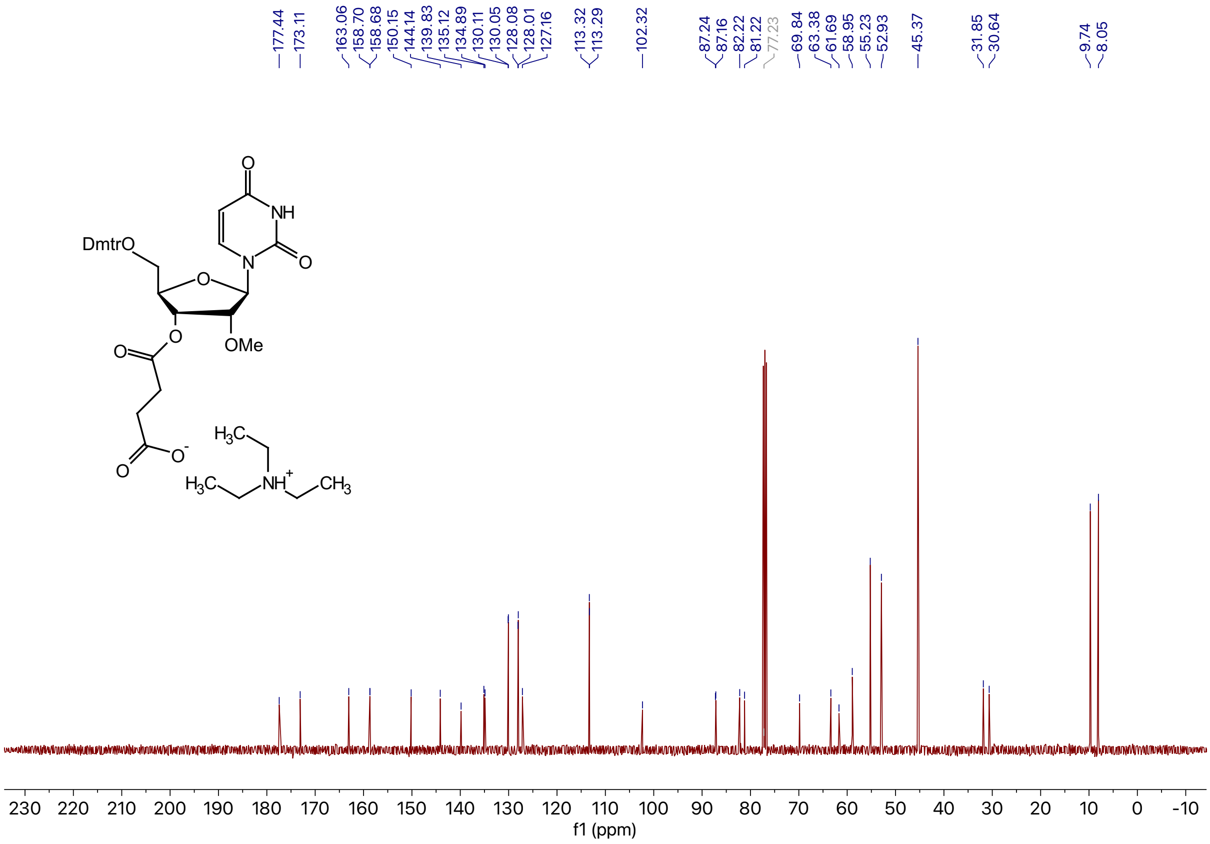

*Figure S17: ^{13}C NMR spectra of 2'-O-methyl-3'-O-succinyl-5'-O-(4'',4'''-dimethoxytriphenylmethyl)uridine triethylammonium salt, **5***

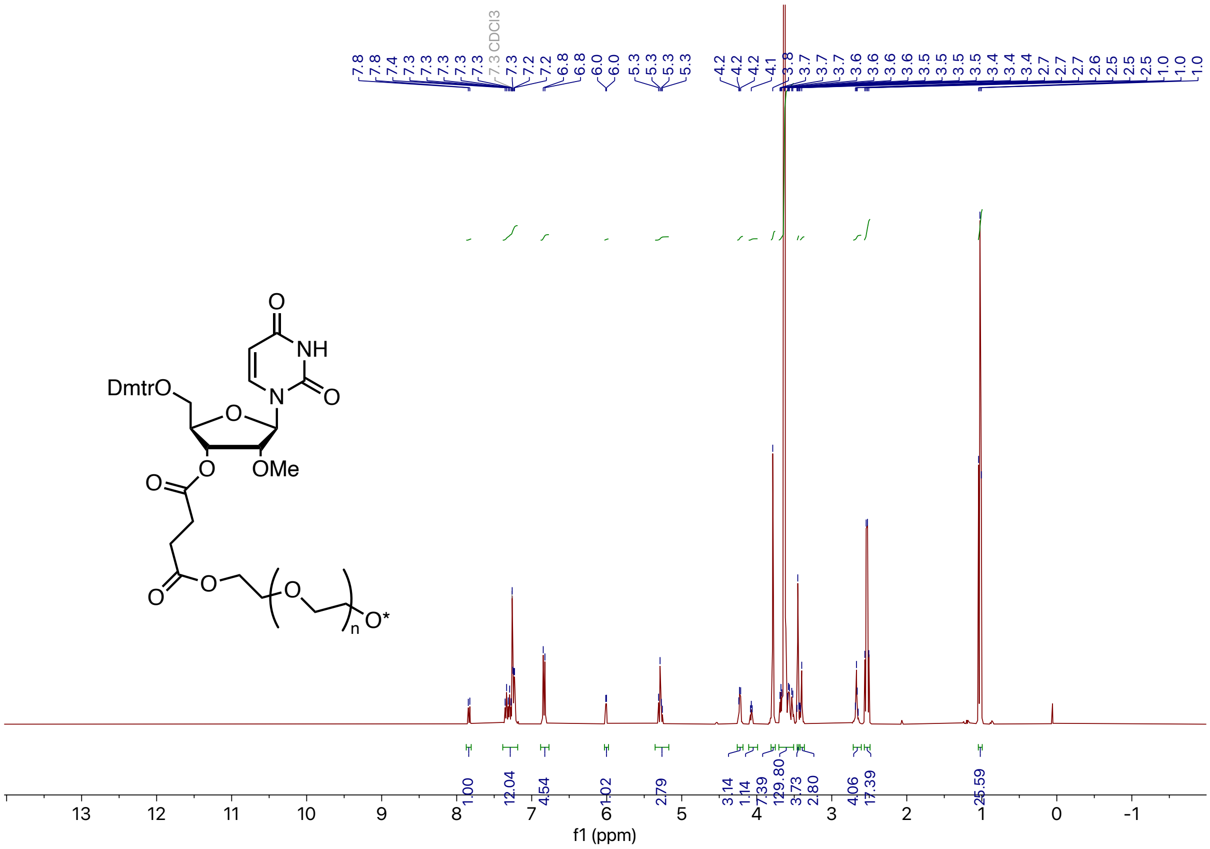

*Figure S18: ^{1}H NMR spectra of 5 kDa 4-arm PEG-O-mU-ODmtr, **1b***

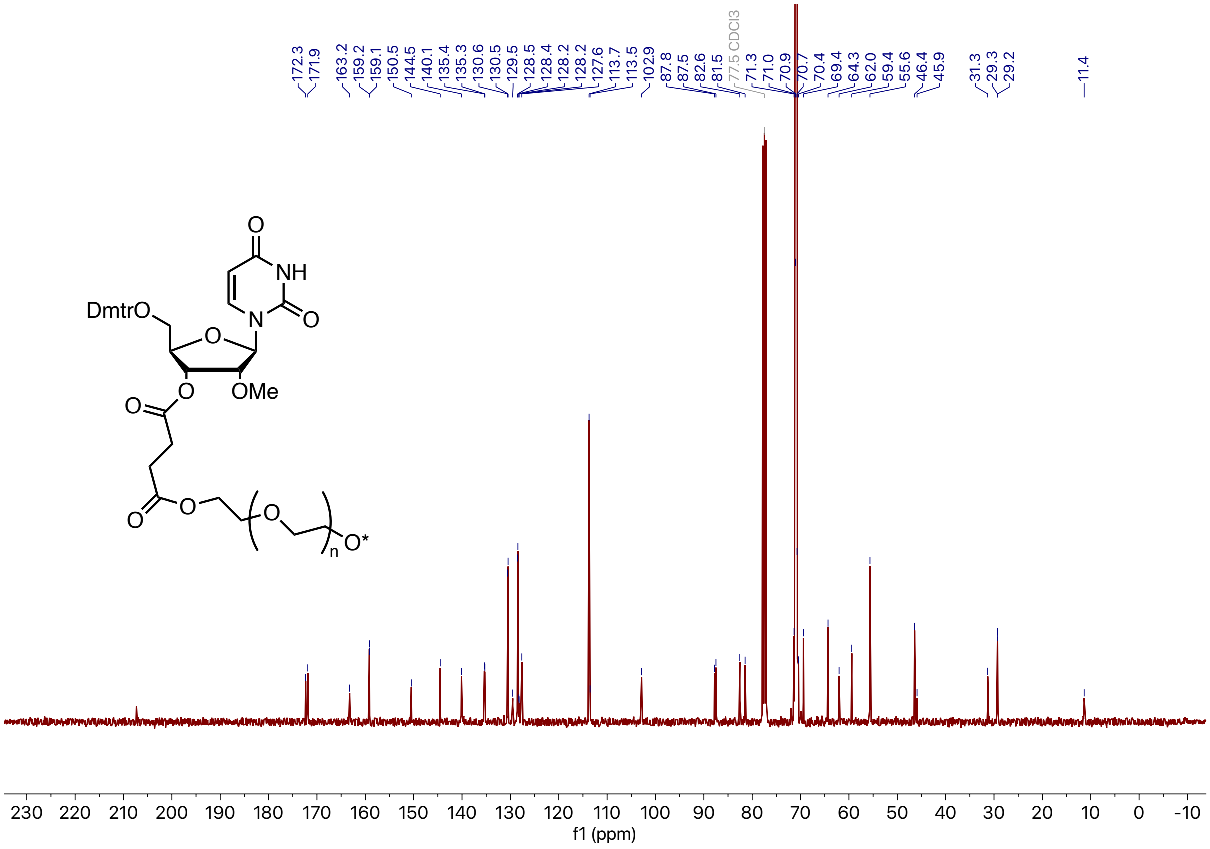

*Figure S19: ^{13}C NMR spectra of 5 kDa 4-arm PEG-O-mU-ODmtr, **1b***

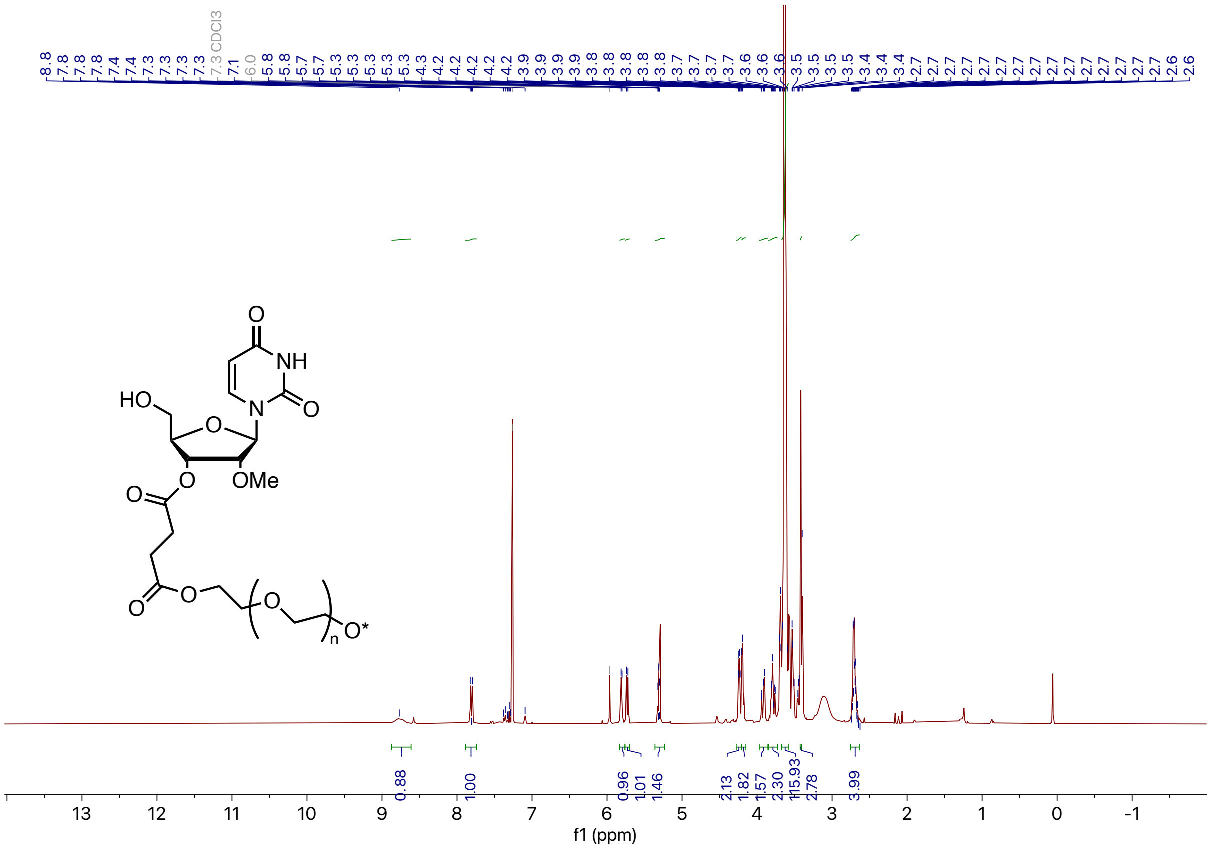

*Figure S20: ^{1}H NMR spectra of 5 kDa 4-arm PEG-O-mU-OH, **1c***

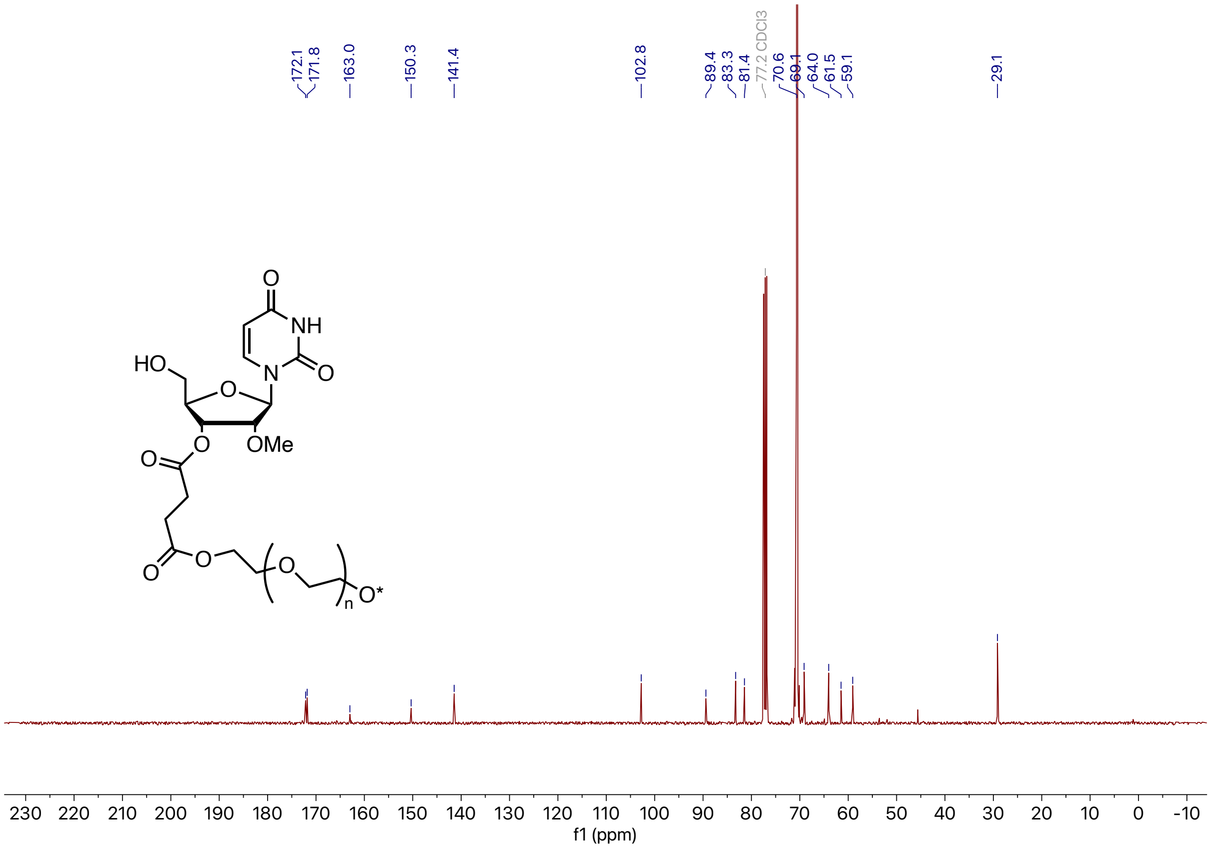

*Figure S21: ^{13}C NMR spectra of 5 kDa 4-arm PEG-O-mU-OH, **1c***

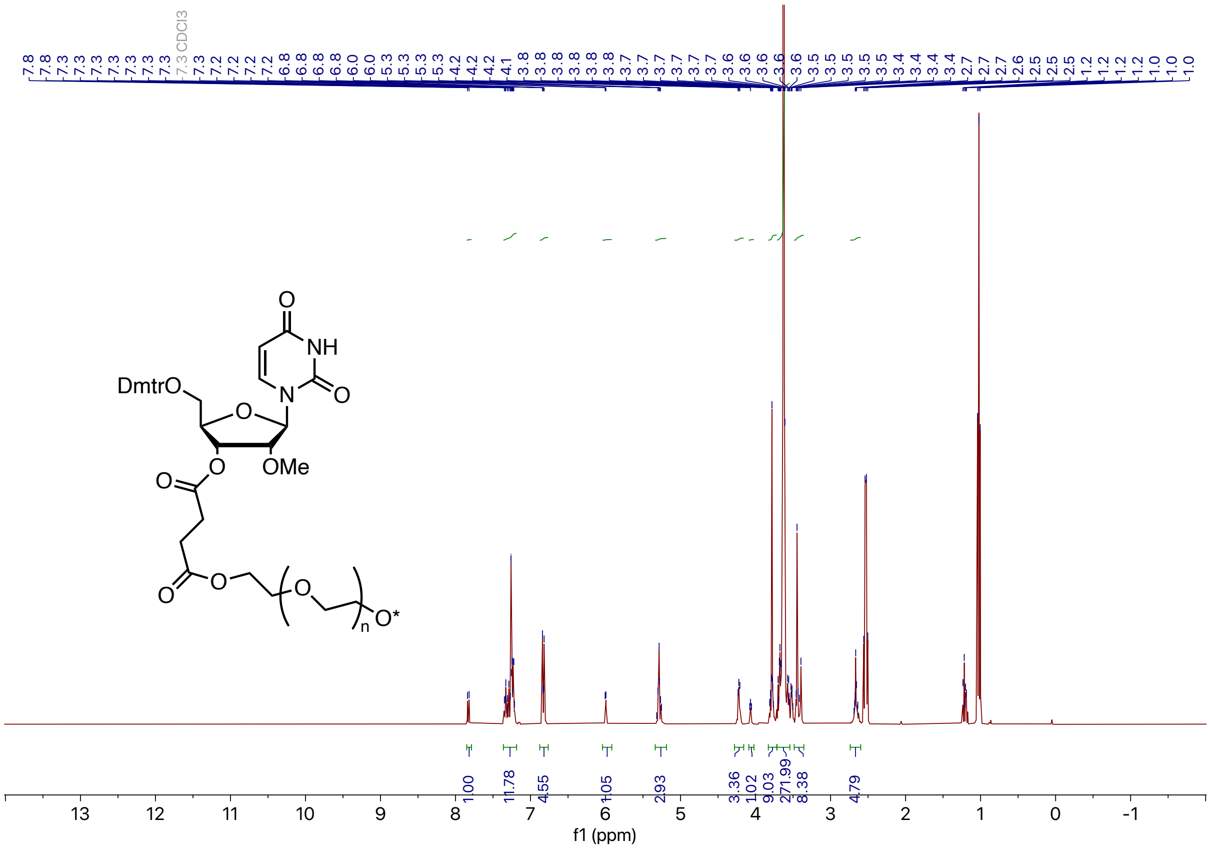

Figure S22: ^{1}H NMR spectra of 10 kDa 4-arm PEG-O-mU-ODmtr, ***2b***

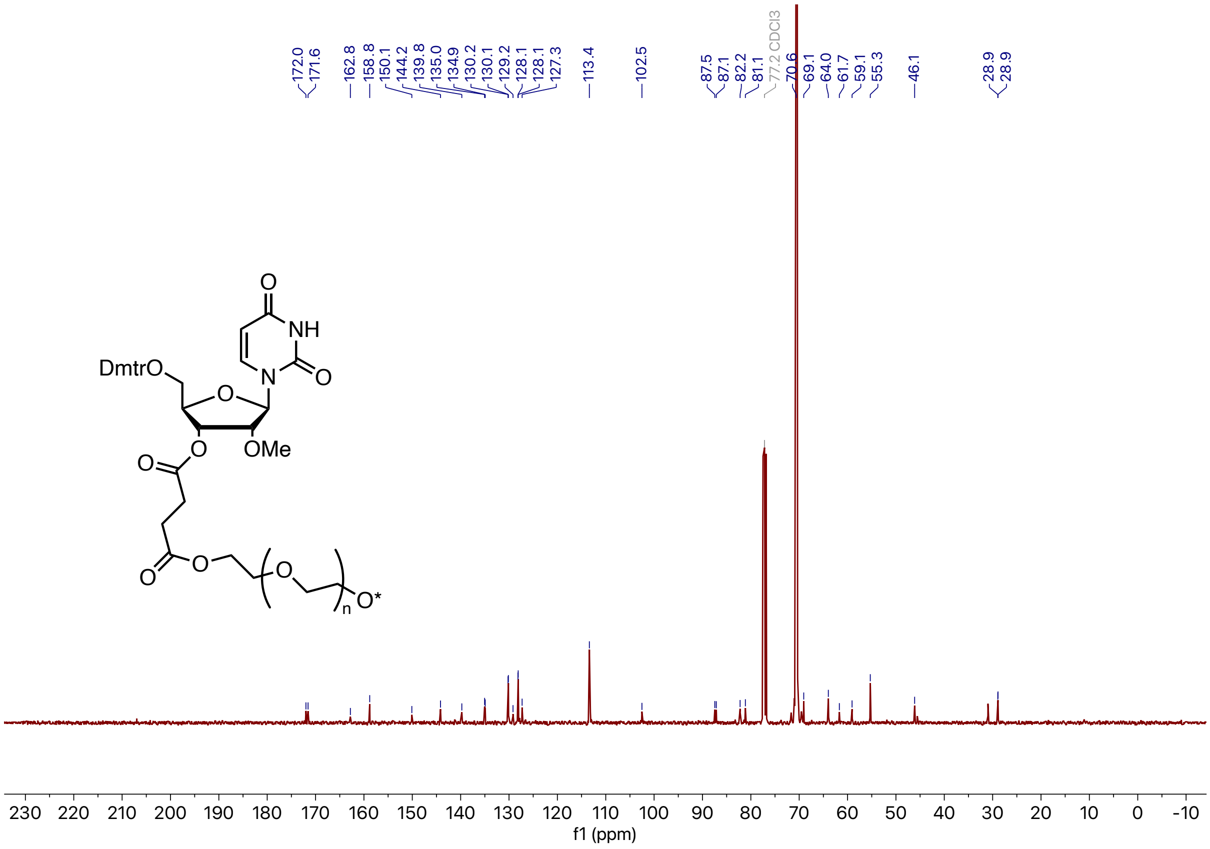

Figure S23: ^{13}C NMR spectra of 10 kDa 4-arm PEG-O-mU-ODmtr, ***2b***

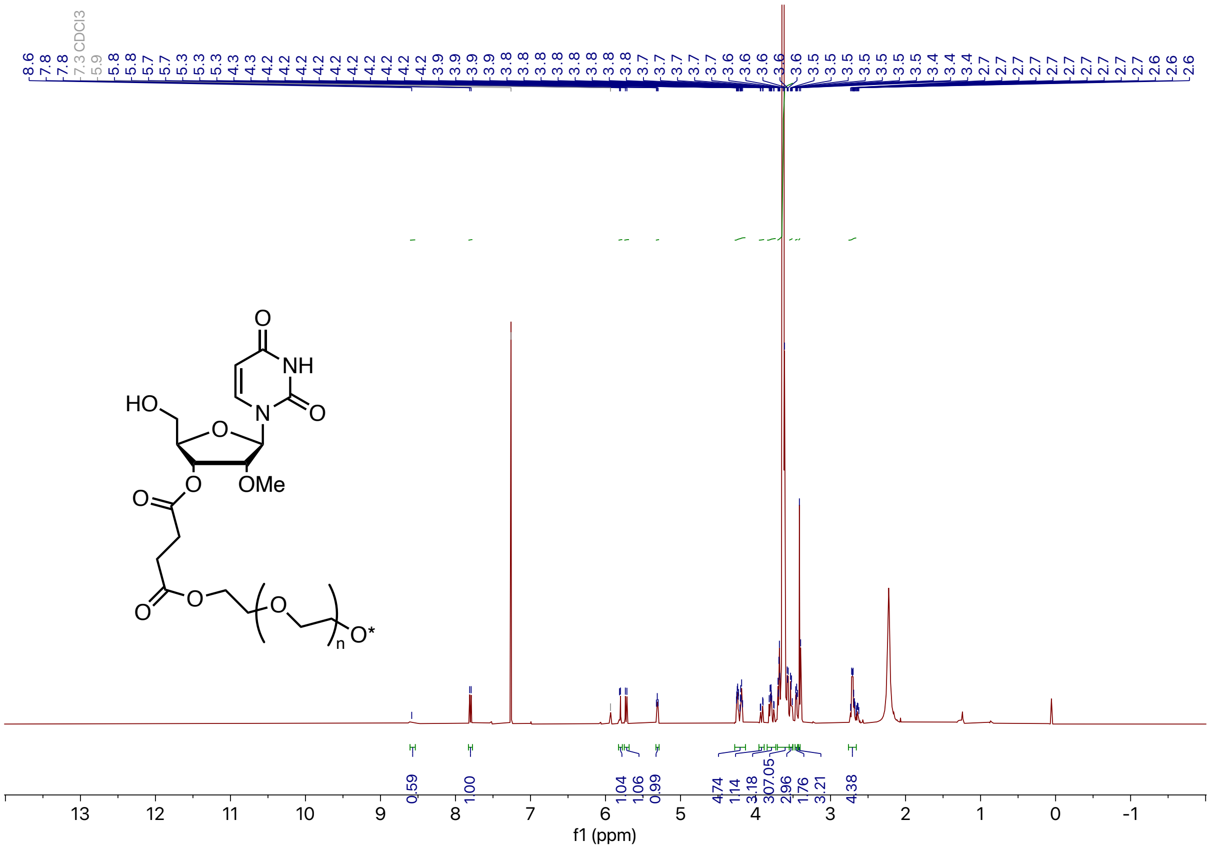

Figure S24: ^{1}H NMR spectra of 10 kDa 4-arm PEG-O-mU-OH, ***2c***

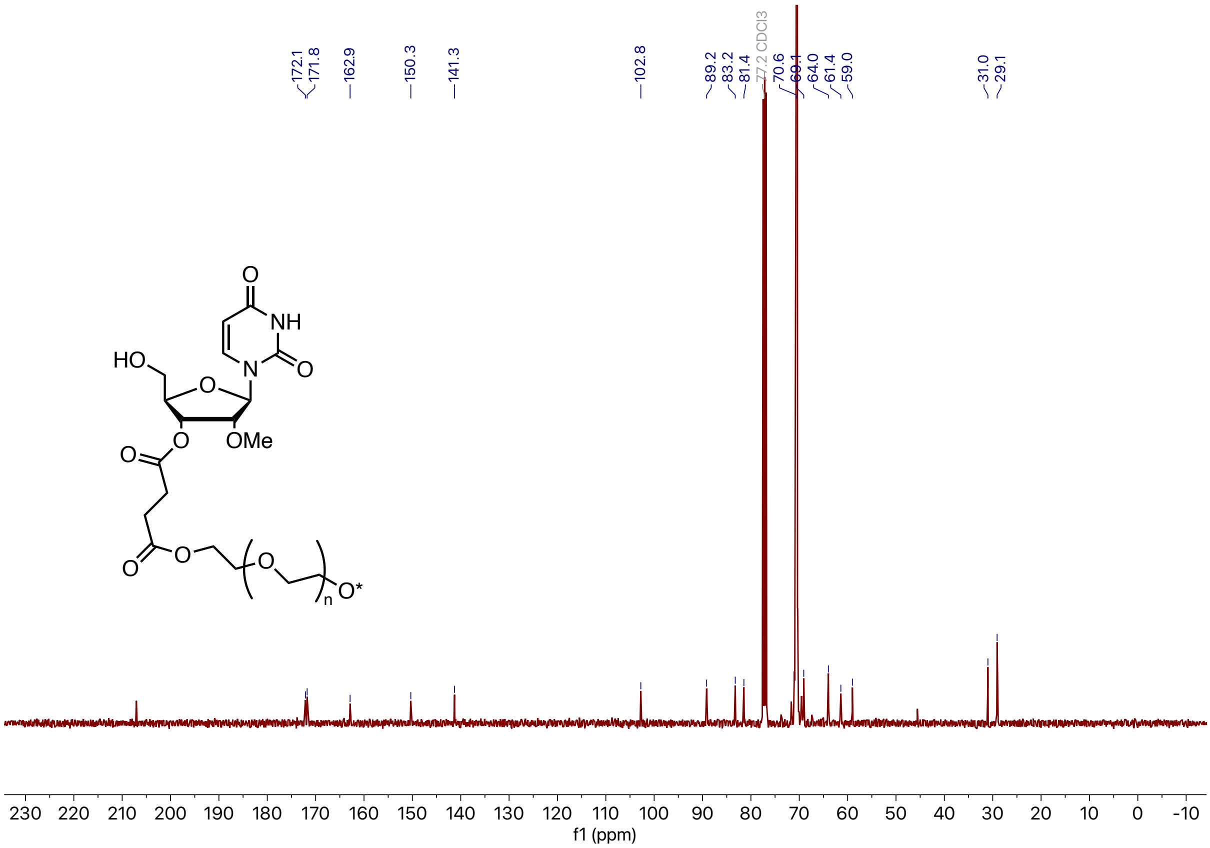

Figure S25: ^{13}C NMR spectra of 10 kDa 4-arm PEG-O-mU-OH, ***2c***

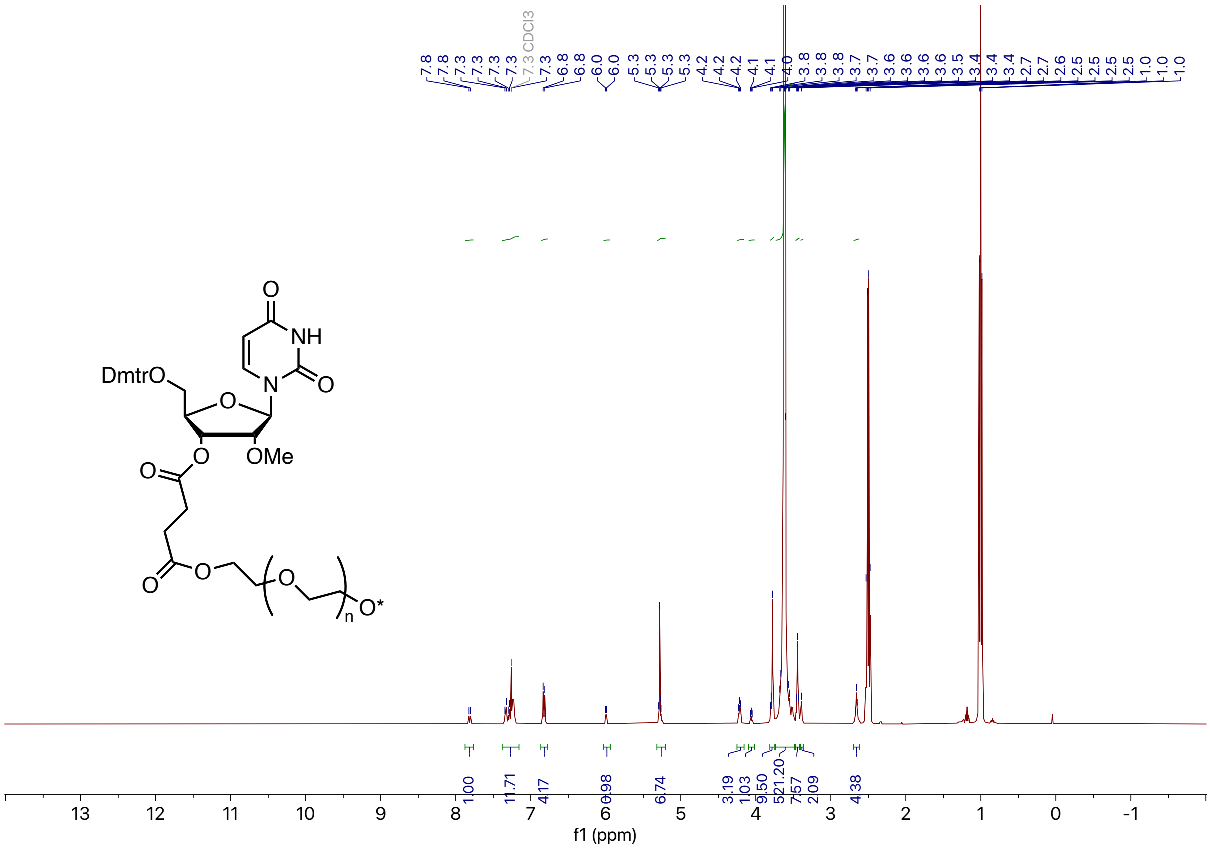

*Figure S26: ^{1}H NMR spectra of 20 kDa 4-arm PEG-O-mU-ODmtr, **3b***

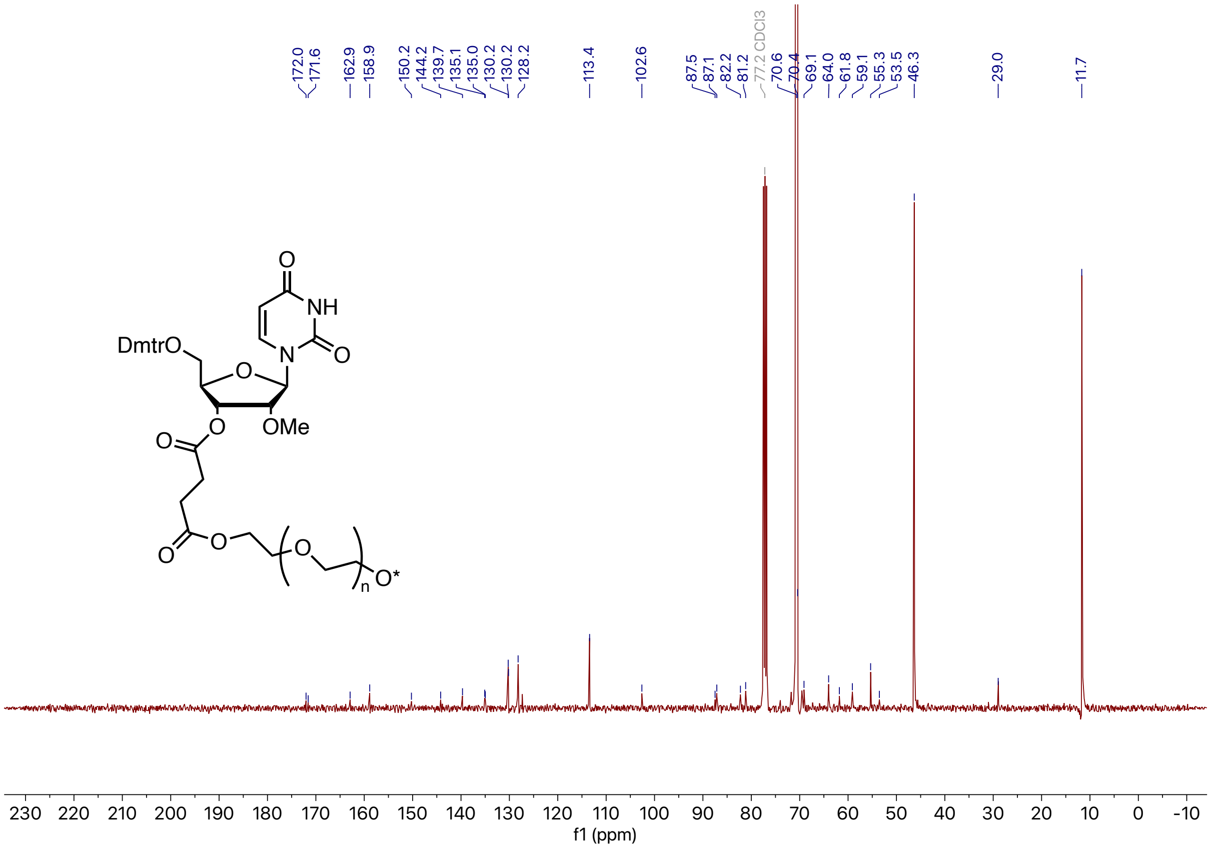

*Figure S27: ^{13}C NMR spectra of 20 kDa 4-arm PEG-O-mU-ODmtr, **3b***

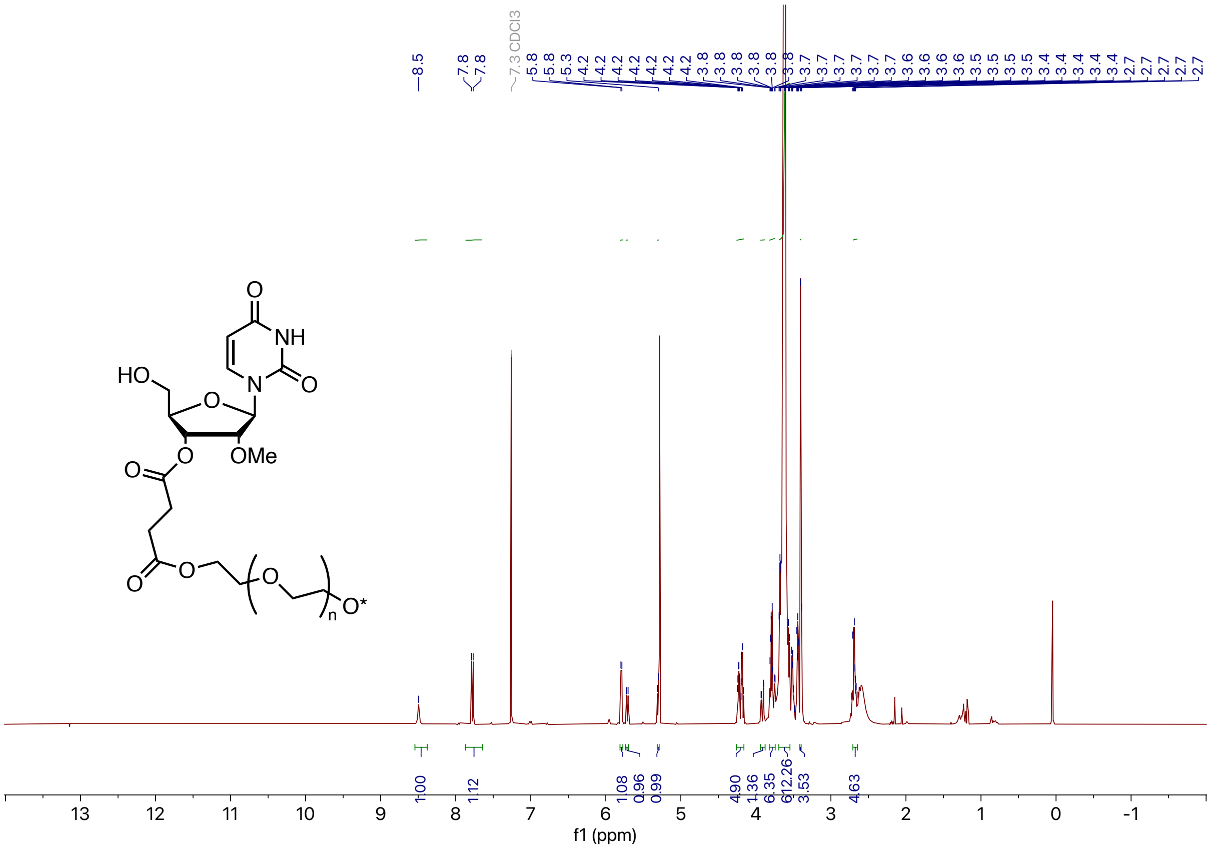

*Figure S28: ^{1}H NMR spectra of 20 kDa 4-arm PEG-O-mU-OH, **3c***

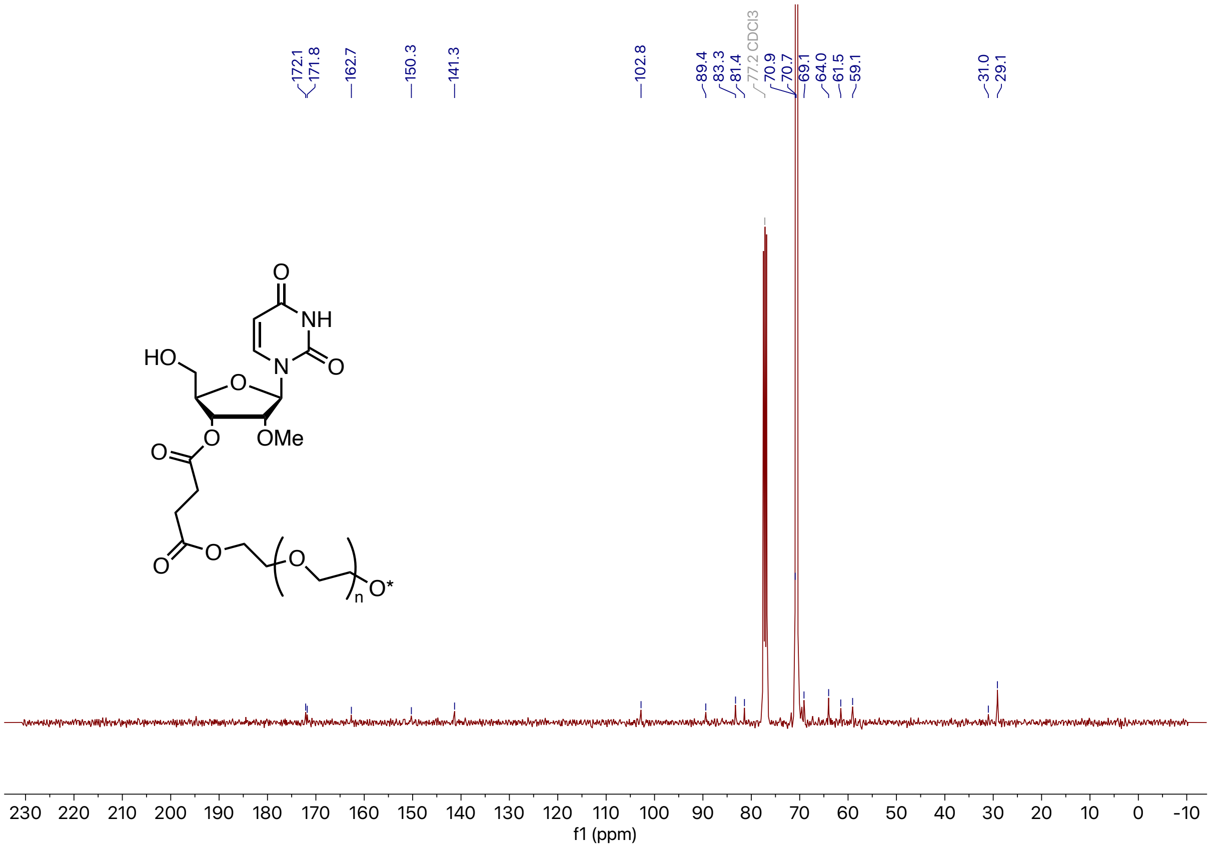

*Figure S29: ^{13}C NMR spectra of 5 kDa 4-arm PEG-O-mU-OH, **3c***

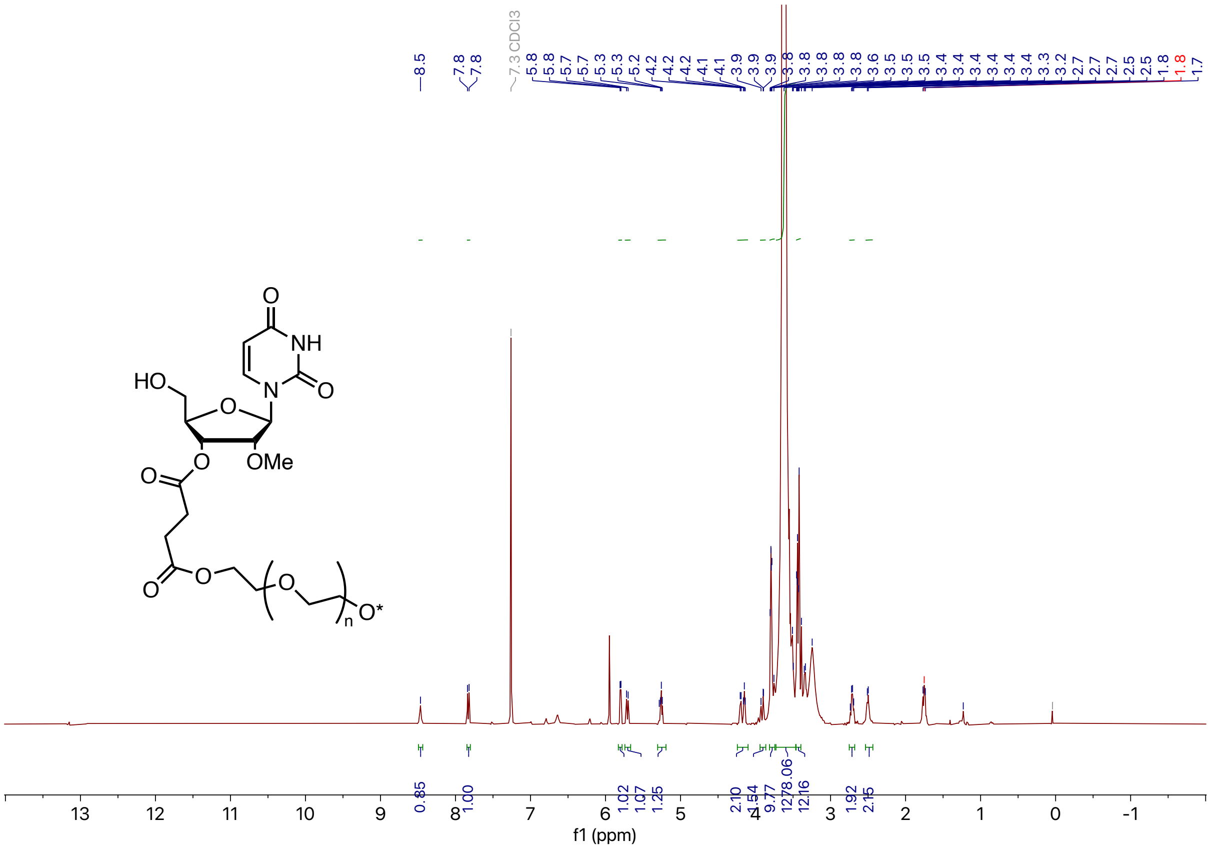

*Figure S30: ^{1}H NMR spectra of 40 kDa 4-arm PEG-O-mU-OH, **4c***

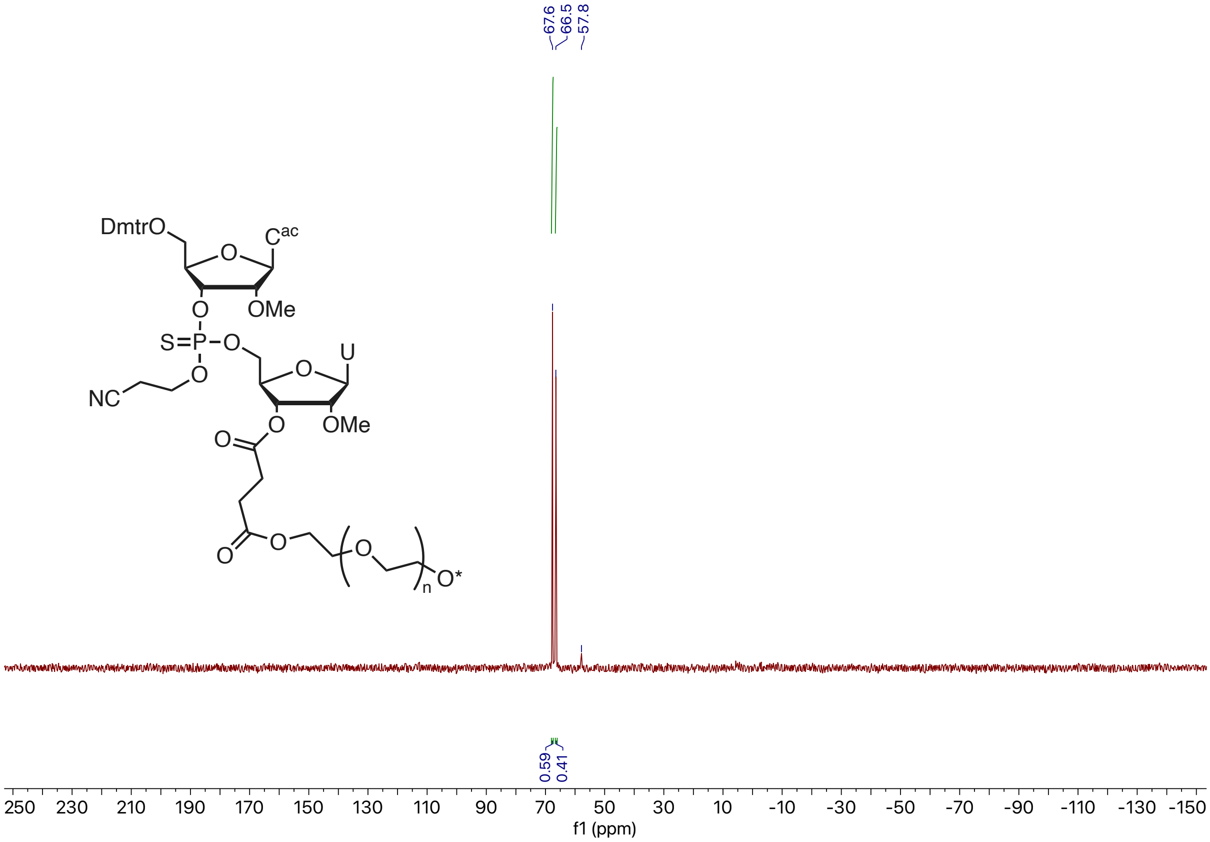

Figure S31: ^{31}P NMR spectra of dimer product after first round of diafiltration with NF 750 Da membrane post coupling-sulfurization showing product phosphorothioate and trace monothioate

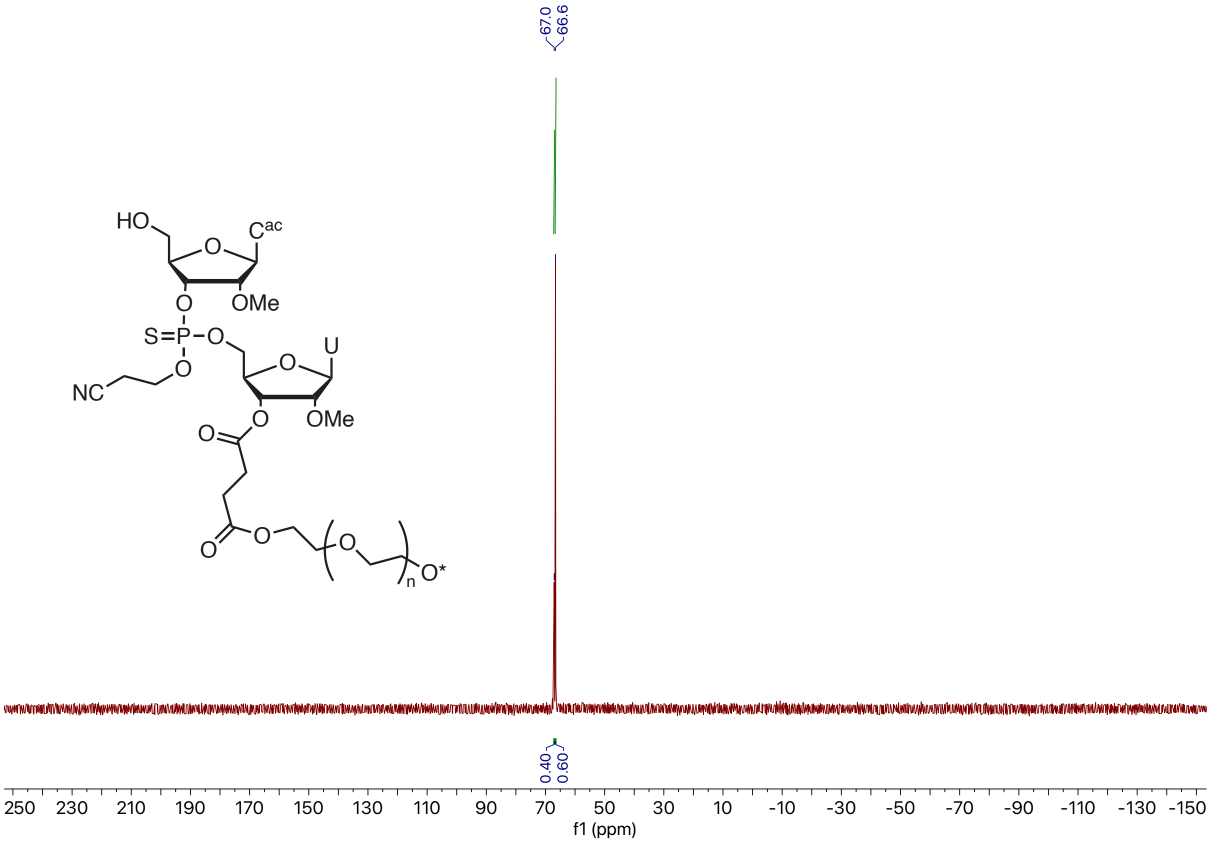

Figure S32: ^{31}P NMR spectra of dimer product after second round of diafiltration with NF 750 Da membrane post detritylation showing product phosphorothioate

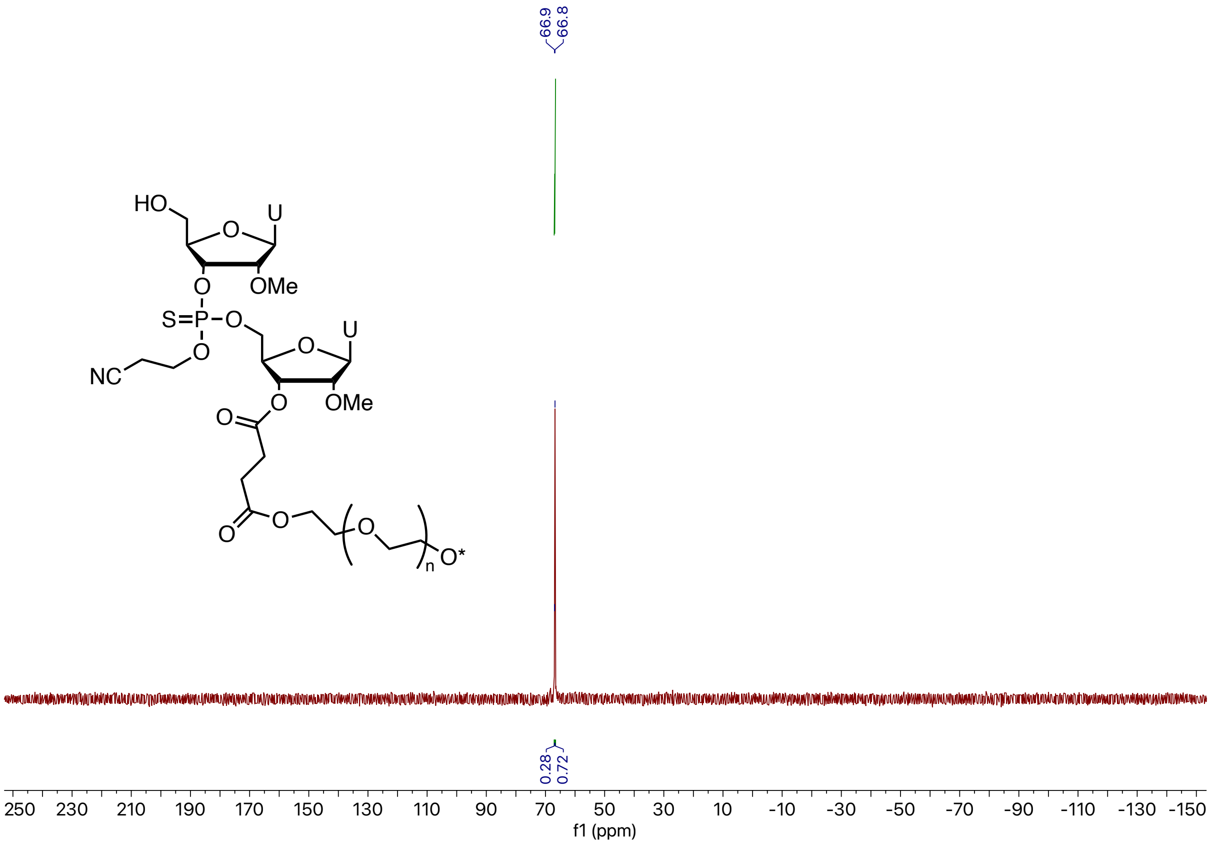

*Figure S33: ^{31}P NMR spectra of dimer product **1d*** after diafiltration with NF 750 Da membrane

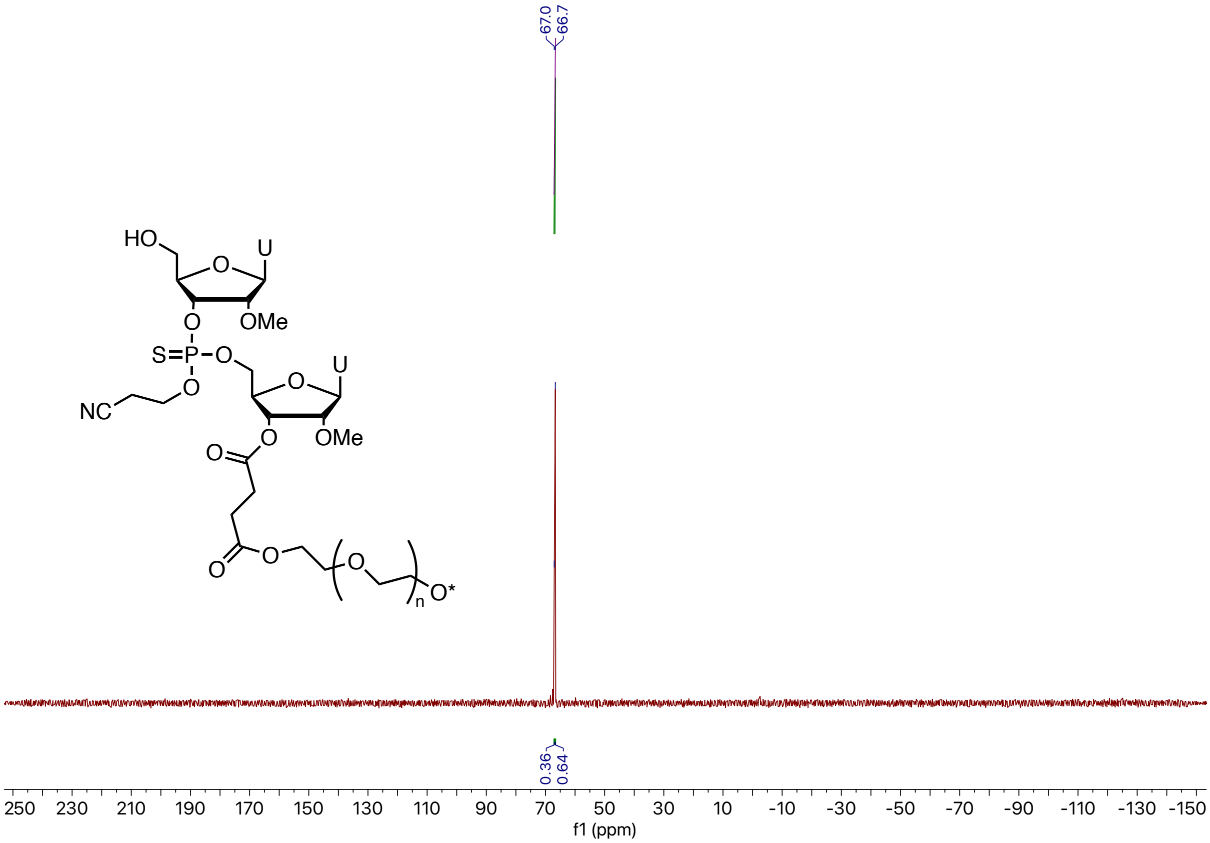

*Figure S34: ^{31}P NMR spectra of dimer product **3d*** after diafiltration with UF 2000 Da membrane

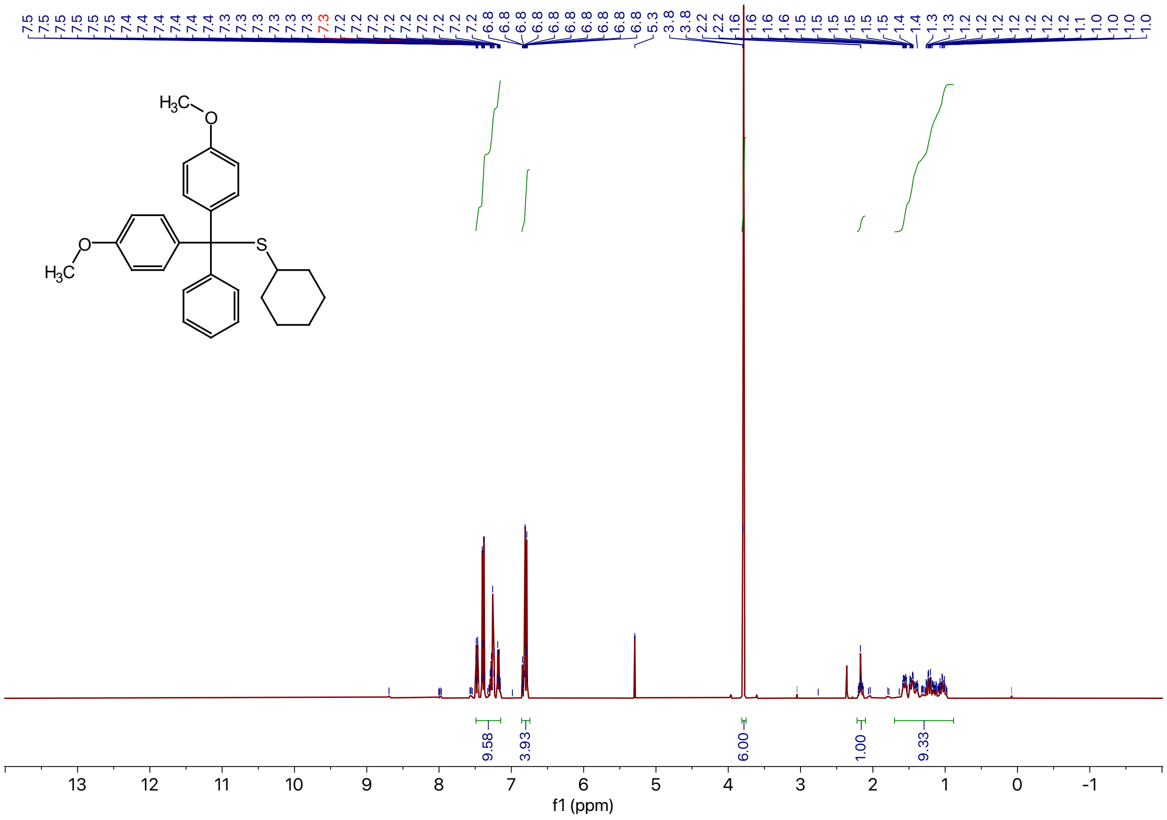

*Figure S35: ^{1}H NMR spectra of 4,4'-Dimethoxytrityl (cyclohexyl) sulfane, Dmtr-CySH**, 7***

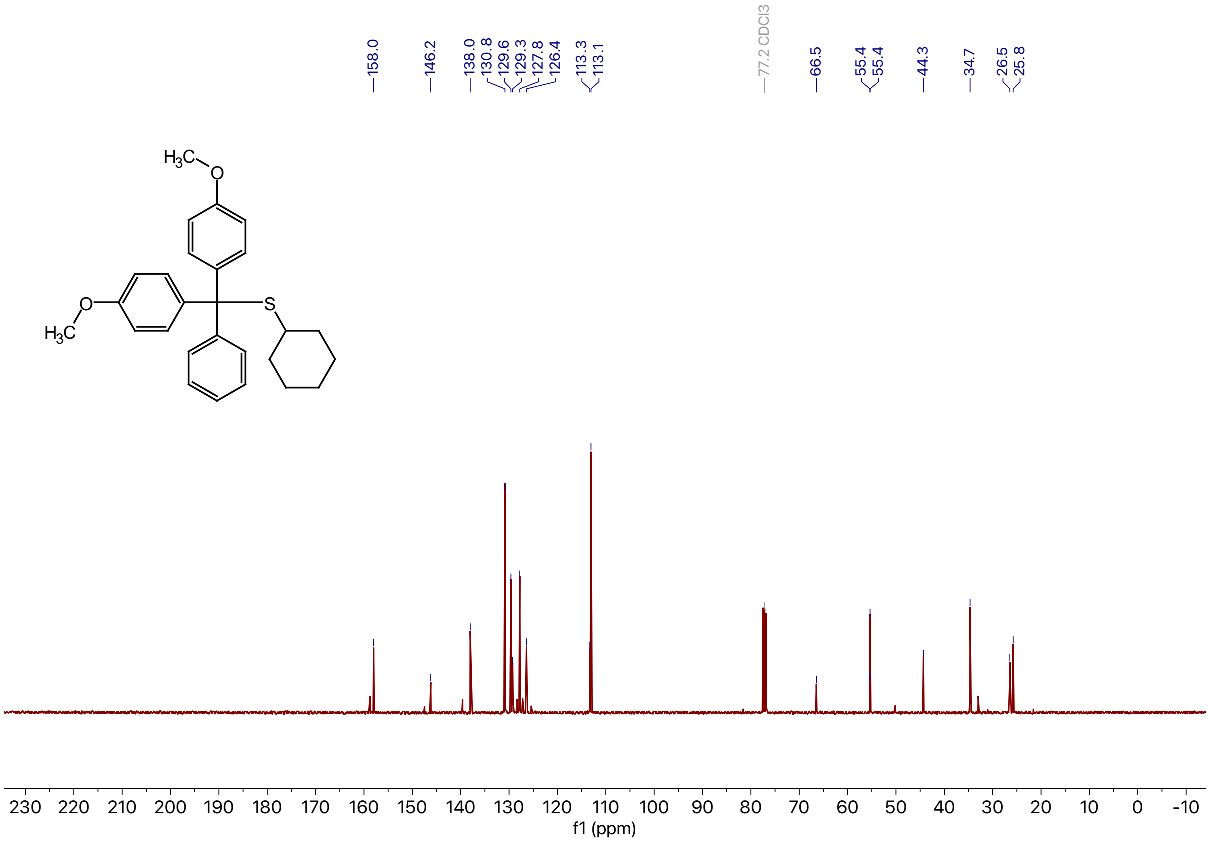

*Figure S36: ^{13}C NMR spectra of 4,4'-Dimethoxytrityl (cyclohexyl) sulfane, Dmtr-CySH, **7***

MALDI-TOF

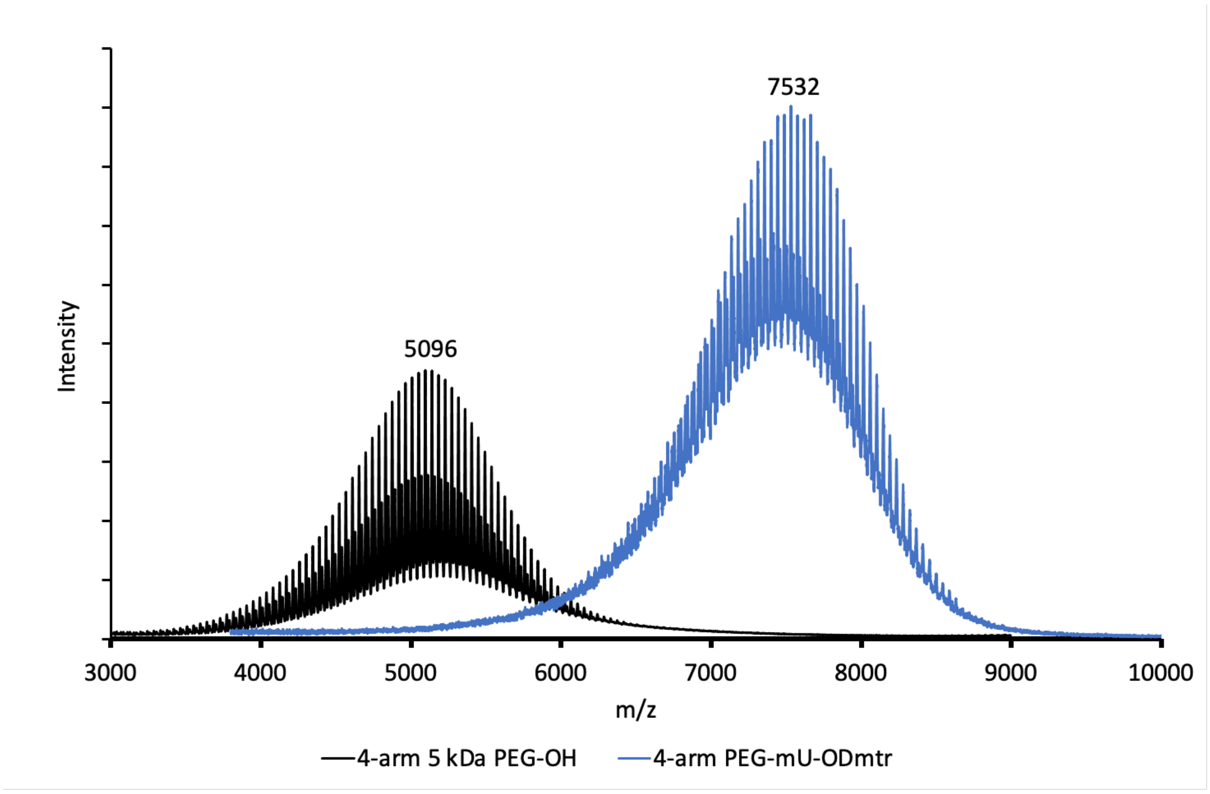

*Figure S37: MALDI-TOF spectrum of unloaded 4-arm 5 kDa PEG-OH **1a** and loaded 4-arm 5 kDa PEG-mU-ODmtr **1b** with average MW indicated before and after loading.*

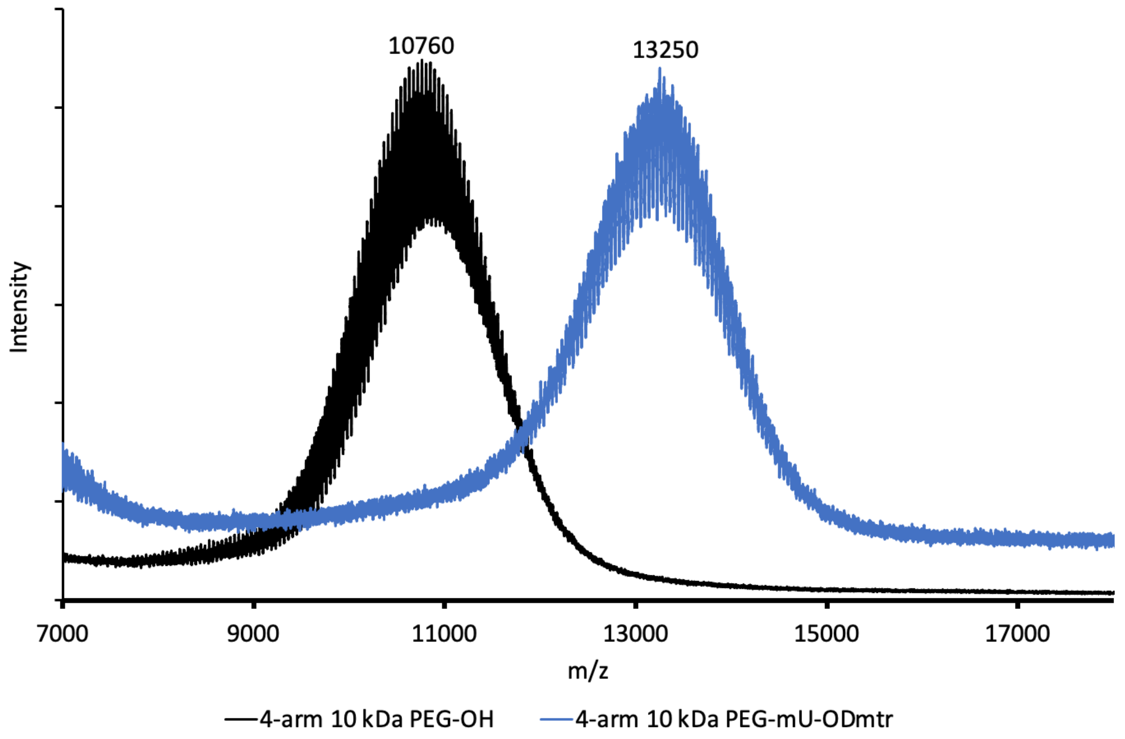

*Figure S38: MALDI-TOF spectrum of unloaded 4-arm 10 kDa PEG-OH **2a** and loaded 4-arm 10 kDa PEG-mU-ODmtr **2b** with average MW indicated before and after loading.*

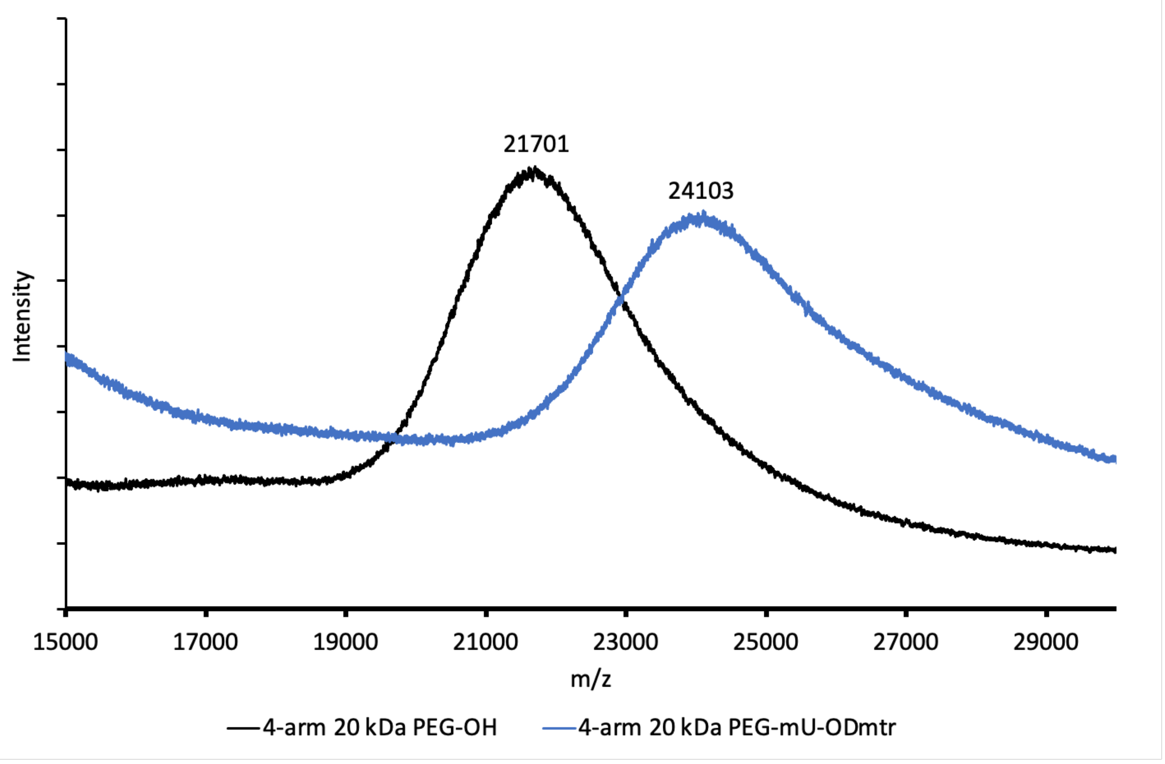

*Figure S39: MALDI-TOF spectrum of unloaded 4-arm 20 kDa PEG-OH **3a** and loaded 4-arm 20 kDa PEG-mU-ODmtr **3b** with average MW indicated before and after loading.*

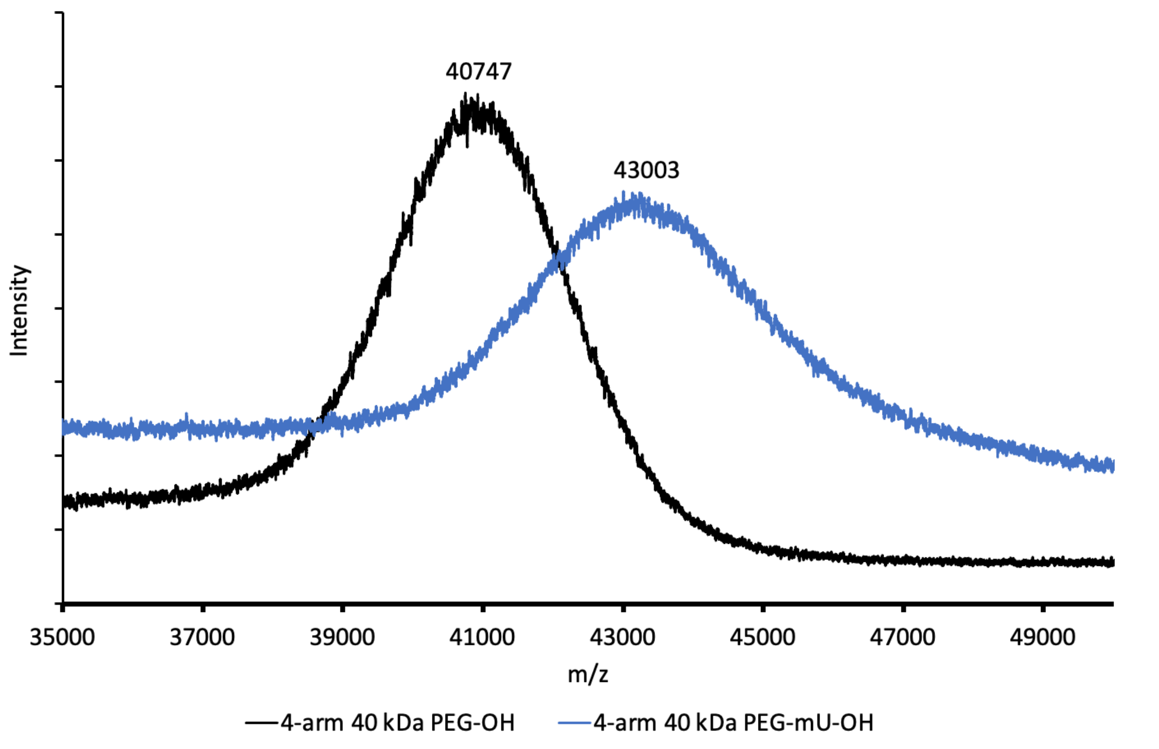

*Figure S40: MALDI-TOF spectrum of unloaded 4-arm 40 kDa PEG-OH **4a** and loaded 4-arm 40 kDa PEG-mU-ODmtr **4b** with average MW indicated before and after loading.*
